# GAMclust: identification of regulated metabolic modules in bulk, single cell and spatial gene expression data

**DOI:** 10.1093/bioinformatics/btag639

**Published:** 2026-08-22

**Authors:** Anastasiia Gainullina, Evgeniia Chikina, Maxim N Artyomov, Alexey Sergushichev

**Affiliations:** Computer Technologies Department, ITMO University, St. Petersburg, 197101, Russia; Laboratory of Bioinformatics and Molecular Genetics, Koltzov Institute of Developmental Biology of the Russian Academy of Sciences, Moscow, 119334, Russia; Life Improvement by Future Technologies (LIFT) Center, Moscow, 121205, Russia; Department of Pathology and Immunology, Washington University School of Medicine, St. Louis, MO 63110, United States; Department of Pathology and Immunology, Washington University School of Medicine, St. Louis, MO 63110, United States

## Abstract

**Motivation:**

Metabolism operates as a highly interconnected biochemical network, and its regulation emerges from coordinated changes across many reactions and metabolites. The integration of gene expression profiling data with organism-scale metabolic networks has proven to be a valuable tool for understanding cellular metabolic regulation. However, the increasing complexity of profiling technologies and experimental designs requires the development of specialized tools.

**Results:**

Here, we present GAMclust, an R package implementing and extending the previously published GAM-clustering pipeline for identifying transcriptionally regulated metabolic modules in complex gene expression datasets. GAMclust supports bulk, single-cell, and spatial gene expression profiling. It includes built-in KEGG and Rhea metabolic networks for human and mouse, with options to expand these networks for the analysis of other species. The package also offers a suite of post-processing and visualization tools, facilitating the exploration and interpretation of results.

**Availability:**

GAMclust is freely available at https://github.com/alserglab/GAMclust and https://doi.org/10.5281/zenodo.21432552 under the MIT license. Documentation is available at https://alserglab.github.io/GAMclust. Source code for supplementary materials is available at https://github.com/alserglab/GAMclust-paper.

## 1 Introduction

Metabolism is a highly complex system involving numerous interconnected processes of metabolite transformation, and it is actively studied using diverse experimental and computational methods (Swainston *et al.* 2016, Robinson *et al.* 2020, Specht *et al.* 2021, Kokaji *et al.* 2022). Bulk, single-cell, and spatial gene expression data provide valuable insights into cellular and tissue metabolism by quantifying the expression of enzymes that catalyze biochemical reactions (Kamburov *et al.* 2011, Kuo *et al.* 2013, Chong *et al.* 2018). However, because metabolic regulation is multilayered, changes in individual gene expression do not necessarily correspond to changes in metabolic fluxes. To address this, integrative analyses that connect gene expression changes at the pathway or whole-metabolism level are required (Beisser *et al.* 2010, He *et al.* 2017).

Metabolic network analysis methods approach this challenge by mapping expression data onto reaction networks, where reactions are linked through shared metabolites. Tools such as BioNet (Beisser *et al.* 2010), GAM (Sergushichev *et al.* 2016), and GATOM (Emelianova *et al.* 2022) can be used to identify an active metabolic module—a group of connected reactions where the corresponding enzymes are transcriptionally regulated within the dataset, for example, between control and treated samples. However, with the increase in dataset complexity, a single module becomes insufficient to properly describe the changes, requiring development of more specialized methods. For example, TiCoNE (Wiwie *et al.* 2019) was developed to find modules associated with time-course expression changes, whereas Compass (Wagner *et al.* 2021) enables single-cell level analysis.

Here, we introduce the GAMclust R package for identifying regulated metabolic modules in complex gene expression datasets. GAMclust implements and extends the GAM-clustering method, originally described and validated in a study of metabolic diversity in mouse mononuclear phagocytes across tissues (Gainullina *et al.* 2023). Unlike its predecessors, GAM (Sergushichev *et al.* 2016) and GATOM (Emelianova *et al.* 2022), GAM-clustering identifies multiple metabolic modules, each characterized by a distinct pattern of gene expression regulation. This enables the analysis of multi-condition bulk datasets, as well as single-cell and spatial data. Importantly, GAMclust operates in an unbiased fashion, independent of predefined reaction sets or pathway boundaries, offering a flexible and scalable approach to the data-driven discovery of metabolic modules. We demonstrate GAMclust on diverse datasets—including bulk, single-cell, and spatial RNA-sequencing studies of metabolic heterogeneity in physiological and tumor tissues—where it reveals both conserved and context-specific modules that recapitulate known biology and uncover novel metabolic dependencies.

## 2 GAMclust pipeline

**Figure 1:**
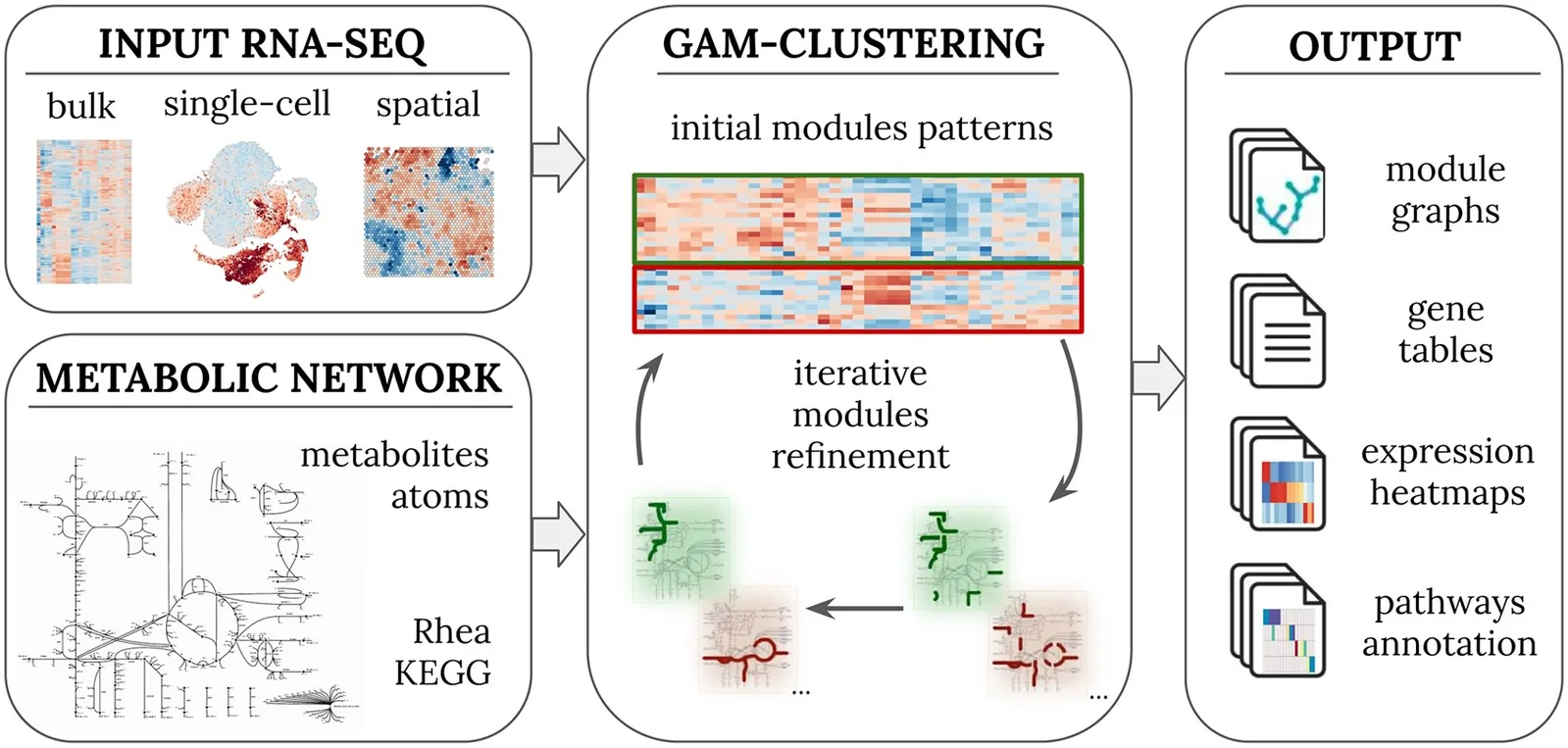
Overview of the GAMclust R-package workflow. Input gene expression data undergo preprocessing, including initial clustering. The initial expression patterns and the network are then passed to the core component—the GAM-clustering algorithm—which iteratively identifies metabolic modules that capture transcriptional variability within the analysed dataset. The output includes: (i) metabolic module graphs, (ii) tables of module-associated genes, (iii) module gene expression heatmaps, and (iv) pathway enrichment annotations.

The *GAMclust* R package implements a flexible data processing pipeline (Fig. 1) designed to analyse three major types of gene expression data: bulk, single-cell, and spatial. The pipeline takes as an input a gene expression matrix and a global metabolic network. After initial preprocessing, both of these inputs are integrated by the GAM-clustering algorithm, which identifies metabolic subnetworks (modules) with correlated gene expression behaviors. To aid in the interpretation of the results, several post-processing procedures are carried out, including visualization and annotation of the modules.

An important preprocessing step for the gene expression matrix is a low-rank approximation using principal component analysis (PCA). Projecting the input matrix to a low-dimension space reduces noise and makes true gene-gene correlations more pronounced. This is critically important for single-cell and spatial datasets due to their sparsity and higher overall noise, but can be beneficial for bulk gene expression datasets with a large number of samples as well. As an additional benefit, the low-rank approximation enables efficient storage of the matrix, reducing the memory requirements.

Next, the pre-processed expression data is integrated with a comprehensive *global metabolic network*—which describes all possible reactions in the organism. To construct a *dataset-specific* metabolic network, reactions from the global network that cannot be supported by the expressed genes of the dataset are filtered out. This involves mapping the genes to their corresponding enzymes and then to the associated metabolic reactions. The set of supported reactions is then represented as a graph, where edges correspond to reactions and vertices correspond either to metabolites or atoms, which get transformed by the corresponding reactions. Finally, the largest connected component of the graph is extracted to eliminate small disconnected fragments.

The above steps provide the necessary inputs for the main procedure of module identification (panel “GAM-CLUSTERING” in Fig. 1). This procedure is realized as an iterative module refinement and should be initialized with a set of expression patterns that serve as seeds for finding metabolic modules. By default, these initial expression patterns are obtained by performing k-means clustering on the full or PCA-reduced gene expression matrix. The initial patterns then serve as references against which the expression profile of every gene in the dataset is compared and scored. The score is defined in such a way that it is positive only if the gene is highly and specifically correlated with the given pattern (see Supplementary File 1 for the detailed formula). These scores are then used as weights for the edges of the dataset-specific metabolic network. Using a specialized solver for the Signal Generalized Maximum Weight Connected Subgraph problem (Emelianova *et al.* 2022), highly scored connected subgraphs are identified for each pattern. These subgraphs represent a new iteration of the modules, and their genes are used to recalculate more accurate expression patterns for each module, after which the iteration repeats. This process continues until the module patterns no longer change significantly between iterations and a set of modules satisfies several quality criteria, such as the absence of very large modules and modules with highly correlated patterns. A more detailed description of the algorithm and its parameters is available in Supplementary File 1 or in documentation at https://alserglab.github.io/GAMclust/. While changing of the algorithm parameters can be used to adjust the GAMclust results, the core results are stable between algorithm runs (Supplementary File 2).

At the final stage, a comprehensive report is generated to facilitate the interpretation of the results. It includes visualization of the final metabolic modules, the corresponding gene expression patterns, and annotations of the modules with canonical metabolic pathways from KEGG (Kanehisa and Goto 2000) and Reactome (Milacic *et al.* 2024) databases.

## 3 Case studies

To demonstrate the application of GAMclust to real data, we tested it across three distinct datasets containing bulk, single-cell, and spatial transcriptomics data (Supplementary Files 2–4). On the bulk ImmGen (Supplementary File 2) and single-cell Tabula Muris datasets (Supplementary File 3), GAMclust successfully reproduced the established biological findings, effectively recapitulating the core results of the original analyses (Gainullina *et al.* 2023). For the spatial transcriptomics glioblastoma dataset (Supplementary File 4), the algorithm identified hypoxic regions—a key spatial structure that was also described by the original authors of the dataset (Ravi *et al.* 2022). Notably, GAMclust captures global metabolic differences within the input samples, so it should be applied on a level appropriate to the biological question: for example, analysis of the bulk ImmGen and single-cell Tabula Muris datasets included only mononuclear phagocyte subset, while the spatial dataset was analysed as a whole.

Additionally, we applied GAMclust to an IDH-mutant glioma single-cell RNA-seq dataset (Friedrich *et al.* 2021) (Supplementary File 5). For this dataset, GAMclust identified two modules associated with the tumor phenotype and one module describing cell subpopulation without clear condition specificity. Both modules are mainly related to energy metabolism, with Module 1 highlighting the principal difference between IDH-wt and IDH-mutant tumors. IDH-wt gliomas, which are associated with poor prognosis, exhibit upregulation of the TCA cycle (Han *et al.* 2020), a metabolic feature captured by GAMclust. Module 2 reflected glycolytic metabolism and appeared to be more active in tumor samples compared to control samples, consistent with the well-known observation that tumor cells often rely on enhanced glycolysis as a more readily available energy source. Module 3 was related to sphingolipid metabolism and highlighted metabolic properties of one of the subpopulations.

To place the GAMclust in the context of complementary metabolic analysis approaches, we applied several related tools—TiCoNE (Wiwie *et al.* 2019), Compass (Wagner *et al.* 2021), and scCellFie (Armingol *et al.* 2025)—to the same glioma dataset (Supplementary Files 6–8). While the analysis by these methods also support the regulation of glycolysis and the TCA cycle, there are major differences in their usage. Similarly to GAMclust, TiCoNE uses a framework of identifying metabolic modules with distinct patterns of gene expression regulation, but, unlike GAMclust, is not as automated and highly depends on interaction with the user: TiCoNE’s main working mode requires the user to intervene during every round of the iterative clustering by manually splitting, merging or deleting the emerging cluster patterns. Compass also integrates a metabolic network with scRNA-seq data, but performs a different task: inferring reaction fluxes within individual cells using genome-scale metabolic modeling. Consequently, it does not provide tools to investigate connections between the reactions except for aggregating them into canonical pathways. Similarly, scCellFie analyses single cells relying on predefined metabolic tasks. Compared to Compass and scCellFie, GAMclust facilitates analysis of connections between different parts of the metabolic network, without reliance on canonical pathways, but does not fully enforce the flux structure. Across the tested datasets, GAMclust’s memory footprint ranged from roughly 4 to 16 GB depending on input data size, with a typical runtime of about one hour.

## 4 Conclusion

Metabolic networks are inherently complex, and interpreting transcriptional changes within their interconnected biochemical context requires integrative analytical tools. The GAMclust R package addresses this challenge by enabling unbiased identification of transcriptionally regulated metabolic modules across bulk, single-cell, and spatial gene expression datasets. Unlike other approaches such as Compass, TiCoNE, or scCellFie, GAMclust is fully automated and is not restricted to the predefined pathway boundaries, allowing the discovery of both known and novel metabolic pathways. GAMclust is open source and freely available at https://github.com/alserglab/GAMclust.

## Supplementary Material

btag639_Supplementary_Data

## Data Availability

GAMclust source code is available at https://github.com/alserglab/GAMclust and https://doi.org/10.5281/zenodo.21432552 under the MIT license. Documentation is available at https://alserglab.github.io/GAMclust. Source code for supplementary materials is available at https://github.com/alserglab/GAMclust-paper.
