# Supplementary material for: GAMclust: identification of regulated metabolic modules in bulk, single cell and spatial gene expression data
Source: Bioinformatics. 2026 Aug 22;42(9):btag639. doi: 10.1093/bioinformatics/btag639 (PMC13544963; doi:10.1093/bioinformatics/btag639)
Supplement: Supplementary file 1 [file btag639_supplementary_data.zip › S1_GAM-clustering_algorithm_description.pdf]

Supplementary File 1: GAM-clustering algorithm description

The file contains the pseudocode of the GAM-clustering algorithm and the description of the helper functions.

Algorithm 1: GAM-clustering

```
Input:
• Gene expression matrix E of size n × m
• Network edge table network
• List of k initial patterns cur.patterns of length m
• Parameters: start.base, base.dec, max.module.size, cor.threshold, p.adj.val.threshold
Result:
• Final metabolic modules

base ← start.base
while true do
    for j ∈ ℕ do
        k' ← |cur.patterns|
        revisions ← {}
        dist ← calculateCorrelationDistances(E, cur.patterns)
        for i ∈ {1, . . . , k'} do
            scores[i] ← calculateGeneScores(dist, i, base)
            mwcs[i] ← makeMwcsInstance(network, scores[i])
            modules[i] ← solveMwcs(mwcs[i])
            new.patterns[i] ← calculatePatterns(modules[i])
        end
        cur.patterns ← new.patterns
        revisions[j] ← cur.patterns
        if not changedSignificantly(cur.patterns, revisions[1..(j − 1)]) then
            break
        end
    end
    hasBig ← areThereAnyTooBigModules(modules, max.module.size)
    hasCorr, mc1, mc2 ← areThereAnyCorrelatedModules(modules, cor.threshold)
    hasUninform ← areThereAnyUninformativeModules(modules, p.adj.val.threshold)
    if hasBig then
        base ← base - base.dec * base
    end
    if hasCorr then
        cur.patterns ← mergeTwoModules(cur.patterns, mc1, mc2)
    end
    if hasUninform then
        maxOverlap, mu1, mu2 ← checkOverlap(modules)
        if maxOverlap > 0 then
            cur.patterns ← mergeTwoModules(cur.patterns, mu1, mu2)
        else
            cur.patterns[mu1] ← NULL
        end
    end
    if not any(hasBig, hasCorr, hasUninform) then
        break
    end
end
return modules
```

GAM-clustering helper functions

`calculateCorrelationDistances`(E, *cur.patterns*): computes correlation-based distances between gene expression profiles and module patterns. The correlation is converted to distance by subtracting it from 1. This results in a distance matrix where each entry represents how dissimilar a gene profile is from each module pattern.

`calculateGeneScores`(*dist*, *base*): transforms the distances for a specific module into a score for each gene, which will be used as edge weights in the MWCS problem. This score represents similarity of gene expression with the module's pattern and dissimilarity with other modules' patterns. Formally, score is defined as follows:

$$d\left(g_i, c_j\right) = 1 - \operatorname{cor}\left(g_i, c_j\right)$$

$$d\left(g_i, c_0\right) \equiv base$$

$$d'\left(g_i, c_j\right) = \min_{k \neq j, k \in (0, M)} \left(d\left(g_i, c_k\right)\right)$$

$$\operatorname{score}\left(g_i, c_j\right) = -\log \frac{d(g_i, c_j)}{d'\left(g_i, c_j\right)}$$

where g_i—expression of the i-th gene, $i \in (1, N)$;

c_j—pattern of the j-th cluster if $j \in (1, M)$ or special fake pattern if $j = 0$;

d—distance to the pattern the score is being calculated for;

d'—distance to the pattern which this gene has the most correlation with

(all other patterns are considered except the pattern the score is being calculated for);

base — distance to the fake pattern.

`makeMwcsInstance`(*network*, *scores*): formally defines an instance of the Maximum Weight Connected Subgraph (MWCS) problem. Takes the biological *network* (graph) and the *scores* vector for module, and outputs a formal MWCS problem object.

`solveMwcs`(*mwcs*): solves the MWCS instance for module to find a single connected subgraph where the sum of the edge weights is maximized. Returns a list of genes that form the connected subgraph for this module, which becomes the new refined module.

`calculatePatterns`(*modules*): recalculates the expression pattern for a newly identified module. Computes the mean of the expression profiles of all genes within the module across all conditions.

`changedSignificantly`(*cur.patterns*, *revisions*): determines if the algorithm has converged by checking if the module patterns have stopped changing significantly between iterations. Compares the current patterns against the patterns from previous iterations with the same number of modules in it.

`areThereAnyTooBigModules`(*modules*, *max.module.size*): returns *True* if any module contains more genes than the *max.module.size* parameter.

`areThereAnyCorrelatedModules`(*modules*, *cor.threshold*): identifies if any two distinct modules have become too similar to each other. Calculates the correlation between the expression patterns of every pair of modules. Returns *True* if any pair's correlation exceeds the *cor.threshold*. Also returns the pair of most correlated modules mc_1 and mc_2.

`areThereAnyUninformativeModules`(*modules*, *p.adj.val.threshold*): identifies modules that do not show statistically significant expression changes across the conditions (uninformative modules) by performing GESECA test from the `fgsea` package. Returns *True* if any module does not pass the multiple-testing corrected significance threshold (*p.adj.val.threshold*).

`checkOverlap`(*modules*): computes the overlap of positive-scoring genes between modules and identifies the uninformative module with the largest overlap to any other module. Returns the maximum overlap value *maxOverlap* and the corresponding modules mu_1 and mu_2.

`mergeTwoModules`(*cur.patterns*, mu_1, mu_2): merges two modules mu_1 and mu_2 into one by creating a new expression pattern from the combined set of genes from mu_1 and mu_2. The original patterns of mu_1 and mu_2 are removed. Returns the updated set of $|cur.patterns| - 1$ patterns.
