# Supplementary material for: GAMclust: identification of regulated metabolic modules in bulk, single cell and spatial gene expression data
Source: Bioinformatics. 2026 Aug 22;42(9):btag639. doi: 10.1093/bioinformatics/btag639 (PMC13544963; doi:10.1093/bioinformatics/btag639)
Supplement: Supplementary file 1 [file btag639_supplementary_data.zip › S2_Robustness_of_GAMclust_modules_to_parameter_choice_and_random_perturbations.pdf]

Supplementary file 2

Robustness of GAMclust modules to parameter choice and random perturbations.

This supplementary file describes tests evaluating reliability of GAMclust results. First, we used gene-label shuffling to test whether the detected modules could be explained by the metabolic network topology alone. We then tested whether the main modules were stable under different parameter settings.

Unless stated otherwise, the baseline analysis used the default GAMclust settings described in the corresponding vignettes. All upset plots show intersection sets with size ≥ 2.

1. Gene-label shuffling

To test whether modules could appear only because of the metabolic network structure, we performed gene-label shuffling in the bulk RNA-Seq ImmGen dataset. In this test, the network was kept unchanged, but gene labels were randomly reassigned. This breaks the link between gene expression profiles and metabolic reactions while preserving the network topology. We repeated this analysis for two values of max.module.size: 50 and 75.

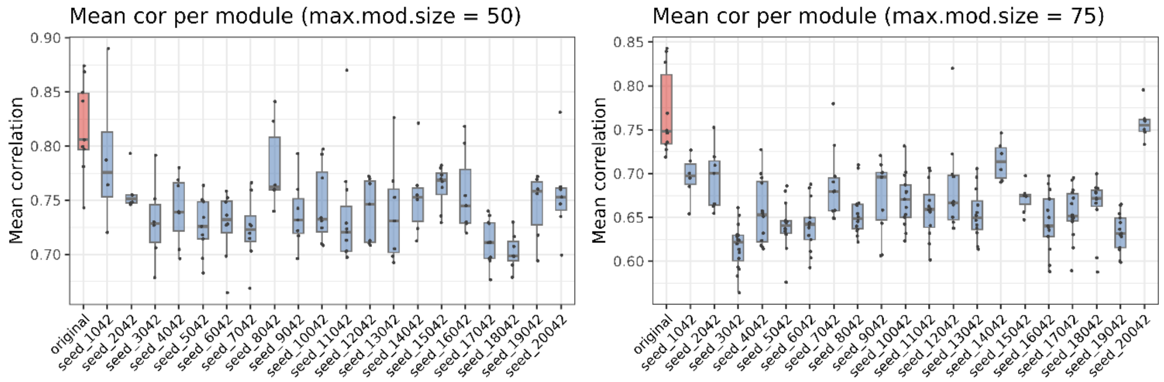

Figure 1. Gene-label shuffling analysis in the bulk RNA-seq dataset. Boxplots show mean correlation between module genes and their module pattern for the original data and gene-label shuffled data. The analysis was performed for two values of max.module.size: 50 (left) and 75 (right).

Modules from the original data showed stronger correlation with their module patterns than modules from shuffled data (**Fig. 1**). This proves that the inferred modules are not explained by network topology alone.

2. Maximum module size

This parameter controls how large the detected modules can be. We accessed the stability of inferred modules for the range of values from 50 to 75 for the bulk RNA-seq data analysis.

The main modules were recovered across all tested values and the core genes remained similar (**Fig. 2**). Larger values of max.module.size allowed GAMclust to include more genes in the modules, but the main module content remained similar. Smaller default values provide more compact and easier-to-interpret modules, whereas larger values may be useful for more detailed exploratory analyses.

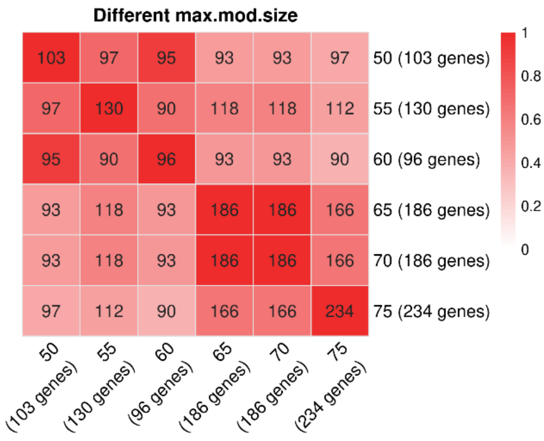

Figure 2. Effect of maximum module size in bulk RNA-seq data analysis. Heatmap showing overlap between modules inferred with different values of max.module.size. Color indicates Jaccard similarity, and numbers indicate the number of shared genes.

3. Number of initial clusters

GAMclust uses initial expression patterns obtained from k-means clustering of the input expression matrix. We tested whether the final modules depend on the number of initial clusters *k*. We tested values of *k* from 32 to 64 in bulk, single-cell, and spatial RNA-seq datasets.

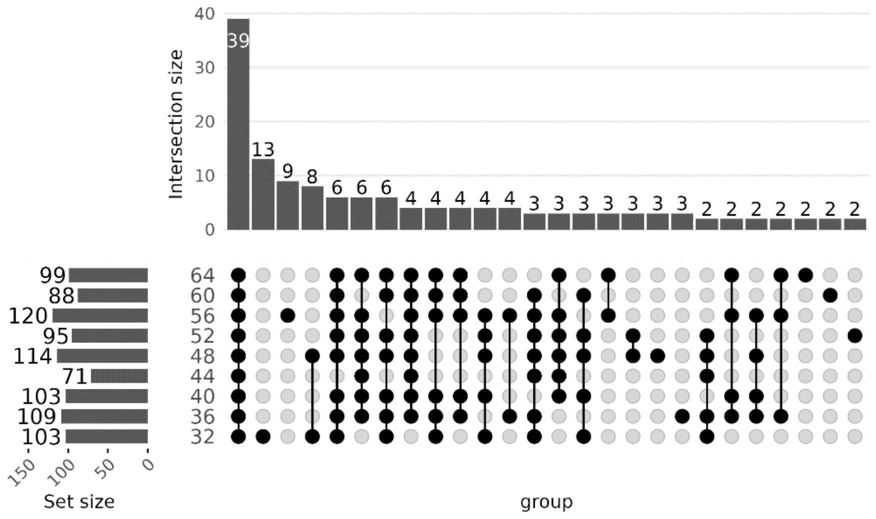

Figure 3. Effect of the number of initial clusters in bulk RNA-seq data analysis. UpSet plot showing overlap between module genes inferred with different values of k.

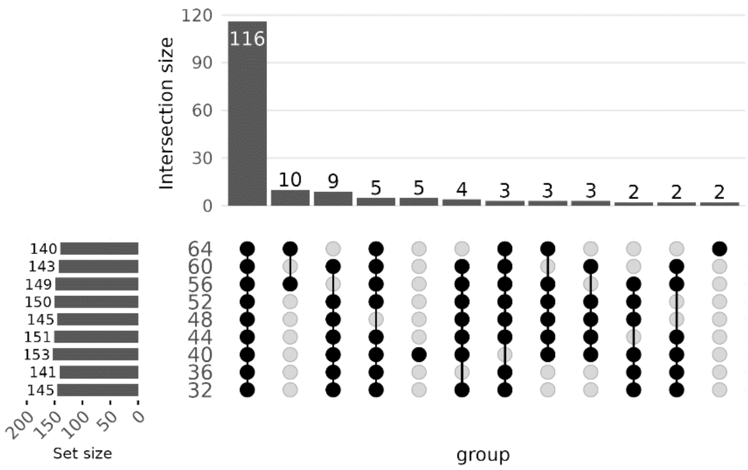

Figure 4. Effect of the number of initial clusters in single-cell RNA-Seq data analysis. UpSet plot showing overlap between module genes inferred with different values of k.

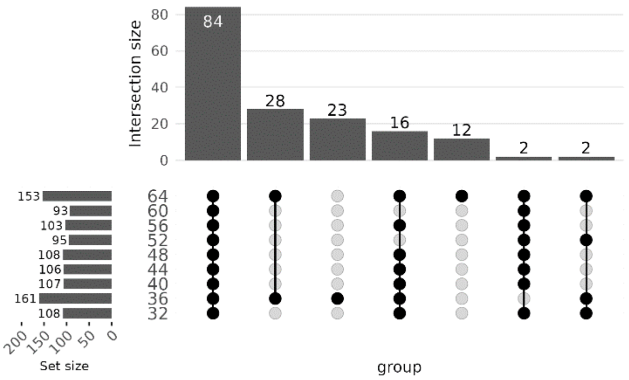

Figure 3. Effect of the number of initial clusters in spatial RNA-seq data analysis. UpSet plot showing overlap between module genes inferred with different values of k.

The main gene core found by GAMclust was stable across the tested values in all three datasets (**Fig. 3-5**). Overall, the final modules were stable across tested values and are not driven by one specific choice of the initial number of clusters.

4. Random seed

We repeated the analysis with different random seeds while keeping the default parameters for the bulk RNA-Seq dataset. This test mainly evaluates the effect of random k-means initialization, which is the main stochastic step in the pipeline. The analysis was repeated for two values of max.module.size: 50 and 75.

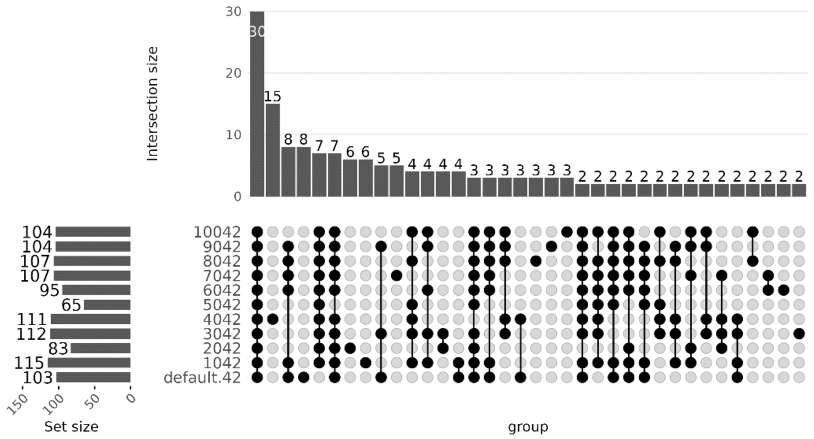

Figure 6. Effect of random seed with max.module.size = 50 in bulk RNA-Seq data analysis. UpSet plot showing overlap between module genes inferred with different random seeds.

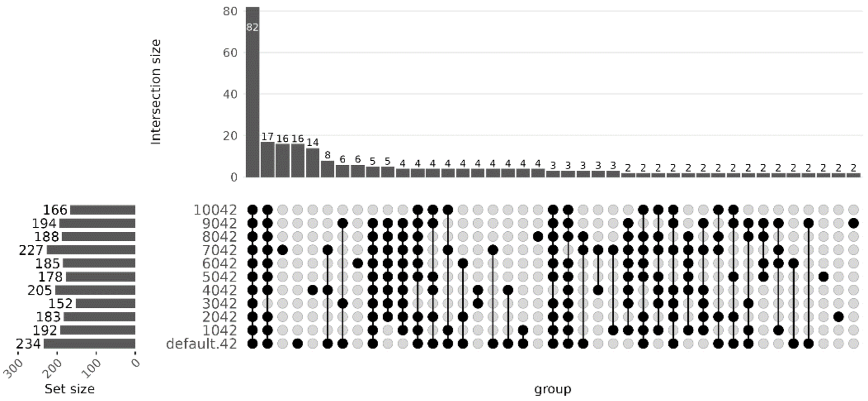

Figure 7. Effect of random seed with max.module.size = 75 in bulk RNA-Seq data analysis. UpSet plot showing overlap between module genes inferred with different random seeds.

In both cases, the main modules were reproduced across random seeds (**Fig. 6-7**). This shows that the main results do not depend on one specific random seed and can be reproduced.

5. Cell subsampling

For the single-cell dataset, we tested whether modules remain stable when fewer cells are used. We randomly selected 50% of cells and repeated the analysis 10 times.

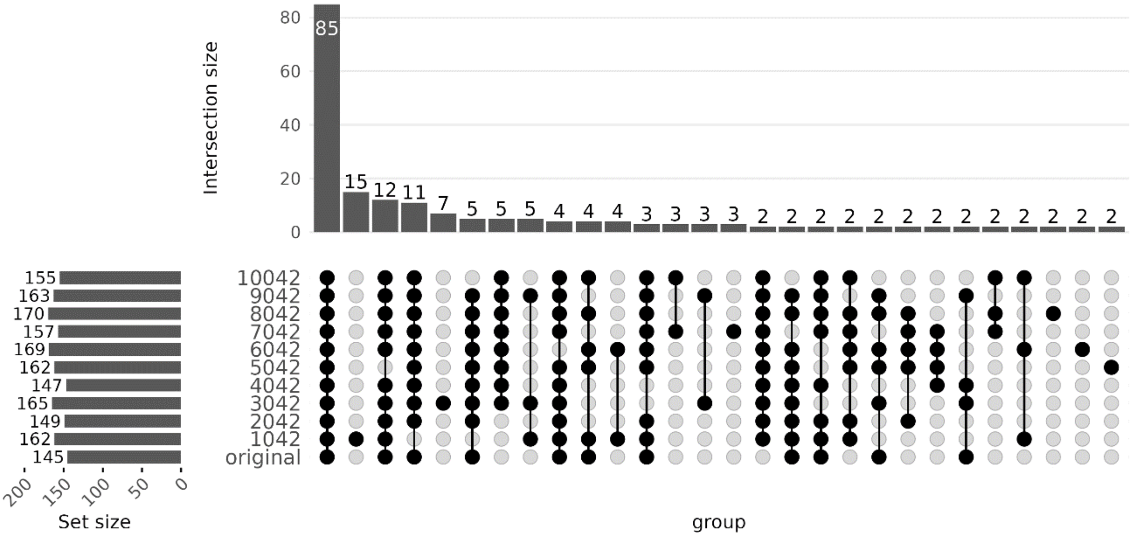

Figure 8. Cell subsampling analysis for single-cell RNA-Seq dataset. UpSet plot showing overlap between module genes inferred from the full (original) dataset and from 10 random subsamples containing 50% of cells.

The main modules were still recovered after subsampling, and their core genes remained stable across runs (Fig. 8). Smaller modules were more variable because they are more sensitive to changes in cell composition.

6. Number of principal components

For single-cell and spatial datasets, GAMclust uses PCA-based dimensionality reduction before module inference. We tested whether the final modules depend on the number of principal components. We repeated the analysis using from 10 to 50 principal components.

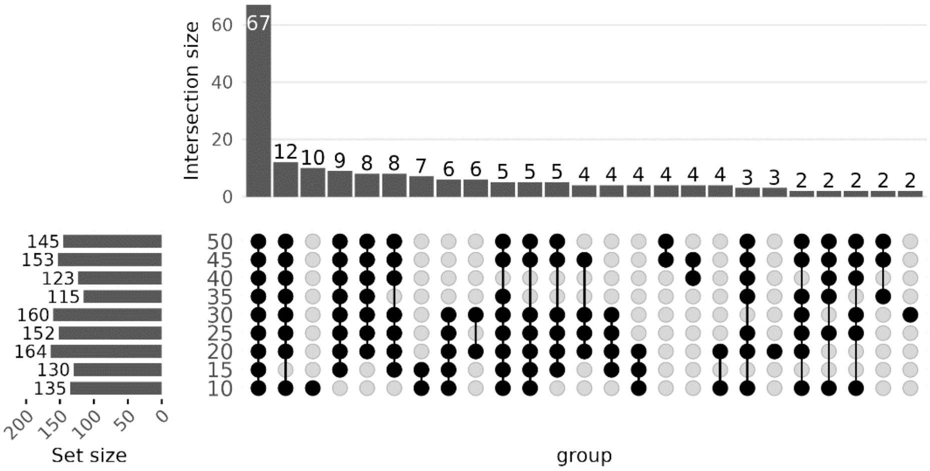

Figure 9. Effect of the number of principal components in single-cell RNA-Seq data analysis. UpSet plot showing overlap between module genes inferred with different numbers of principal components.

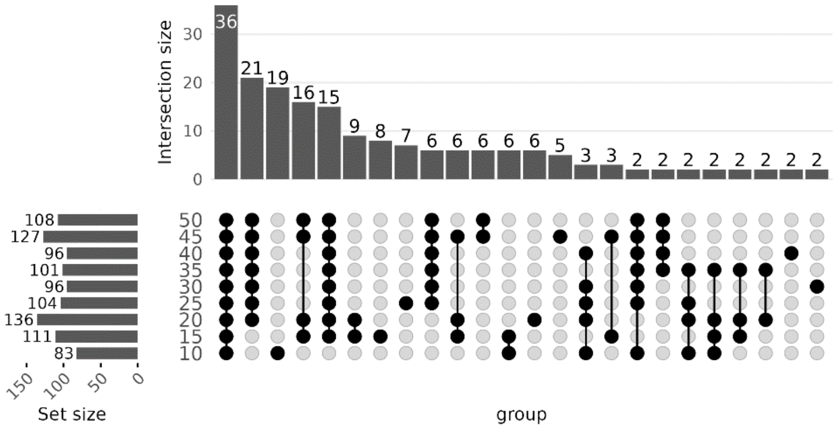

Figure 10. Effect of the number of principal components in spatial RNA-Seq data analysis. UpSet plot showing overlap between module genes inferred with different numbers of principal components.

Summary

We performed various tests to evaluate the stability and significance of GAMclust results.

The significance of detected modules is supported by gene-label shuffling experiments showing that the detected modules are not explained by the metabolic network topology alone.

Changing parameters such as maximal module size (max.module.size), the number of initial clusters (k), and the number of principal components preserved the core genes of the main modules, although smaller are less stable modules. Cell subsampling supports the robustness of the identified modules: they were recovered even in a subset of cells. At the same time, modules related to rare cell populations may be more sensitive to subsampling. The core module genes were also recovered across runs with different random seeds, suggesting that the choice of seed does not strongly affect main results.

Overall, these results support the use of the default GAMclust settings as a practical starting point. At the same time, we encourage users to explore different parameter settings for their own datasets. GAMclust vignettes provide guidance on which parameters can be adjusted and how these changes may affect the analysis.
