# Supplementary material for: GAMclust: identification of regulated metabolic modules in bulk, single cell and spatial gene expression data
Source: Bioinformatics. 2026 Aug 22;42(9):btag639. doi: 10.1093/bioinformatics/btag639 (PMC13544963; doi:10.1093/bioinformatics/btag639)
Supplement: Supplementary file 1 [file btag639_supplementary_data.zip › S3_GAMclust_tutorial_BULK.pdf]

GAM-clustering reanalysis of ImmGen Open Source bulk RNA-seq data

Introduction

This vignette walks over application of GAM-clustering method for reanalysis of metabolic regulation in ImmGen Open Source bulk RNA-seq data (mononuclear phagocyte subset), originally described in Gainullina et al.

The vignette requires about an hour of computational time and 4GB of memory for completion.

Installation and libraries

Install GAMclust package:

```
devtools::install_github("alserglab/GAMclust")
```

```
library(GAMclust)
library(gatom)
library(mwcsr)
library(fgsea)
library(data.table)
library(futile.logger)

set.seed(42)
```

```
startTime <- Sys.time()
```

Preparing working environment

First, load and initialize all objects required for GAM-clustering analysis:

(1) load metabolic network and its metabolites annotation. We provide two networks: KEGG and combined network that includes KEGG, Rhea and transport reactions:

(1.1.) load KEGG metabolic network `network.kegg.rds` and its metabolites annotation `met.kegg.db.rds`

```
# KEGG network:
network <- readRDS(url("http://artyomovlab.wustl.edu/publications/supp_materials/GATOM/ne
↪ twork.kegg.rds"))
metabolites.annotation <- readRDS(url("http://artyomovlab.wustl.edu/publications/supp_mat
↪ erials/GATOM/met.kegg.db.rds"))
```

(1.2.) or load combined metabolic network `network.combined.rds`, its metabolites annotation `met.combined.db.rds` and species-specific list of genes that either come from proteome or are not linked to a specific enzyme `gene2reaction.combined.mmu.eg.tsv` for mouse and `gene2reaction.combined.hsa.eg.tsv` for human data;

```
# combined network (KEGG+Rhea+transport reactions):
network <- readRDS(url("http://artyomovlab.wustl.edu/publications/supp_materials/GATOM/ne
↪ twork.combined.rds"))
```

```
metabolites.annotation <- readRDS(url("http://artyomovlab.wustl.edu/publications/supp_mat
↪  erials/GATOM/met.combined.db.rds"))
gene2reaction.extra <- data.table::fread("http://artyomovlab.wustl.edu/publications/supp_
↪  materials/GATOM/gene2reaction.combined.mmu.eg.tsv", colClasses="character")
```

(2) load species-specific network annotation: `org.Hs.eg.gatom.anno` for human data or `org.Mm.eg.gatom.anno` for mouse data;

```
network.annotation <- readRDS(url("http://artyomovlab.wustl.edu/publications/supp_materia
↪  ls/GATOM/org.Mm.eg.gatom.anno.rds"))
```

(3) load provided list of metabolites that should not be considered during the analysis as connections between reactions (e.g., CO2, HCO3-, etc);

```
met.to.filter <- data.table::fread(system.file("mets2mask.lst", package="GAMclust"))$ID
```

(4) initialize MWCS solver:

(4.1.) we recommend to use here either heuristic relax-and-cut solver `rnc_solver` from `mwcsr` package,

```
solver <- mwcsr::rnc_solver()
```

(4.2.) either proprietary CPLEX solver (free for academy);

```
cplex.dir <-  "/opt/ibm/ILOG/CPLEX_Studio1271"
```

```
solver <- mwcsr::virgo_solver(cplex_dir = cplex.dir)
```

(5) set working directory where the results will be saved to.

```
work.dir <- "results-bulk"
dir.create(work.dir, showWarnings = F, recursive = T)
```

Preparing objects for the analysis

Preparing data

GAMclust works with bulk, single cell and spatial RNA-seq data.

This vignette shows how to process bulk RNA-seq data on the example of ImmGen Open Source data reanalysis.

Let's load the data.

For bulk RNA-seq cell data, take 12,000-15,000 genes for the GAM-clustering analysis.

```
expression_set_object <- readRDS(url("http://artyomovlab.wustl.edu/publications/supp_mate
↪  rials/GAMclust/243_es.top12k.rds"))

E <- Biobase::exprs(expression_set_object)

nrow(E) # ! make sure this value is in range from 10,000 to 15,000
```

```
# [1] 12000
```

```
E[1:3, 1:3]
```

```
#        MF.64pLYVEpIIn.Ao.1 MF.64pLYVEpIIn.Ao.2 MF.64pLYVEpIIn.Ao.3
# Actb              14.77450            14.97466            14.80920
# Cst3              12.73106            12.80513            12.62843
```

```
# Eef1a1             12.14864            12.38202            12.33756
```

Genes in your dataset may be named as Symbol, Entrez, Ensembl or RefSeq IDs. One of these names should be specified as value of `gene.id.type` parameter in `prepareData()`.

```
E.prep <- prepareData(E = E,
                      gene.id.type = "Symbol",
                      use.PCA = FALSE,
                      network.annotation = network.annotation)

E.prep[1:3, 1:3]
```

```
#       MF.64pLYVEpIIn.Ao.1 MF.64pLYVEpIIn.Ao.2 MF.64pLYVEpIIn.Ao.3
# 11461           0.7947992           1.0375496           0.8368845
# 13010           0.3038730           0.3471841           0.2438681
# 13627           0.4187557           0.6674637           0.6200901
```

Preparing network

The `prepareNetwork()` function defines the structure of the final metabolic modules.

```
network.prep <- prepareNetwork(E = E.prep,
                               network = network,
                               topology = "metabolites",
                               met.to.filter = met.to.filter,
                               network.annotation = network.annotation,
                               gene2reaction.extra = gene2reaction.extra) # for combined
                                ↪  network
```

```
# INFO [2026-07-20 10:25:20] Global metabolite network contains 6583 edges.
```

```
# INFO [2026-07-20 10:25:20] Largest connected component of this global network contains
 ↪  1430 nodes and 5121 edges.
```

Preclustering

The `preClustering()` function defines initial patterns using k-medoids clustering on gene expression matrix. It is strongly recommended to do initial clustering with no less than 32 clusters (`initial.number.of.clusters = 32`).

You can visualize the initial heatmap as shown below.

```
cur.centers <- preClustering(E.prep = E.prep,
                             network.prep = network.prep,
                             initial.number.of.clusters = 32,
                             network.annotation = network.annotation)
```

```
# INFO [2026-07-20 10:25:20] 1160 metabolic genes from the analysed dataset mapped to
 ↪  this component.
```

```
cur.centers[1:3, 1:3]
```

```
#   MF.64pLYVEpIIn.Ao.1 MF.64pLYVEpIIn.Ao.2 MF.64pLYVEpIIn.Ao.3
# 1         -0.09284514         -0.12763246          0.02188235
# 2          0.03432400          0.02773624          0.13477218
# 3          0.78988889          0.80944803          0.87514519
```

```
pheatmap::pheatmap(
      GAMclust:::normalize.rows(cur.centers),
```

```
      cluster_rows=F, cluster_cols=F,
      show_rownames=F, show_colnames=T)
```

GAM-clustering analysis

Now you have everything prepared for the GAM-clustering analysis.

Initial patterns will be now refined in an iterative process. The output of `gamClustering()` function presents a set of specific subnetworks (also called metabolic modules) that reflect metabolic variability within a given transcriptional dataset.

Note, that it may take a long time to derive metabolic modules by `gamClustering()` function (tens of minutes).

There is a set of parameters which determine the size and number of your final modules. We recommend you to start with the default settings, however you can adjust them based on your own preferences:

1. If you consider final modules to be too small or too big and it complicates interpretation for you, you can either increase or reduce by 10 units the `max.module.size` parameter.
2. If among final modules you consider presence of any modules with too similar patterns, you can reduce by 0.1 units the `cor.threshold` parameter.
3. If among final modules you consider presence of any uninformative modules, you can reduce by 10 times the `p.adj.val.threshold` parameter.

```
results <- gamClustering(E.prep = E.prep,
                         network.prep = network.prep,
                         cur.centers = cur.centers,

                         start.base = 0.5,
                         base.dec = 0.1,
                         max.module.size = 50,

                         cor.threshold = 0.7,
                         p.adj.val.threshold = 1e-5,

                         batch.solver = seq_batch_solver(solver),
                         work.dir = work.dir,

                         show.intermediate.clustering = F,
                         verbose = T,
                         collect.stats = T)
```

Visualizing and exploring the GAM-clustering results

Each metabolic module is a connected piece of metabolic network whose genes expression is correlated across all dataset.

The following functions will help you to visualize and explore the obtained results.

Get graphs of modules:

```
getGraphs(modules = results$modules,
          network.annotation = network.annotation,
          metabolites.annotation = metabolites.annotation,
          seed.for.layout = 42,
```

```
          work.dir = work.dir)
```

```
# Graphs for module 1 are built
# Graphs for module 2 are built
# Graphs for module 3 are built
# Graphs for module 4 are built
# Graphs for module 5 are built
# Graphs for module 6 are built
# Graphs for module 7 are built
# Graphs for module 8 are built
# Graphs for module 9 are built
```

Example of the graph of the first module:

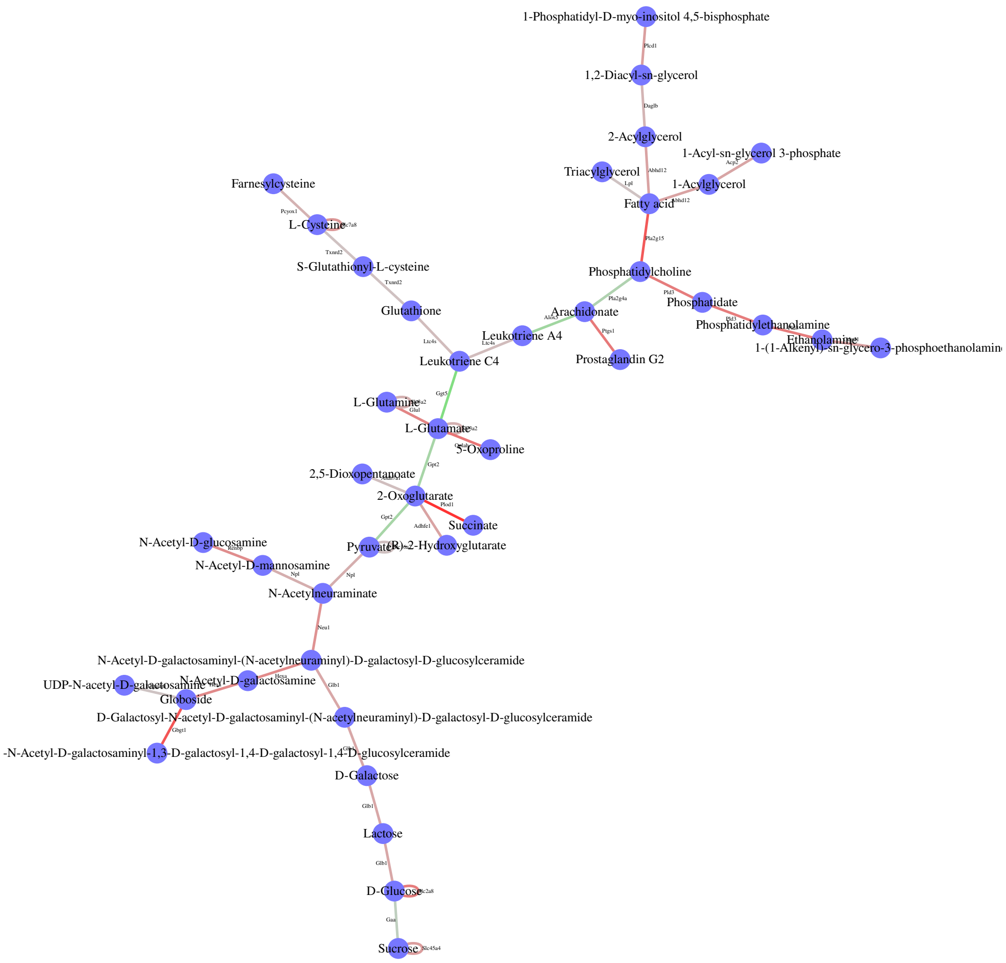

Get gene tables:

The table contains gene list. Each gene has two descriptive values: i) gene's correlation value with the modules pattern and ii) gene's score. High score means that this gene's expression is similar to the module's pattern and not similar to other modules' patterns.

```r
m.gene.list <- getGeneTables(modules = results$modules,
                             nets = results$nets,
                             patterns = results$patterns.pos,
                             gene.exprs = E.prep,
                             network.annotation = network.annotation,
                             work.dir = work.dir)
```

```
# Gene tables for module 1 are produced

# Gene tables for module 2 are produced

# Gene tables for module 3 are produced

# Gene tables for module 4 are produced

# Gene tables for module 5 are produced

# Gene tables for module 6 are produced

# Gene tables for module 7 are produced

# Gene tables for module 8 are produced

# Gene tables for module 9 are produced
```

Example of the gene table of the first module:

```r
head(GAMclust:::read.tsv(file.path(work.dir, "m.1.genes.tsv"))) |>
  kableExtra::kable() |>
  kableExtra::kable_styling()
```

symbol	Entrez	score	cor
Plod1	18822	2.211137	0.9426139
Gbgt1	227671	1.545965	0.9089996
Pla2g15	192654	1.501044	0.9061216
Oplah	75475	1.120957	0.8778247
Ptgs1	19224	1.081628	0.8744483
Slc2a8	56017	1.042379	0.8709858

Get plots of patterns and individual genes expression:

Heatmap for patterns of all modules:

```r
patterns <- results$patterns.pos

pheatmap::pheatmap(
  GAMclust:::normalize.rows(patterns),
  cluster_rows=F, cluster_cols=F,
  show_rownames=T, show_colnames=T)
```

Get plots of individual genes expression

```r
for (i in seq_along(m.gene.list)) {

  heatmap <- E[m.gene.list[[i]], , drop=F]

  pheatmap::pheatmap(
    GAMclust:::normalize.rows(heatmap),
```

```
    cluster_rows=F, cluster_cols=F,
    file=sprintf("%s/m.%s.genes.png", work.dir, i),
    width=10, height=5,
    show_rownames=T, show_colnames=T)
}
```

Example of the heatmap of the first module:

```
pheatmap::pheatmap(
  GAMclust:::normalize.rows(E[m.gene.list[[1]], , drop=F]),
  show_rownames=T, show_colnames=T)
```

Get annotation tables and annotation heatmap for all modules:

Functional annotation of obtained modules is done based on KEGG and Reactome canonical metabolic pathways.

```
getAnnotationTables(network.annotation = network.annotation,
                    nets = results$nets,
                    work.dir = work.dir)
```

```
# Pathway annotation for module 1 is produced

# Pathway annotation for module 2 is produced

# Pathway annotation for module 3 is produced

# Pathway annotation for module 4 is produced

# Pathway annotation for module 5 is produced

# Pathway annotation for module 6 is produced

# Pathway annotation for module 7 is produced

# Pathway annotation for module 8 is produced

# Pathway annotation for module 9 is produced
```

Example of the annotation table of the first module:

```
head(GAMclust:::read.tsv(file.path(work.dir, "m.1.pathways.tsv"))) |>
  kableExtra::kable() |>
  kableExtra::kable_styling(font_size = 8) |>
  kableExtra::column_spec(1, width = "1.6in") |>
  kableExtra::column_spec(2:6, width = "0.5in") |>
  kableExtra::column_spec(7, width = "1.2in")
```

pathway	pval	padj	foldEnrichment	overlap	size	overlapGenes
mmu00590: Arachidonic acid metabolism	0.0000713	0.0170328	9.250000	5	10	Alox5 Ltc4s Pla2g4a Ptgs1 Ggt5
mmu00511: Other glycan degradation	0.0025885	0.2262868	9.250000	3	6	Glb1 Hexa Neu1
mmu00603: Glycosphingolipid biosynthesis - globo and isoglobo series	0.0043623	0.2606492	7.928571	3	7	Hexa Gbgt1 B3galnt1
mmu00565: Ether lipid metabolism	0.0097110	0.4641838	6.166667	3	9	Pla2g4a Pld3 Gdpd1
mmu00220: Arginine biosynthesis	0.0502266	1.0000000	5.285714	2	7	Gpt2 Glul

Annotation heatmap for all modules:

```r
getAnnotationHeatmap(work.dir = work.dir)
```

```
# Processing module 1

# Module size: 34

# Processing module 2

# Module size: 19

# Processing module 3

# Module size: 10

# Processing module 4

# Module size: 12

# Processing module 5

# Module size: 7

# Processing module 6

# Module size: 5

# Processing module 7

# Module size: 5

# Processing module 8

# Module size: 5

# Processing module 9

# Module size: 6
```

Compare modules obtained in different runs:

You may also compare two results of running GAM-clustering on the same dataset (e.g. runs with different parameters) or compare two results of running GAM-clustering on different datasets (then set `same.data=FALSE`).

```r
modulesSimilarity(dir1 = work.dir,
                  dir2 = "old_dir",
                  name1 = "new",
                  name2 = "old",
                  same.data = TRUE,
                  use.genes.with.pos.score = TRUE,
                  work.dir = work.dir,
                  file.name = "comparison.png")
```

Session info

Elapsed time: 36.5 mins.

Peak R memory usage: 1.1 GB

```r
sessionInfo()
```

```
# R version 4.5.3 (2026-03-11)
# Platform: x86_64-pc-linux-gnu
# Running under: Debian GNU/Linux 13 (trixie)
#
# Matrix products: default
# BLAS:   /usr/lib/x86_64-linux-gnu/openblas-pthread/libblas.so.3
# LAPACK: /usr/lib/x86_64-linux-gnu/openblas-pthread/libopenblasp-r0.3.29.so;  LAPACK
 ↪  version 3.12.0
#
# locale:
#  [1] LC_CTYPE=C.utf8       LC_NUMERIC=C          LC_TIME=C.utf8
 ↪  LC_COLLATE=C.utf8     LC_MONETARY=C.utf8
#  [6] LC_MESSAGES=C.utf8    LC_PAPER=C.utf8       LC_NAME=C             LC_ADDRESS=C
 ↪  LC_TELEPHONE=C
# [11] LC_MEASUREMENT=C.utf8 LC_IDENTIFICATION=C
#
# time zone: America/Chicago
# tzcode source: system (glibc)
#
# attached base packages:
# [1] stats     graphics  grDevices utils     datasets  methods   base
#
# other attached packages:
#  [1] futile.logger_1.4.9 fgsea_1.39.4        mwcsr_0.1.12        gatom_1.8.4
 ↪  GAMclust_0.1.0
#  [6] data.table_1.18.4   rmarkdown_2.31      rlang_1.3.0         devtools_2.5.0
 ↪  usethis_3.2.1
#
# loaded via a namespace (and not attached):
#  [1] tidyselect_1.2.1     viridisLite_0.4.3    dplyr_1.2.1          farver_2.1.2
 ↪  blob_1.3.0
#  [6] Biostrings_2.78.0    S7_0.2.2             fastmap_1.2.0        GGally_2.4.0
 ↪  XML_3.99-0.23
# [11] digest_0.6.39        lifecycle_1.0.5      rsvg_2.7.0           ellipsis_0.3.2
 ↪  KEGGREST_1.53.5
# [16] RSQLite_3.53.3       magrittr_2.0.5       compiler_4.5.3       tools_4.5.3
 ↪  igraph_2.3.3
# [21] yaml_2.3.12          knitr_1.51           lambda.r_1.2.4       htmlwidgets_1.6.4
 ↪  bit_4.6.0
# [26] pkgbuild_1.4.8       xml2_1.5.2           plyr_1.8.9           RColorBrewer_1.1-3
 ↪  pkgload_1.5.2
# [31] rsconnect_1.7.0      BiocParallel_1.44.0  purrr_1.2.2          BiocGenerics_0.56.0
 ↪  shinyCyJS_1.0.0
# [36] grid_4.5.3           stats4_4.5.3         ggplot2_4.0.3        scales_1.4.0
 ↪  tinytex_0.60
# [41] cli_3.6.6            crayon_1.5.3         generics_0.1.4       otel_0.2.0
 ↪  rstudioapi_0.17.1
# [46] httr_1.4.8           sessioninfo_1.2.3    DBI_1.3.0            cachem_1.1.0
 ↪  stringr_1.6.0
# [51] parallel_4.5.3       AnnotationDbi_1.72.0 formatR_1.14         XVector_0.50.0
 ↪  matrixStats_1.5.0
# [56] vctrs_0.7.3          Matrix_1.7-4         IRanges_2.44.0       S4Vectors_0.48.1
 ↪  bit64_4.8.2
# [61] BioNet_1.70.0        Rgraphviz_2.54.0     systemfonts_1.3.1    tidyr_1.3.2
 ↪  glue_1.8.1
```

```
# [66] ggstats_0.13.0       codetools_0.2-20     cowplot_1.2.0        stringi_1.8.7
↪  gtable_0.3.6
# [71] tibble_3.3.1         pillar_1.11.1        htmltools_0.5.9      Seqinfo_1.0.0
↪  graph_1.88.1
# [76] R6_2.6.1             textshaping_1.0.4    kableExtra_1.4.0     lattice_0.22-7
↪  evaluate_1.0.5
# [81] Biobase_2.70.0       futile.options_1.0.1 png_0.1-9            pheatmap_1.0.13
↪  memoise_2.0.1
# [86] fastmatch_1.1-8      Rcpp_1.1.2           svglite_2.2.2        xfun_0.60
↪  fs_2.1.0
# [91] pkgconfig_2.0.3
```
