# Supplementary material for: GAMclust: identification of regulated metabolic modules in bulk, single cell and spatial gene expression data
Source: Bioinformatics. 2026 Aug 22;42(9):btag639. doi: 10.1093/bioinformatics/btag639 (PMC13544963; doi:10.1093/bioinformatics/btag639)
Supplement: Supplementary file 1 [file btag639_supplementary_data.zip › S4_GAMclust_tutorial_SC.pdf]

GAM-clustering reanalysis of Tabula Muris Senis single cell RNA-seq data

Introduction

This vignette walks over application of GAM-clustering method for reanalysis of metabolic regulation in Tabula Muris Senis single cell RNA-seq data (mononuclear phagocyte subset), originally described in Gainullina et al.

The vignette requires about an hour of computational time and 16GB of memory for completion.

Installation and libraries

Install GAMclust package:

```
devtools::install_github("alserglab/GAMclust")
```

```
library(GAMclust)
library(gatom)
library(mwcsr)
library(fgsea)
library(data.table)
library(Seurat)
library(BiocFileCache)
library(futile.logger)


set.seed(42)
```

```
startTime <- Sys.time()
```

Preparing working environment

First, load and initialize all objects required for GAM-clustering analysis:

(1) load metabolic network and its metabolites annotation. We provide two networks: KEGG and combined network that includes KEGG, Rhea and transport reactions:

(1.1.) load KEGG metabolic network `network.kegg.rds` and its metabolites annotation `met.kegg.db.rds`

```
# KEGG network:
network <- readRDS(url("http://artyomovlab.wustl.edu/publications/supp_materials/GATOM/ne
↪  twork.kegg.rds"))
metabolites.annotation <- readRDS(url("http://artyomovlab.wustl.edu/publications/supp_mat
↪  erials/GATOM/met.kegg.db.rds"))
```

(1.2.) or load combined metabolic network `network.combined.rds`, its metabolites annotation `met.combined.db.rds` and species-specific list of genes that either come from proteome or are not linked to a specific enzyme `gene2reaction.combined.mmu.eg.tsv` for mouse and `gene2reaction.combined.hsa.eg.tsv` for human data;

```
# combined network (KEGG+Rhea+transport reactions):
network <- readRDS(url("http://artyomovlab.wustl.edu/publications/supp_materials/GATOM/ne
↪  twork.combined.rds"))
metabolites.annotation <- readRDS(url("http://artyomovlab.wustl.edu/publications/supp_mat
↪  erials/GATOM/met.combined.db.rds"))
gene2reaction.extra <- data.table::fread("http://artyomovlab.wustl.edu/publications/supp_
↪  materials/GATOM/gene2reaction.combined.mmu.eg.tsv", colClasses="character")
```

(2) load species-specific network annotation: `org.Hs.eg.gatom.anno` for human data or `org.Mm.eg.gatom.anno` for mouse data;

```
network.annotation <- readRDS(url("http://artyomovlab.wustl.edu/publications/supp_materia
↪  ls/GATOM/org.Mm.eg.gatom.anno.rds"))
```

(3) load provided list of metabolites that should not be considered during the analysis as connections between reactions (e.g., CO2, HCO3-, etc);

```
met.to.filter <- data.table::fread(system.file("mets2mask.lst", package="GAMclust"))$ID
```

(4) initialize MWCS solver:

(4.1.) we recommend to use here either heuristic relax-and-cut solver `rnc_solver` from `mwcsr` package,

```
solver <- mwcsr::rnc_solver()
```

(4.2.) either proprietary CPLEX solver (free for academy);

```
cplex.dir <- "/opt/ibm/ILOG/CPLEX_Studio1271"
```

```
solver <- mwcsr::virgo_solver(cplex_dir = cplex.dir)
```

(5) set working directory where the results will be saved to.

```
work.dir <- "results-sc"
dir.create(work.dir, showWarnings = F, recursive = T)
```

Preparing objects for the analysis

Preparing data

GAMclust works with bulk, single cell and spatial RNA-seq data.

This vignette shows how to process single cell RNA-seq data on the example of Tabula Muris Senis data reanalysis.

For single cell data, take 6,000-12,000 genes for the GAM-clustering analysis. To do this while preprocessing data with Seurat pipeline, set `variable.features.n = 12000` in `SCTransform()` function. In case of preprocessing multi-sample data, set `nfeatures=12000` in `SelectIntegrationFeatures()`).

Let's load already preprocessed data.

```
bfc <- BiocFileCache("./cache", ask = FALSE)
path <- bfcrpath(
  bfc,
  "http://artyomovlab.wustl.edu/publications/supp_materials/GAMclust/tms_sct12k.rds"
)
```

```
seurat_object <- readRDS(path)
```

```r
E <- as.matrix(Seurat::GetAssayData(object = seurat_object,
                                    assay = "SCT",
                                    layer = "scale.data"))

nrow(E) # ! make sure this value is in range from 6,000 to 12,000
```

```
# [1] 12000
```

```r
E[1:3, 1:3]
```

```
#         AACCATGAGATCCCAT-1-0-0-0 AACCATGTCAGAGGTG-1-0-0-0 AAGGTTCTCCTAGAAC-1-0-0-0
# Sox17                 -0.1865187               -0.2478802               -0.2390427
# Mrpl15                -0.4440852                0.3323707               -0.5901840
# Lypla1                -0.4182570               -0.5818529               -0.5602515
```

Genes in your dataset may be named as Symbol, Entrez, Ensembl or RefSeq IDs. One of these names should be specified as value of `gene.id.type` parameter in `prepareData()`.

If you analyse singe cell or spatial RNA-seq data, set `use.PCA=TRUE` in `prepareData()`.

```r
E.prep <- prepareData(E = E,
                      gene.id.type = "Symbol",
                      use.PCA = TRUE,
                      use.PCA.n = 50,
                      network.annotation = network.annotation)

E.prep[1:3, 1:3]
```

```
#               PC1      PC2        PC3
# 16176  -0.0419378 2.222356 -0.7472891
# 278180 -1.2188519 2.806405  1.6716989
# 56744  -1.5140244 3.368009  1.5328317
```

Preparing network

The `prepareNetwork()` function defines the structure of the final metabolic modules.

```r
network.prep <- prepareNetwork(E = E.prep,
                               network = network,
                               topology = "metabolites",
                               met.to.filter = met.to.filter,
                               network.annotation = network.annotation,
                               gene2reaction.extra = gene2reaction.extra) # for combined
    network
```

```
# INFO [2026-07-20 11:48:08] Global metabolite network contains 6754 edges.
```

```
# INFO [2026-07-20 11:48:08] Largest connected component of this global network contains
    1397 nodes and 5139 edges.
```

Preclustering

The `preClustering()` function defines initial patterns using k-medoids clustering on gene expression matrix. It is strongly recommended to do initial clustering with no less than 32 clusters (`initial.number.of.clusters = 32`).

You can visualize the initial heatmap as shown below.

```r
cur.centers <- preClustering(E.prep = E.prep,
                             network.prep = network.prep,
                             initial.number.of.clusters = 32,
                             network.annotation = network.annotation)
```

```
# INFO [2026-07-20 11:48:08] 1123 metabolic genes from the analysed dataset mapped to
↪  this component.
```

```r
cur.centers[1:3, 1:3]
```

```
#         PC1       PC2         PC3
# 1 -4.140217 -3.588960  0.47784732
# 2 -3.374871  1.319177 -1.89015590
# 3 -4.281918 -0.421322 -0.08240903
```

```r
pheatmap::pheatmap(
      GAMclust:::normalize.rows(cur.centers),
      cluster_rows=F, cluster_cols=F,
      show_rownames=F, show_colnames=T)
```

GAM-clustering analysis

Now you have everything prepared for the GAM-clustering analysis.

Initial patterns will be now refined in an iterative process. The output of `gamClustering()` function presents a set of specific subnetworks (also called metabolic modules) that reflect metabolic variability within a given transcriptional dataset.

Note, that it may take a long time to derive metabolic modules by `gamClustering()` function (tens of minutes).

There is a set of parameters which determine the size and number of your final modules. We recommend you to start with the default settings, however you can adjust them based on your own preferences:

1. If you consider final modules to bee too small or too big and it complicates interpretation for you, you can either increase or reduce by 10 units the `max.module.size` parameter.
2. If among final modules you consider presence of any modules with too similar patterns, you can reduce by 0.1 units the `cor.threshold` parameter.
3. If among final modules you consider presence of any uninformative modules, you can reduce by 10 times the `p.adj.val.threshold` parameter.

```r
results <- gamClustering(E.prep = E.prep,
                         network.prep = network.prep,
                         cur.centers = cur.centers,

                         start.base = 0.5,
                         base.dec = 0.05,
                         max.module.size = 50,

                         cor.threshold = 0.8,
                         p.adj.val.threshold = 1e-5,

                         batch.solver = seq_batch_solver(solver),
                         work.dir = work.dir,

                         show.intermediate.clustering = FALSE,
```

```
                                 verbose = FALSE,
                                 collect.stats = TRUE)
```

Visualizing and exploring the GAM-clustering results

Each metabolic module is a connected piece of metabolic network whose genes expression is correlated across all dataset.

The following functions will help you to visualize and explore the obtained results.

Get graphs of modules:

```
getGraphs(modules = results$modules,
          network.annotation = network.annotation,
          metabolites.annotation = metabolites.annotation,
          seed.for.layout = 42,
          work.dir = work.dir)
```

```
# Graphs for module 1 are built

# Graphs for module 2 are built

# Graphs for module 3 are built

# Graphs for module 4 are built

# Graphs for module 5 are built

# Graphs for module 6 are built

# Graphs for module 7 are built

# Graphs for module 8 are built
```

Example of the graph of the third module:

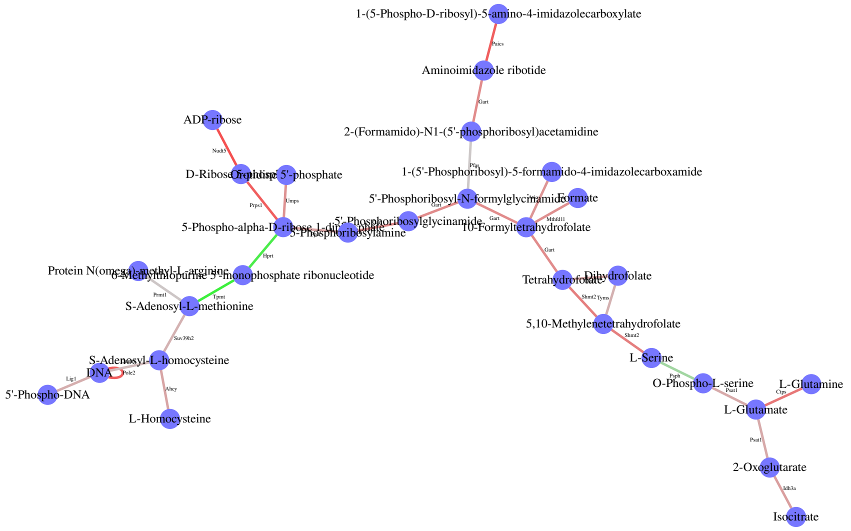

Get gene tables:

The table contains gene list. Each gene has two descriptive values: i) gene's correlation value with the modules pattern and ii) gene's score. High score means that this gene's expression is similar to the module's

pattern and not similar to other modules' patterns.

```
m.gene.list <- getGeneTables(modules = results$modules,
                             nets = results$nets,
                             patterns = results$patterns.pos,
                             gene.exprs = E.prep,
                             network.annotation = network.annotation,
                             work.dir = work.dir)
```

```
# Gene tables for module 1 are produced

# Gene tables for module 2 are produced

# Gene tables for module 3 are produced

# Gene tables for module 4 are produced

# Gene tables for module 5 are produced

# Gene tables for module 6 are produced

# Gene tables for module 7 are produced

# Gene tables for module 8 are produced
```

Example of the gene table of the third module:

```
head(GAMclust:::read.tsv(file.path(work.dir, "m.3.genes.tsv"))) |>
  kableExtra::kable() |>
  kableExtra::kable_styling()
```

symbol	Entrez	score	cor
Nudt5	53893	1.6042778	0.9406440
Pole2	18974	1.4296715	0.9383090
Prps1	19139	1.4135713	0.9341166
Paics	67054	1.4146098	0.9325548
Ctps	51797	1.0938273	0.9229224
Idh3a	67834	0.5849869	0.9184430

Get plots of patterns:

```
for(i in 1:length(m.gene.list)){

  print(fgsea::plotCoregulationProfileReduction(m.gene.list[[i]],
                                                seurat_object,
                                                title = paste0("module ", i),
                                                raster = TRUE,
                                                reduction = "pumap"))
}
```

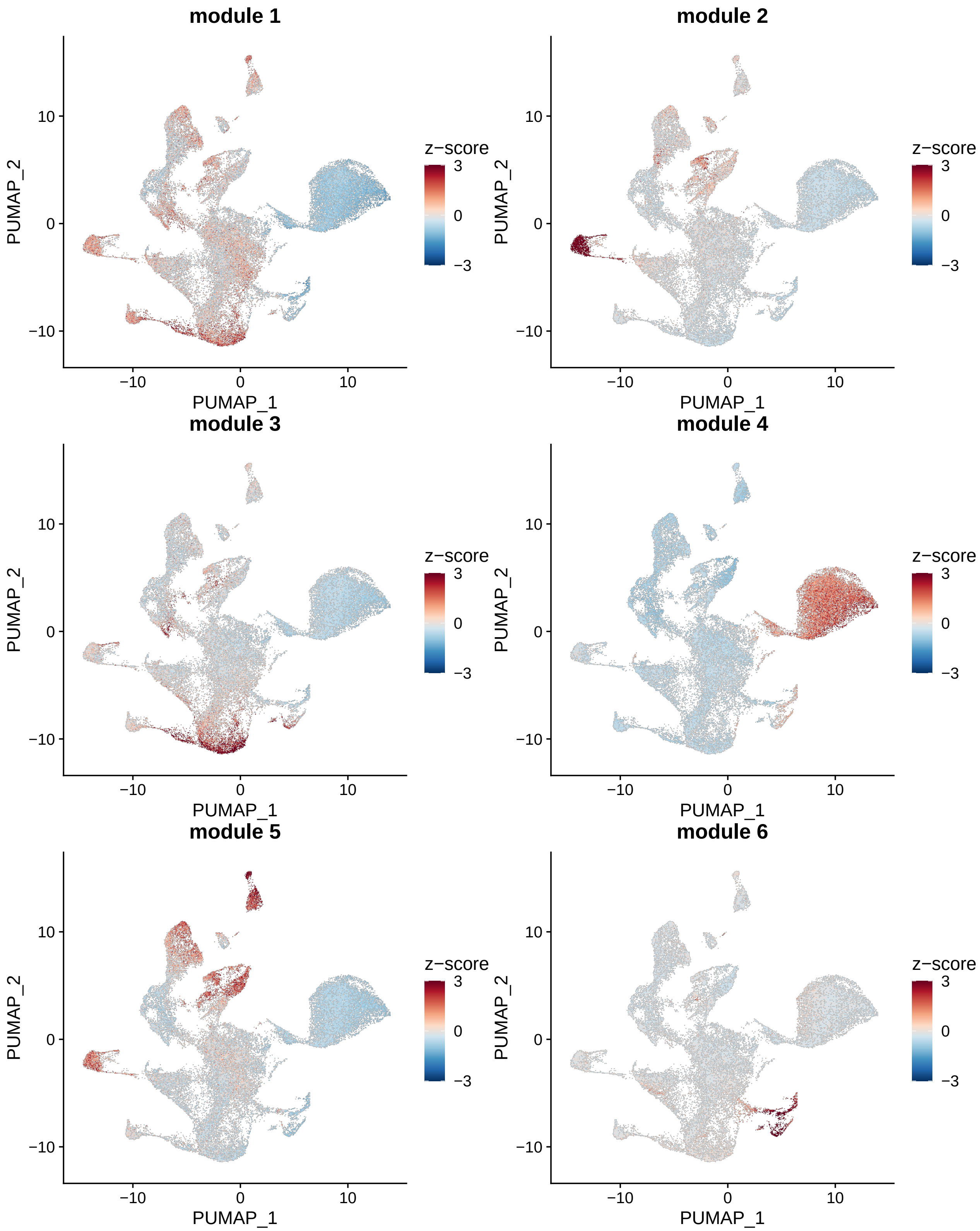
module 1
module 2
module 3
module 4
module 5
module 6
PUMAP_2
PUMAP_1
z−score
3
0
−3
10
0
−10
−10
0
10

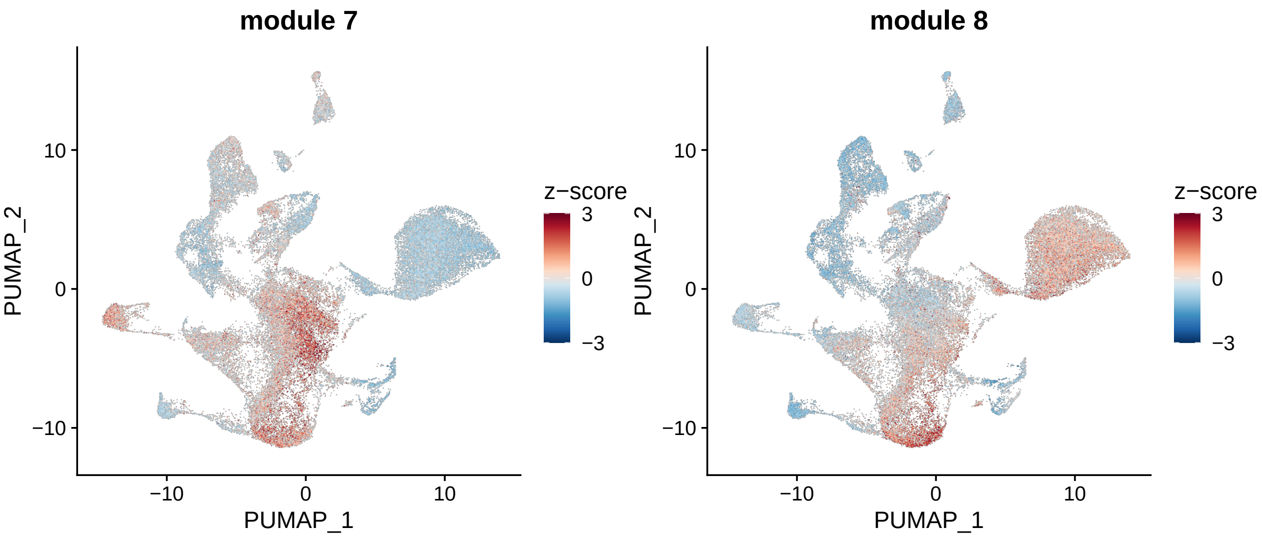

Get plots of individual genes expression (example for the third module):

```r
Seurat::DefaultAssay(seurat_object) <- "SCT"

i <- 3

Seurat::FeaturePlot(seurat_object,
                    reduction = "pumap",
                    order = T,
                    features = m.gene.list[[i]],
                    ncol = 6,
                    raster = TRUE,
                    combine = TRUE)
```

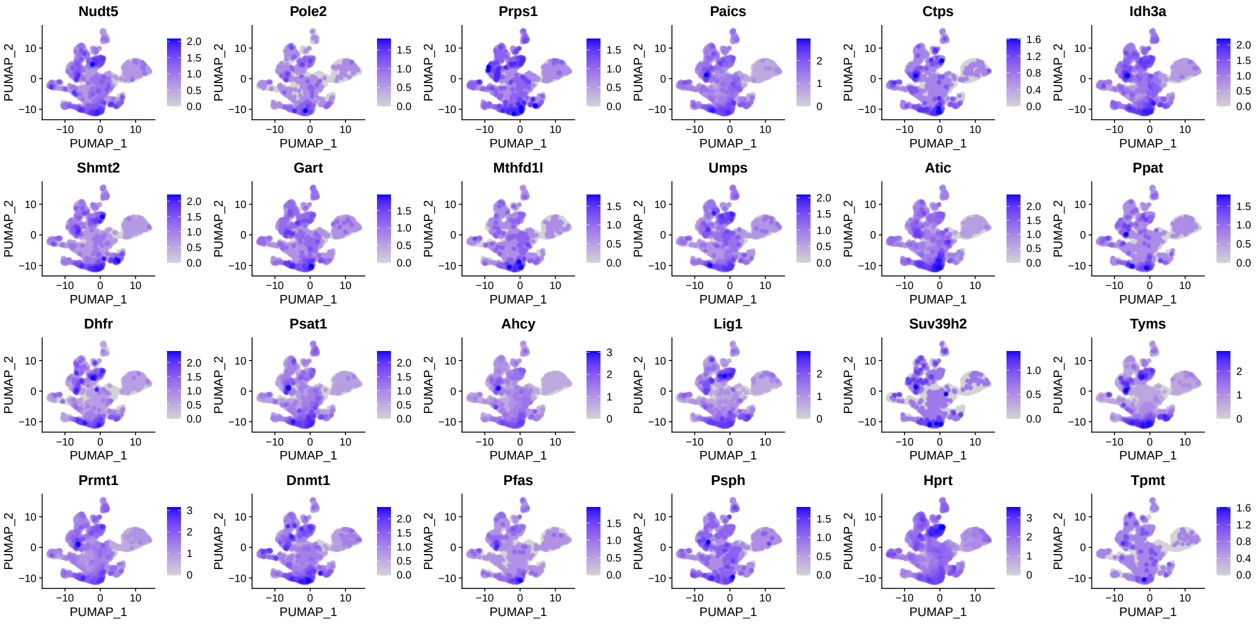

Get tables and plots with annotation of modules:

Functional annotation of obtained modules is done based on KEGG and Reactome canonical metabolic pathways.

```
getAnnotationTables(network.annotation = network.annotation,
                    nets = results$nets,
                    work.dir = work.dir)
```

```
# Pathway annotation for module 1 is produced

# Pathway annotation for module 2 is produced

# Pathway annotation for module 3 is produced

# Pathway annotation for module 4 is produced

# Pathway annotation for module 5 is produced

# Pathway annotation for module 6 is produced

# Pathway annotation for module 7 is produced

# Pathway annotation for module 8 is produced
```

Example of the annotation table of the third module:

```
head(GAMclust:::read.tsv(file.path(work.dir, "m.3.pathways.tsv"))) |>
  kableExtra::kable() |>
  kableExtra::kable_styling(font_size = 8) |>
  kableExtra::column_spec(1, width = "1.6in") |>
  kableExtra::column_spec(2:6, width = "0.5in") |>
  kableExtra::column_spec(7, width = "1.2in")
```

pathway	pval	padj	foldEnrichment	overlap	size	overlapGenes
mmu00230: Purine metabolism	0.0000005	0.0000572	8.956522	8	23	Atic Gart Hprt Prps1 Ppat Pfas Nudt5 Paics
mmu00670: One carbon pool by folate	0.0000007	0.0000572	14.045454	6	11	Shmt2 Atic Dhfr Gart Tyms Mthfd1l
mmu_M00048: De novo purine biosynthesis, PRPP + glutamine => IMP	0.0000003	0.0000572	21.458333	5	6	Atic Gart Ppat Pfas Paics
mmu_M00035: Methionine degradation	0.0014477	0.0857736	25.750000	2	2	Dnmt1 Ahcy
mmu_M00020: Serine biosynthesis, glycerate-3P => serine	0.0042396	0.1674628	17.166667	2	3	Psph Psat1

Annotation heatmap for all modules:

```
getAnnotationHeatmap(work.dir = work.dir)
```

```
# Processing module 1

# Module size: 40

# Processing module 2

# Module size: 21

# Processing module 3

# Module size: 24
```

```
# Processing module 4

# Module size: 21

# Processing module 5

# Module size: 14

# Processing module 6

# Module size: 18

# Processing module 7

# Module size: 8

# Processing module 8

# Module size: 4
```

Compare modules obtained in different runs:

You may also compare two results of running GAM-clustering on the same dataset (e.g. runs with different parameters) or compare two results of running GAM-clustering on different datasets (then set `same.data=FALSE`).

```
modulesSimilarity(dir1 = work.dir,
                  dir2 = "old.dir",
                  name1 = "new",
                  name2 = "old",
                  same.data = TRUE,
                  use.genes.with.pos.score = TRUE,
                  work.dir = work.dir,
                  file.name = "comparison.png")
```

Session info

Elapsed time: 47.1 mins.

Peak R memory usage: 14.7 GB

```
sessionInfo()
```

```
# R version 4.5.3 (2026-03-11)
# Platform: x86_64-pc-linux-gnu
# Running under: Debian GNU/Linux 13 (trixie)
#
# Matrix products: default
# BLAS:   /usr/lib/x86_64-linux-gnu/openblas-pthread/libblas.so.3
# LAPACK: /usr/lib/x86_64-linux-gnu/openblas-pthread/libopenblasp-r0.3.29.so;  LAPACK
 ↪  version 3.12.0
#
# locale:
#  [1] LC_CTYPE=C.utf8       LC_NUMERIC=C           LC_TIME=C.utf8
 ↪  LC_COLLATE=C.utf8     LC_MONETARY=C.utf8
#  [6] LC_MESSAGES=C.utf8    LC_PAPER=C.utf8       LC_NAME=C              LC_ADDRESS=C
 ↪  LC_TELEPHONE=C
# [11] LC_MEASUREMENT=C.utf8 LC_IDENTIFICATION=C
#
# time zone: America/Chicago
# tzcode source: system (glibc)
```

```
#
# attached base packages:
# [1] stats     graphics  grDevices utils     datasets  methods   base
#
# other attached packages:
#  [1] BiocFileCache_3.0.0 dbplyr_2.5.1        future_1.70.0       Seurat_5.5.0
 ↪  SeuratObject_5.4.0
#  [6] sp_2.2-1            futile.logger_1.4.9 fgsea_1.39.4        mwcsr_0.1.12
 ↪  gatom_1.8.4
# [11] GAMclust_0.1.0      data.table_1.18.4   rmarkdown_2.31      rlang_1.3.0
 ↪  devtools_2.5.0
# [16] usethis_3.2.1
#
# loaded via a namespace (and not attached):
#   [1] RcppAnnoy_0.0.23           splines_4.5.3              later_1.4.8
 ↪  filelock_1.0.3
#   [5] tibble_3.3.1               polyclip_1.10-7            graph_1.88.1
 ↪  XML_3.99-0.23
#   [9] fastDummies_1.7.6          httr2_1.2.2                lifecycle_1.0.5
 ↪  globals_0.19.1
#  [13] lattice_0.22-7             MASS_7.3-65                magrittr_2.0.5
 ↪  plotly_4.12.0
#  [17] yaml_2.3.12                httpuv_1.6.17              otel_0.2.0
 ↪  glmGamPoi_1.22.0
#  [21] sctransform_0.4.3          spam_2.11-3                sessioninfo_1.2.3
 ↪  pkgbuild_1.4.8
#  [25] spatstat.sparse_3.1-0      reticulate_1.46.0          cowplot_1.2.0
 ↪  pbapply_1.7-4
#  [29] DBI_1.3.0                  RColorBrewer_1.1-3         abind_1.4-8
 ↪  pkgload_1.5.2
#  [33] GenomicRanges_1.62.1       Rtsne_0.17                 purrr_1.2.2
 ↪  BiocGenerics_0.56.0
#  [37] rappdirs_0.3.4             IRanges_2.44.0             S4Vectors_0.48.1
 ↪  ggrepel_0.9.8
#  [41] irlba_2.3.7                listenv_0.10.1             spatstat.utils_3.2-3
 ↪  pheatmap_1.0.13
#  [45] goftest_1.2-3              RSpectra_0.16-2            spatstat.random_3.4-5
 ↪  fitdistrplus_1.2-6
#  [49] parallelly_1.47.0          DelayedMatrixStats_1.32.0  svglite_2.2.2
 ↪  DelayedArray_0.36.1
#  [53] codetools_0.2-20           xml2_1.5.2                 tidyselect_1.2.1
 ↪  farver_2.1.2
#  [57] shinyCyJS_1.0.0            matrixStats_1.5.0          stats4_4.5.3
 ↪  spatstat.explore_3.8-0
#  [61] Seqinfo_1.0.0              jsonlite_2.0.0             ellipsis_0.3.2
 ↪  progressr_0.19.0
#  [65] ggridges_0.5.7             survival_3.8-3             systemfonts_1.3.1
 ↪  tools_4.5.3
#  [69] ica_1.0-3                  Rcpp_1.1.2                 glue_1.8.1
 ↪  SparseArray_1.10.10
#  [73] gridExtra_2.3              xfun_0.60                  MatrixGenerics_1.22.0
 ↪  dplyr_1.2.1
#  [77] withr_3.0.3                formatR_1.14               fastmap_1.2.0
 ↪  GGally_2.4.0
```

```
#  [81] digest_0.6.39             R6_2.6.1                    mime_0.13
↪  textshaping_1.0.4
#  [85] Cairo_1.7-0               scattermore_1.2             rsvg_2.7.0
↪  tensor_1.5.1
#  [89] spatstat.data_3.1-9       RSQLite_3.53.3              tidyr_1.3.2
↪  generics_0.1.4
#  [93] S4Arrays_1.10.1           httr_1.4.8                  htmlwidgets_1.6.4
↪  ggstats_0.13.0
#  [97] uwot_0.2.4                pkgconfig_2.0.3             gtable_0.3.6
↪  rsconnect_1.7.0
# [101] blob_1.3.0                lmtest_0.9-40               S7_0.2.2
↪  XVector_0.50.0
# [105] htmltools_0.5.9           dotCall64_1.2               BioNet_1.70.0
↪  scales_1.4.0
# [109] kableExtra_1.4.0          Biobase_2.70.0              png_0.1-9
↪  spatstat.univar_3.1-7
# [113] knitr_1.51                lambda.r_1.2.4              rstudioapi_0.17.1
↪  reshape2_1.4.5
# [117] curl_7.1.0                nlme_3.1-168                cachem_1.1.0
↪  zoo_1.8-15
# [121] stringr_1.6.0             KernSmooth_2.23-26          vipor_0.4.7
↪  parallel_4.5.3
# [125] miniUI_0.1.2              AnnotationDbi_1.72.0        ggrastr_1.0.2
↪  pillar_1.11.1
# [129] grid_4.5.3                vctrs_0.7.3                 RANN_2.6.2
↪  promises_1.5.0
# [133] beachmat_2.26.0           xtable_1.8-8                cluster_2.1.8.1
↪  beeswarm_0.4.0
# [137] Rgraphviz_2.54.0          evaluate_1.0.5              tinytex_0.60
↪  cli_3.6.6
# [141] compiler_4.5.3            futile.options_1.0.1        crayon_1.5.3
↪  future.apply_1.20.2
# [145] labeling_0.4.3            ggbeeswarm_0.7.3            plyr_1.8.9
↪  fs_2.1.0
# [149] stringi_1.8.7             deldir_2.0-4                viridisLite_0.4.3
↪  BiocParallel_1.44.0
# [153] Biostrings_2.78.0         lazyeval_0.2.3              spatstat.geom_3.7-3
↪  Matrix_1.7-4
# [157] RcppHNSW_0.6.0            patchwork_1.3.2             sparseMatrixStats_1.22.0
↪  bit64_4.8.2
# [161] ggplot2_4.0.3             KEGGREST_1.53.5             shiny_1.13.0
↪  SummarizedExperiment_1.40.0
# [165] ROCR_1.0-12               igraph_2.3.3                memoise_2.0.1
↪  fastmatch_1.1-8
# [169] bit_4.6.0
```
