# Supplementary material for: GAMclust: identification of regulated metabolic modules in bulk, single cell and spatial gene expression data
Source: Bioinformatics. 2026 Aug 22;42(9):btag639. doi: 10.1093/bioinformatics/btag639 (PMC13544963; doi:10.1093/bioinformatics/btag639)
Supplement: Supplementary file 1 [file btag639_supplementary_data.zip › S5_GAMclust_tutorial_SPAT.pdf]

GAM-clustering analysis of spatial RNA-seq data

Introduction

This vignette walks over application of GAM-clustering method for analysis of metabolic regulation in a glioblastoma sample based on the data from Ravi et al.

The vignette requires about an hour of computational time and 6GB of memory for completion.

Installation and libraries

Install GAMclust package:

```
devtools::install_github("alserglab/GAMclust")
```

```
library(GAMclust)
library(gatom)
library(mwcsr)
library(fgsea)
library(data.table)
library(Seurat)
library(futile.logger)

set.seed(42)
```

```
startTime <- Sys.time()
```

Preparing working environment

First, load and initialize all objects required for GAM-clustering analysis:

(1) load metabolic network and its metabolites annotation. We provide two networks: KEGG and combined network that includes KEGG, Rhea and transport reactions:

(1.1.) load KEGG metabolic network `network.kegg.rds` and its metabolites annotation `met.kegg.db.rds`

```
# KEGG network:
network <- readRDS(url("http://artyomovlab.wustl.edu/publications/supp_materials/GATOM/ne
↪  twork.kegg.rds"))
metabolites.annotation <- readRDS(url("http://artyomovlab.wustl.edu/publications/supp_mat
↪  erials/GATOM/met.kegg.db.rds"))
```

(1.2.) or load combined metabolic network `network.combined.rds`, its metabolites annotation `met.combined.db.rds` and species-specific list of genes that either come from proteome or are not linked to a specific enzyme `gene2reaction.combined.mmu.eg.tsv` for mouse and `gene2reaction.combined.hsa.eg.tsv` for human data;

```
# combined network (KEGG+Rhea+transport reactions):
network <- readRDS(url("http://artyomovlab.wustl.edu/publications/supp_materials/GATOM/ne
↪  twork.combined.rds"))
metabolites.annotation <- readRDS(url("http://artyomovlab.wustl.edu/publications/supp_mat
↪  erials/GATOM/met.combined.db.rds"))
```

```r
gene2reaction.extra <- data.table::fread("http://artyomovlab.wustl.edu/publications/supp_
↪  materials/GATOM/gene2reaction.combined.hsa.eg.tsv", colClasses="character")
```

(2) load species-specific network annotation: `org.Hs.eg.gatom.anno` for human data or `org.Mm.eg.gatom.anno` for mouse data;

```r
network.annotation <- readRDS(url("http://artyomovlab.wustl.edu/publications/supp_materia
↪  ls/GATOM/org.Hs.eg.gatom.anno.rds"))
```

(3) load provided list of metabolites that should not be considered during the analysis as connections between reactions (e.g., CO_2, HCO_3^-, etc);

```r
met.to.filter <- data.table::fread(system.file("mets2mask.lst", package="GAMclust"))$ID
```

(4) initialize MWCS solver:

(4.1.) we recommend to use here either heuristic relax-and-cut solver `rnc_solver` from `mwcsr` package,

```r
solver <- mwcsr::rnc_solver()
```

(4.2.) either proprietary CPLEX solver (free for academy);

```r
cplex.dir <-  "/opt/ibm/ILOG/CPLEX_Studio1271"
```

```r
solver <- mwcsr::virgo_solver(cplex_dir = cplex.dir)
```

(5) set working directory where the results will be saved to.

```r
work.dir <- "results-spat"
dir.create(work.dir, showWarnings = F, recursive = T)
```

(6) TEMPORARY: collecting logs while developing the tool.

```r
stats.dir <- file.path(work.dir, "stats")
dir.create(stats.dir, showWarnings = F, recursive = T)

setup_logger <- function(log.file.path, logger.name = "stats.logger") {
  file.appender <- appender.file(log.file.path)
  console.appender <- appender.console()
  combined.appender <- function(line) {
    file.appender(line)
    console.appender(line)
  }
  flog.appender(combined.appender, name = logger.name)
  flog.threshold(TRACE, name = logger.name)
}

log.file <- file.path(stats.dir, "log.txt")
setup_logger(log.file.path = log.file, logger.name = "stats.logger")
```

Preparing objects for the analysis

Preparing data

GAMclust works with bulk, single cell and spatial RNA-seq data.

This vignette shows how to process spatial RNA-seq data.

Let's load the data and take 10,000 genes for the GAM-clustering analysis.

```
seurat_object <- readRDS(url("http://artyomovlab.wustl.edu/publications/supp_materials/GA
↪  Mclust/275_T_ST_Seurat.rds"))

seurat_object <- Seurat::SCTransform(seurat_object,
                                     assay = "Spatial",
                                     variable.features.n = 10000,
                                     verbose = FALSE)

E <- as.matrix(Seurat::GetAssayData(object = seurat_object,
                                    assay = "SCT",
                                    layer = "scale.data"))

nrow(E) # ! make sure this value is in range from 6,000 to 12,000
```

```
# [1] 10000
```

```
E[1:3, 1:3]
```

```
#        AAACAAGTATCTCCCA-1 AAACACCAATAACTGC-1 AAACAGAGCGACTCCT-1
# NOC2L           1.1571341          0.7969197          3.8411785
# ISG15           2.5017305         -0.7729781          0.6169713
# AGRN           -0.6511085         -0.7490492          0.5783016
```

Genes in your dataset may be named as Symbol, Entrez, Ensembl or RefSeq IDs. One of these names should be specified as value of `gene.id.type` parameter in `prepareData()`.

If you analyse singe cell or spatial RNA-seq data, set `use.PCA=TRUE` in `prepareData()`.

```
E.prep <- prepareData(E = E,
                      gene.id.type = "Symbol",
                      use.PCA = TRUE,
                      use.PCA.n = 50,
                      network.annotation = network.annotation)

E.prep[1:3, 1:3]
```

```
#             PC1        PC2       PC3
# 23480 -5.334666  1.1258589  3.269509
# 6167   2.708519  1.7893544  2.947255
# 60    -4.081374 -0.9354567 -1.662026
```

Preparing network

The `prepareNetwork()` function defines the structure of the final metabolic modules.

```
network.prep <- prepareNetwork(E = E.prep,
                               network = network,
                               topology = "metabolites",
                               met.to.filter = met.to.filter,
                               network.annotation = network.annotation,
                               gene2reaction.extra = gene2reaction.extra) # for combined
                                ↪  network
```

```
# INFO [2026-07-20 11:08:14] Global metabolite network contains 5813 edges.
```

```
# INFO [2026-07-20 11:08:14] Largest connected component of this global network contains
↪  1296 nodes and 4448 edges.
```

Preclustering

The `preClustering()` function defines initial patterns using k-medoids clustering on gene expression matrix. It is strongly recommended to do initial clustering with no less than 32 clusters (`initial.number.of.clusters = 32`).

You can visualize the initial heatmap as shown below.

```
cur.centers <- preClustering(E.prep = E.prep,
                             network.prep = network.prep,
                             initial.number.of.clusters = 32,
                             network.annotation = network.annotation)
```

```
# INFO [2026-07-20 11:08:14] 1001 metabolic genes from the analysed dataset mapped to
↪  this component.
```

```
cur.centers[1:3, 1:3]
```

```
#          PC1        PC2       PC3
# 1  0.6940166 -0.1461196 -0.971703
# 2  3.1863111  1.2636034 -1.358610
# 3 -0.4623664 -3.1075108 -1.622821
```

```
pheatmap::pheatmap(
      GAMclust:::normalize.rows(cur.centers),
      cluster_rows=F, cluster_cols=F,
      show_rownames=F, show_colnames=T)
```

GAM-clustering analysis

Now you have everything prepared for the GAM-clustering analysis.

Initial patterns will be now refined in an iterative process. The output of `gamClustering()` function presents a set of specific subnetworks (also called metabolic modules) that reflect metabolic variability within a given transcriptional dataset.

Note, that it may take a long time to derive metabolic modules by `gamClustering()` function (tens of minutes).

There is a set of parameters which determine the size and number of your final modules. We recommend you to start with the default settings, however you can adjust them based on your own preferences:

1. If you consider final modules to bee too small or too big and it complicates interpretation for you, you can either increase or reduce by 10 units the `max.module.size` parameter.
2. If among final modules you consider presence of any modules with too similar patterns, you can reduce by 0.1 units the `cor.threshold` parameter.
3. If among final modules you consider presence of any uninformative modules, you can reduce by 10 times the `p.adj.val.threshold` parameter.

```
results <- gamClustering(E.prep = E.prep,
                         network.prep = network.prep,
                         cur.centers = cur.centers,

                         start.base = 0.5,
                         base.dec = 0.05,
                         max.module.size = 50,

                         cor.threshold = 0.5,
```

```
                          p.adj.val.threshold = 1e-5,

                          batch.solver = seq_batch_solver(solver),
                          work.dir = work.dir,

                          show.intermediate.clustering = FALSE,
                          verbose = FALSE,
                          collect.stats = TRUE)
```

Visualizing and exploring the GAM-clustering results

Each metabolic module is a connected piece of metabolic network whose genes expression is correlated across all dataset.

The following functions will help you to visualize and explore the obtained results.

Get graphs of modules:

```
getGraphs(modules = results$modules,
          network.annotation = network.annotation,
          metabolites.annotation = metabolites.annotation,
          seed.for.layout = 42,
          work.dir = work.dir)
```

```
# Graphs for module 1 are built
```

```
# Graphs for module 2 are built
```

```
# Graphs for module 3 are built
```

```
# Graphs for module 4 are built
```

```
# Graphs for module 5 are built
```

Example of the graph of the fourth module:

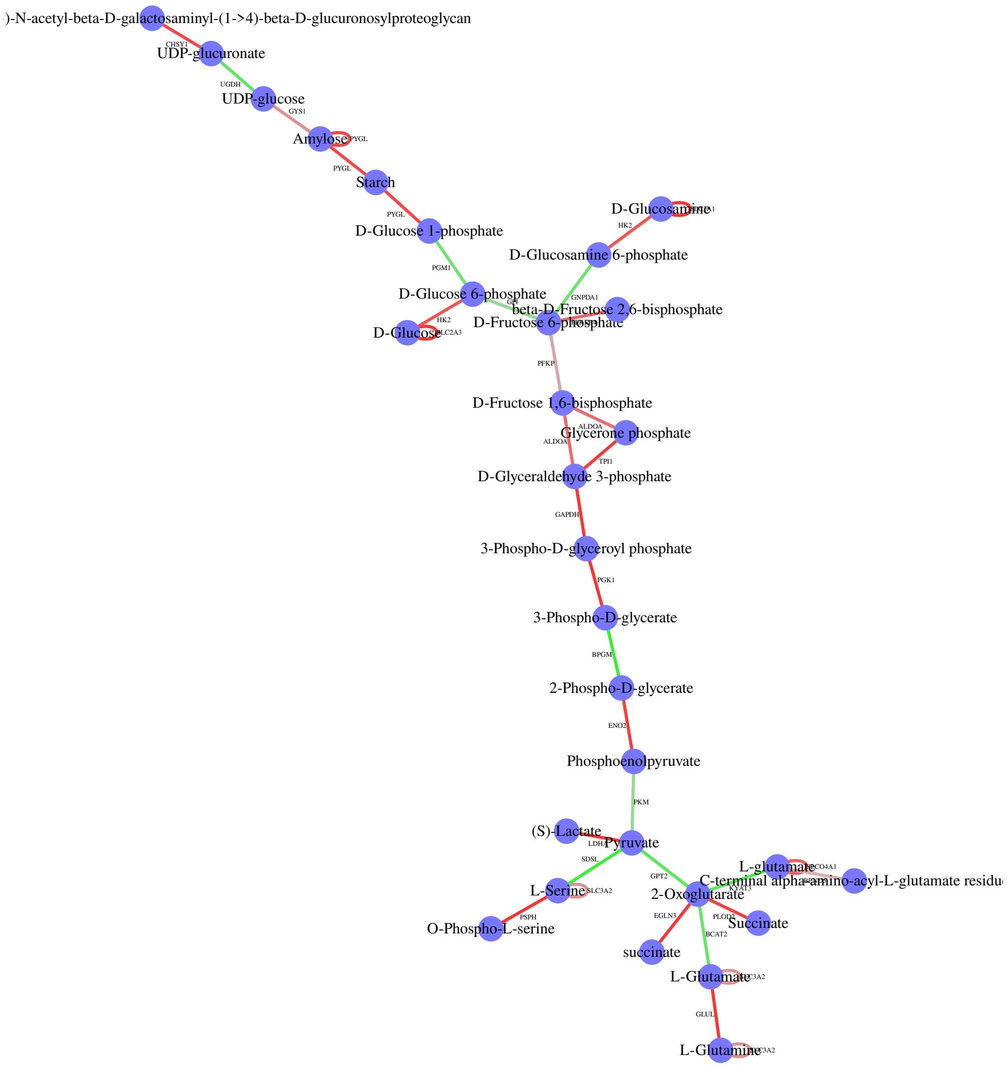

Get gene tables:

The table contains gene list. Each gene has two descriptive values: i) gene's correlation value with the modules pattern and ii) gene's score. High score means that this gene's expression is similar to the module's pattern and not similar to other modules' patterns.

```
m.gene.list <- getGeneTables(modules = results$modules,
                             nets = results$nets,
                             patterns = results$patterns.pos,
                             gene.exprs = E.prep,
                             network.annotation = network.annotation,
                             work.dir = work.dir)
```

```
# Gene tables for module 1 are produced

# Gene tables for module 2 are produced

# Gene tables for module 3 are produced

# Gene tables for module 4 are produced

# Gene tables for module 5 are produced
```

Example of the gene table of the fourth module:

```
head(GAMclust:::read.tsv(file.path(work.dir, "m.4.genes.tsv"))) |>
  kableExtra::kable() |>
  kableExtra::kable_styling()
```

symbol	Entrez	score	cor
SLC2A3	6515	3.332874	0.9552393
SLC2A1	6513	2.693944	0.9334521
EGLN3	112399	2.616592	0.9263654
PGK1	5230	2.520763	0.9213604
GLUL	2752	2.515743	0.9211180
PSPH	5723	2.469190	0.9194949

Get plots of patterns:

```
for(i in 1:length(m.gene.list)){

  print(fgsea::plotCoregulationProfileSpatial(m.gene.list[[i]],
                                              seurat_object,
                                              title = paste0("module ", i)))
}
```

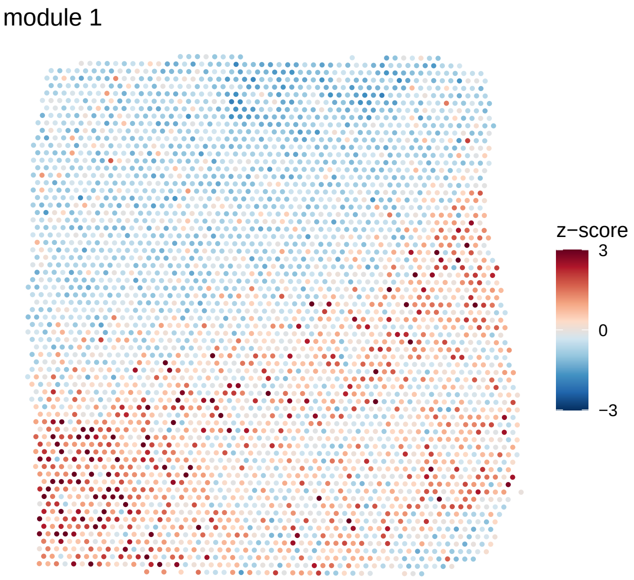

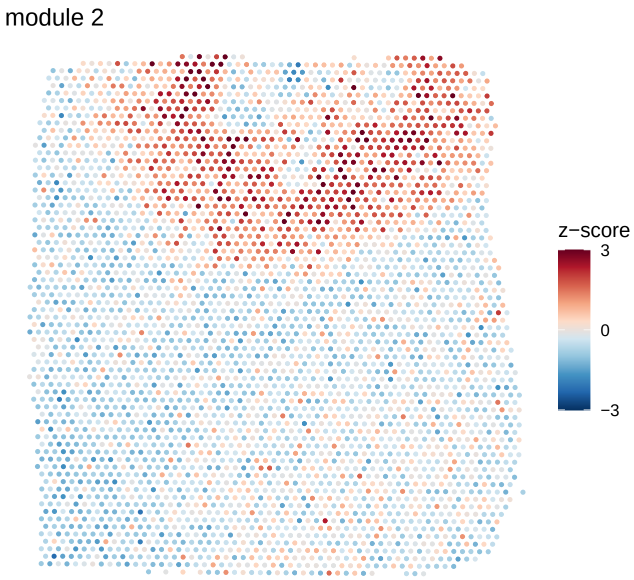

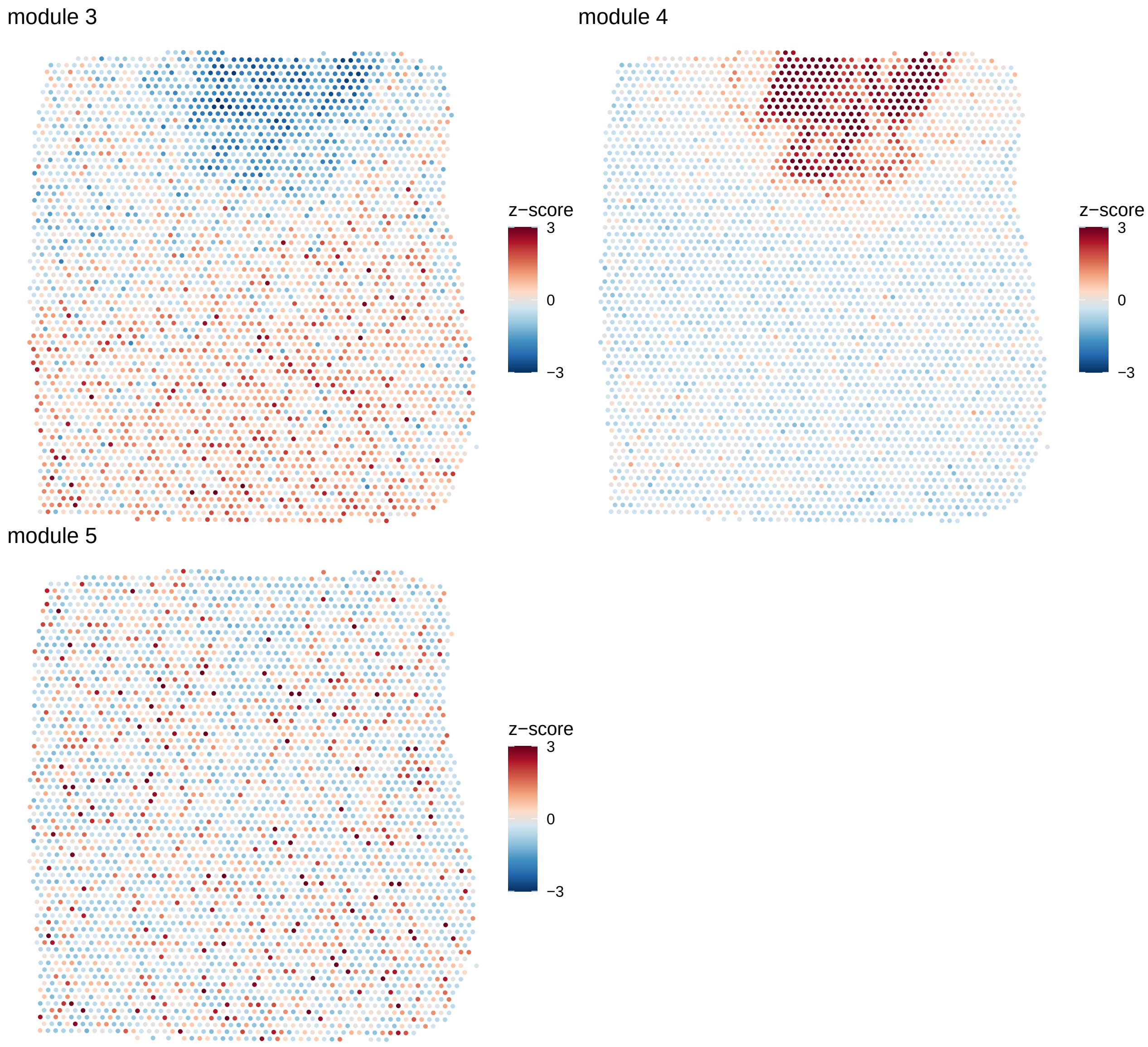

Get plots of individual genes expression (example for the fourth module):

```
Seurat::DefaultAssay(seurat_object) <- "SCT"

i <- 4

Seurat::SpatialFeaturePlot(seurat_object,
                           features = m.gene.list[[i]],
                           pt.size.factor = 2, stroke = 0.01, alpha = 1,
                           image.alpha = 0,
                           ncol = 8)
```

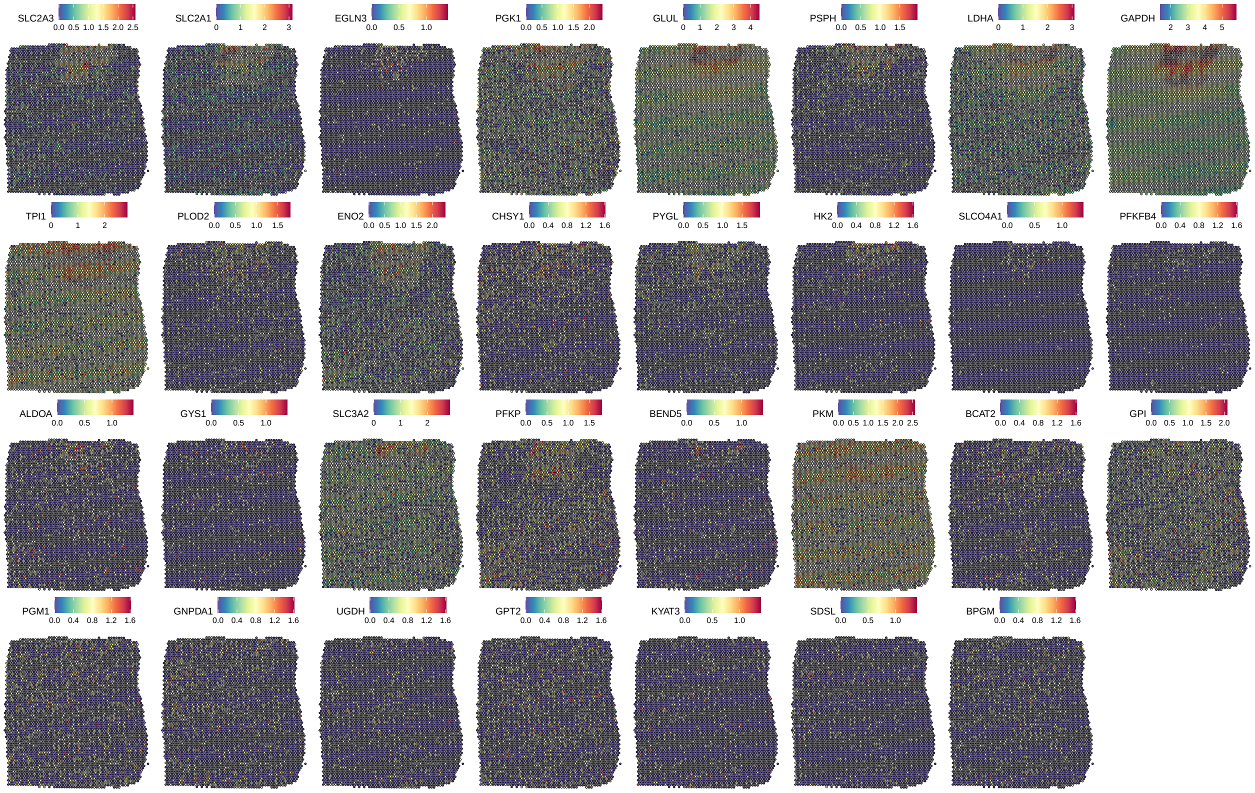

Get tables and plots with annotation of modules:

Functional annotation of obtained modules is done based on KEGG and Reactome canonical metabolic pathways.

```
getAnnotationTables(network.annotation = network.annotation,
                    nets = results$nets,
                    work.dir = work.dir)
```

```
# Pathway annotation for module 1 is produced

# Pathway annotation for module 2 is produced

# Pathway annotation for module 3 is produced

# Pathway annotation for module 4 is produced

# Pathway annotation for module 5 is produced
```

Example of the annotation table of the fourth module:

```
head(GAMclust:::read.tsv(file.path(work.dir, "m.4.pathways.tsv"))) |>
  kableExtra::kable() |>
  kableExtra::kable_styling(font_size = 8) |>
  kableExtra::column_spec(1, width = "1.6in") |>
  kableExtra::column_spec(2:6, width = "0.5in") |>
  kableExtra::column_spec(7, width = "1.2in")
```

pathway	pval	padj	foldEnrichment	overlap	size	overlapGenes

R-HSA-70171: Glycolysis	0.0000000	0.0000000	16.913151	12	13	GNPDA1 ENO2 ALDOA GAPDH GPI HK2 PFKFB4 PFKP PGK1 PKM BPGM TPI1
hsa00500: Starch and sucrose metabolism	0.0000727	0.0043248	9.161290	5	10	GPI GYS1 HK2 PGM1 PYGL
hsa_M00570: Isoleucine biosynthesis, threonine => 2-oxobutanoate => isoleucine	0.0028877	0.0916362	18.322581	2	2	SDSL BCAT2
R-HSA-196836: Vitamin C (ascorbate) metabolism	0.0083672	0.1896560	12.215054	2	3	SLC2A1 SLC2A3
R-HSA-71240: Tryptophan catabolism	0.0161645	0.3205964	9.161290	2	4	KYAT3 SLC3A2
hsa00220: Arginine biosynthesis	0.0377188	0.6191088	6.107527	2	6	GLUL GPT2

Annotation heatmap for all modules:

```
getAnnotationHeatmap(work.dir = work.dir)
```

```
# Processing module 1
# Module size: 43
# Processing module 2
# Module size: 42
# Processing module 3
# Module size: 34
# Processing module 4
# Module size: 31
# Processing module 5
# Module size: 6
```

Compare modules obtained in different runs:

You may also compare two results of running GAM-clustering on the same dataset (e.g. runs with different parameters) or compare two results of running GAM-clustering on different datasets (then set `same.data=FALSE`).

```
modulesSimilarity(dir1 = work.dir,
                  dir2 = "old_dir",
                  name1 = "new",
                  name2 = "old",
                  same.data = TRUE,
                  use.genes.with.pos.score = TRUE,
                  work.dir = work.dir,
                  file.name = "comparison.png")
```

Session info

Elapsed time: 33.2 mins.

Peak R memory usage: 4.3 GB

```r
sessionInfo()
```

```
# R version 4.5.3 (2026-03-11)
# Platform: x86_64-pc-linux-gnu
# Running under: Debian GNU/Linux 13 (trixie)
#
# Matrix products: default
# BLAS:   /usr/lib/x86_64-linux-gnu/openblas-pthread/libblas.so.3
# LAPACK: /usr/lib/x86_64-linux-gnu/openblas-pthread/libopenblasp-r0.3.29.so;  LAPACK
↪  version 3.12.0
#
# locale:
#  [1] LC_CTYPE=C.utf8       LC_NUMERIC=C           LC_TIME=C.utf8
↪  LC_COLLATE=C.utf8     LC_MONETARY=C.utf8
#  [6] LC_MESSAGES=C.utf8    LC_PAPER=C.utf8       LC_NAME=C              LC_ADDRESS=C
↪  LC_TELEPHONE=C
# [11] LC_MEASUREMENT=C.utf8 LC_IDENTIFICATION=C
#
# time zone: America/Chicago
# tzcode source: system (glibc)
#
# attached base packages:
# [1] stats     graphics  grDevices utils     datasets  methods   base
#
# other attached packages:
#  [1] future_1.70.0       Seurat_5.5.0        SeuratObject_5.4.0  sp_2.2-1
↪  futile.logger_1.4.9
#  [6] fgsea_1.39.4        mwcsr_0.1.12        gatom_1.8.4         GAMclust_0.1.0
↪  data.table_1.18.4
# [11] rmarkdown_2.31      rlang_1.3.0         devtools_2.5.0      usethis_3.2.1
#
# loaded via a namespace (and not attached):
#   [1] RcppAnnoy_0.0.23            splines_4.5.3               later_1.4.8
↪  tibble_3.3.1
#   [5] polyclip_1.10-7             graph_1.88.1                XML_3.99-0.23
↪  fastDummies_1.7.6
#   [9] lifecycle_1.0.5             globals_0.19.1              lattice_0.22-7
↪  MASS_7.3-65
#  [13] magrittr_2.0.5              plotly_4.12.0               yaml_2.3.12
↪  httpuv_1.6.17
#  [17] otel_0.2.0                  glmGamPoi_1.22.0            sctransform_0.4.3
↪  spam_2.11-3
#  [21] sessioninfo_1.2.3           pkgbuild_1.4.8              spatstat.sparse_3.1-0
↪  reticulate_1.46.0
#  [25] cowplot_1.2.0               pbapply_1.7-4               DBI_1.3.0
↪  RColorBrewer_1.1-3
#  [29] abind_1.4-8                 pkgload_1.5.2               GenomicRanges_1.62.1
↪  Rtsne_0.17
#  [33] purrr_1.2.2                 BiocGenerics_0.56.0         IRanges_2.44.0
↪  S4Vectors_0.48.1
#  [37] ggrepel_0.9.8               irlba_2.3.7                 listenv_0.10.1
↪  spatstat.utils_3.2-3
#  [41] pheatmap_1.0.13             goftest_1.2-3               RSpectra_0.16-2
↪  spatstat.random_3.4-5
```

```
#  [45] fitdistrplus_1.2-6          parallelly_1.47.0           DelayedMatrixStats_1.32.0
↪  svglite_2.2.2
#  [49] DelayedArray_0.36.1         codetools_0.2-20            xml2_1.5.2
↪  tidyselect_1.2.1
#  [53] farver_2.1.2                shinyCyJS_1.0.0             matrixStats_1.5.0
↪  stats4_4.5.3
#  [57] spatstat.explore_3.8-0      Seqinfo_1.0.0               jsonlite_2.0.0
↪  ellipsis_0.3.2
#  [61] progressr_0.19.0            ggridges_0.5.7              survival_3.8-3
↪  systemfonts_1.3.1
#  [65] tools_4.5.3                 ica_1.0-3                   Rcpp_1.1.2
↪  glue_1.8.1
#  [69] SparseArray_1.10.10         gridExtra_2.3               xfun_0.60
↪  MatrixGenerics_1.22.0
#  [73] dplyr_1.2.1                 withr_3.0.3                 formatR_1.14
↪  fastmap_1.2.0
#  [77] GGally_2.4.0                digest_0.6.39               R6_2.6.1
↪  mime_0.13
#  [81] textshaping_1.0.4           scattermore_1.2             rsvg_2.7.0
↪  tensor_1.5.1
#  [85] spatstat.data_3.1-9         RSQLite_3.53.3              tidyr_1.3.2
↪  generics_0.1.4
#  [89] S4Arrays_1.10.1             httr_1.4.8                  htmlwidgets_1.6.4
↪  ggstats_0.13.0
#  [93] uwot_0.2.4                  pkgconfig_2.0.3             gtable_0.3.6
↪  rsconnect_1.7.0
#  [97] blob_1.3.0                  lmtest_0.9-40               S7_0.2.2
↪  XVector_0.50.0
# [101] htmltools_0.5.9             dotCall64_1.2               BioNet_1.70.0
↪  scales_1.4.0
# [105] kableExtra_1.4.0            Biobase_2.70.0              png_0.1-9
↪  spatstat.univar_3.1-7
# [109] knitr_1.51                  lambda.r_1.2.4              rstudioapi_0.17.1
↪  reshape2_1.4.5
# [113] nlme_3.1-168                cachem_1.1.0                zoo_1.8-15
↪  stringr_1.6.0
# [117] KernSmooth_2.23-26          parallel_4.5.3              miniUI_0.1.2
↪  AnnotationDbi_1.72.0
# [121] pillar_1.11.1               grid_4.5.3                  vctrs_0.7.3
↪  RANN_2.6.2
# [125] promises_1.5.0              beachmat_2.26.0             xtable_1.8-8
↪  cluster_2.1.8.1
# [129] Rgraphviz_2.54.0            evaluate_1.0.5              tinytex_0.60
↪  cli_3.6.6
# [133] compiler_4.5.3              futile.options_1.0.1        crayon_1.5.3
↪  future.apply_1.20.2
# [137] labeling_0.4.3              plyr_1.8.9                  fs_2.1.0
↪  stringi_1.8.7
# [141] deldir_2.0-4                viridisLite_0.4.3           BiocParallel_1.44.0
↪  Biostrings_2.78.0
# [145] lazyeval_0.2.3              spatstat.geom_3.7-3         Matrix_1.7-4
↪  RcppHNSW_0.6.0
# [149] patchwork_1.3.2             sparseMatrixStats_1.22.0    bit64_4.8.2
↪  ggplot2_4.0.3
```

```
# [153] KEGGREST_1.53.5             shiny_1.13.0
 ↪  SummarizedExperiment_1.40.0 ROCR_1.0-12
# [157] igraph_2.3.3                memoise_2.0.1                fastmatch_1.1-8
 ↪  bit_4.6.0
```
