# Supplementary material for: GAMclust: identification of regulated metabolic modules in bulk, single cell and spatial gene expression data
Source: Bioinformatics. 2026 Aug 22;42(9):btag639. doi: 10.1093/bioinformatics/btag639 (PMC13544963; doi:10.1093/bioinformatics/btag639)
Supplement: Supplementary file 1 [file btag639_supplementary_data.zip › S6_GAMclust_on_single_cell_RNA-seq_Friedrich_et_al_dataset.pdf]

# Supplementary File 6: Metabolic Profiling of Friedrich et al glioma dataset with GAMclust

## Contents

**Introduction** **1**

**Input dataset** **1**

**Analysis** **2**
- Preparing working environment . . . . . 2
- Preparing objects for the analysis . . . . . 3
- GAM-clustering analysis . . . . . 4
- Visualizing and exploring the GAM-clustering results . . . . . 5

**Discussion** **11**

```
library(GAMclust)
library(gatom)
library(mwcsr)
library(fgsea)
library(data.table)
library(Seurat)
library(futile.logger)
library(ggplot2)
set.seed(42)
```

## Introduction

In this vignette we show the application of the GAM-clustering on single-cell RNA-Seq data.

## Input dataset

Here we use microglial subset of the human scRNA-Seq data from the study of gliomas microenvironment.

```
seurat_object <- readRDS("data.rds")
```

This dataset consist of control, IDH wild-type glioma and IDH mutant glioma samples.

```
Seurat::DimPlot(seurat_object, split.by = "Diagnosis", group.by = "integrated_snn_res.0.6
↪  ")
```

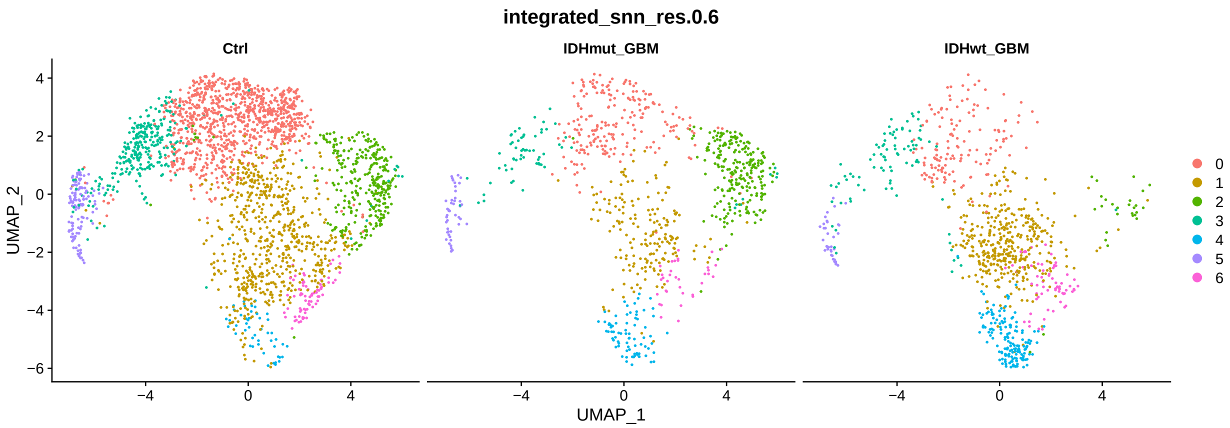


# Analysis

## Preparing working environment

First, we load and initialize all objects required for GAM-clustering analysis:

(1) load metabolic network

```
network <- readRDS(url("http://artyomovlab.wustl.edu/publications/supp_materials/GATOM/ne
↪   twork.combined.rds"))
metabolites.annotation <- readRDS(url("http://artyomovlab.wustl.edu/publications/supp_mat
↪   erials/GATOM/met.combined.db.rds"))
gene2reaction.extra <- data.table::fread("http://artyomovlab.wustl.edu/publications/supp_
↪   materials/GATOM/gene2reaction.combined.hsa.eg.tsv", colClasses="character")
```

(2) load species-specific network annotation: `org.Hs.eg.gatom.anno` for human data or `org.Mm.eg.gatom.anno` for mouse data;

```
network.annotation <- readRDS(url("http://artyomovlab.wustl.edu/publications/supp_materia
↪   ls/GATOM/org.Hs.eg.gatom.anno.rds"))
```

(3) load provided list of metabolites that should not be considered during the analysis as connections between reactions (e.g., CO2, HCO3-, etc);

```
met.to.filter <- data.table::fread(system.file("mets2mask.lst", package="GAMclust"))$ID
```

(4) initialize proprietary CPLEX solver (free for academy);

```
cplex.dir <-  "/opt/ibm/ILOG/CPLEX_Studio1271"
```

```
solver <- mwcsr::virgo_solver(cplex_dir = cplex.dir)
```

(5) set working directory where the results will be saved to.

```
work.dir <- "results"
dir.create(work.dir, showWarnings = F, recursive = T)
```

(6) Collecting logs.

```
stats.dir <- file.path(work.dir, "stats")
dir.create(stats.dir, showWarnings = F, recursive = T)
```

```
setup_logger <- function(log.file.path, logger.name = "stats.logger") {
  file.appender <- appender.file(log.file.path)
  console.appender <- appender.console()
  combined.appender <- function(line) {
    file.appender(line)
    console.appender(line)
  }
  flog.appender(combined.appender, name = logger.name)
  flog.threshold(TRACE, name = logger.name)
}

log.file <- file.path(stats.dir, "log.txt")
setup_logger(log.file.path = log.file, logger.name = "stats.logger")
```

## Preparing objects for the analysis

### Preparing data

For the analysis we require expression data, here we use normalized and scaled with sctransform

```
E <- as.matrix(Seurat::GetAssayData(object = seurat_object,
                                    assay = "SCT",
                                    layer = "scale.data"))
nrow(E)
```

```
# [1] 8483
```

Table 1: Expression data

| | Pat1_Ctrl_1_2 | Pat1_Ctrl_1_3 | Pat1_Ctrl_1_4 |
|---|---|---|---|
| A2M | -1.0153022 | 0.1082862 | 1.0199891 |
| AAAS | 5.9680111 | 3.2506112 | -0.2568466 |
| AAED1 | -0.2587509 | -0.2231623 | -0.2571140 |

We will use PCA option in the analysis to reduce dimensionality of the single-cell dataset

```
E.prep <- prepareData(E = E,
                      gene.id.type = "Symbol",
                      use.PCA = TRUE,
                      use.PCA.n = 50,
                      network.annotation = network.annotation)
```

Gene symbols are replaced with Entrez IDs and instead of cells now we have PCs.

Table 2: Data ready for the analysis

| | PC1 | PC2 | PC3 | PC4 | PC5 |
|---|---|---|---|---|---|
| 342035 | 1.633064 | 3.117240 | 1.7491111 | 0.7708168 | -3.0763842 |
| 10964 | 1.271353 | 1.561451 | 0.7613911 | 3.9839888 | 0.2625252 |
| 348 | 3.245142 | 4.426633 | 0.3624805 | 1.4658940 | -1.5026112 |

### Preparing network

```
network.prep <- prepareNetwork(E = E.prep,
                               network = network,
                               topology = "metabolites",
                               met.to.filter = met.to.filter,
                               network.annotation = network.annotation,
                               gene2reaction.extra = gene2reaction.extra)
```

```
# INFO [2026-07-17 19:37:22] Global metabolite network contains 4615 edges.
# INFO [2026-07-17 19:37:22] Largest connected component of this global network contains
↪  1157 nodes and 3462 edges.
```

### Preclustering

Following the recommendations we set the number of initial clusters to 32

```
cur.centers <- preClustering(E.prep = E.prep,
                             network.prep = network.prep,
                             initial.number.of.clusters = 32,
                             network.annotation = network.annotation)
```

```
# INFO [2026-07-17 19:37:22] 792 metabolic genes from the analysed dataset mapped to this
↪  component.
```

```
pheatmap::pheatmap(
      GAMclust:::normalize.rows(cur.centers),
      cluster_rows=F, cluster_cols=F,
      show_rownames=F, show_colnames=T)
```

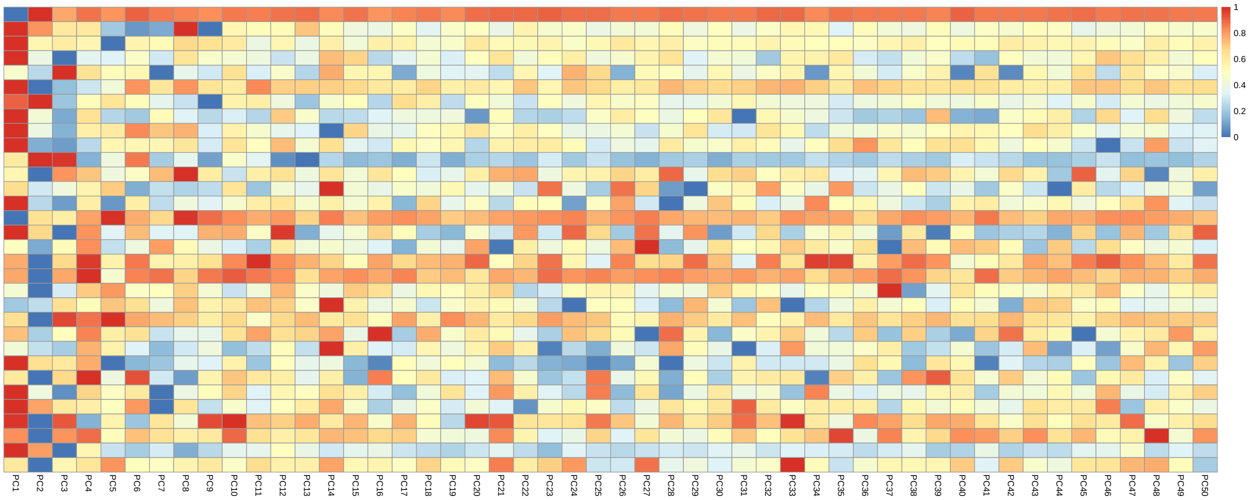

## GAM-clustering analysis

Run GAM-clustering analysis

```
results <- gamClustering(E.prep = E.prep,
                         network.prep = network.prep,
                         cur.centers = cur.centers,

                         start.base = 1,
```

```
                                base.dec = 0.1,
                                max.module.size = 50,

                                cor.threshold = 0.8,
                                p.adj.val.threshold = 1e-5,

                                batch.solver = seq_batch_solver(solver),
                                work.dir = work.dir,

                                show.intermediate.clustering = FALSE,
                                verbose = FALSE,
                                collect.stats = TRUE)
```

**Running time**

Elapsed time: **33 min**

One of the main parameters in GAMclust, *base* parameter, should be adapted for each dataset. It regulates correlation of modules genes expression with overall module expression pattern and ranges from 0 to 1. GAMclust supports automated inference of the *base* parameter. Starting from *start.base* it decreases by following formula: `base * (1 - base.dec)`, - until it reaches the optimal level.

After **43 iterations** and **33 minutes** there are three metabolic modules found for the analysed dataset.

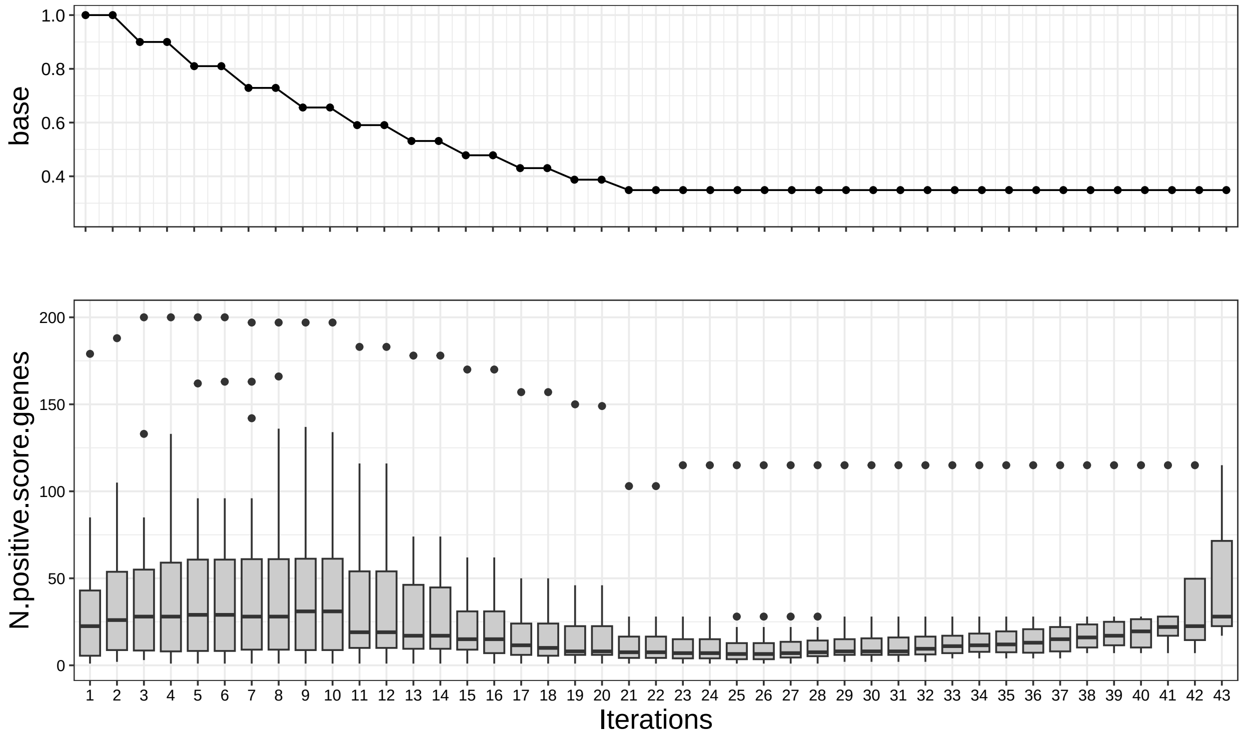


# Visualizing and exploring the GAM-clustering results

**Get graphs of modules:**

```
getGraphs(modules = results$modules,
          network.annotation = network.annotation,
          metabolites.annotation = metabolites.annotation,
```

```
          seed.for.layout = 42,
          work.dir = work.dir)
```

```
# Graphs for module 1 are built

# Graphs for module 2 are built

# Graphs for module 3 are built
```

We got three modules:

- Module 1

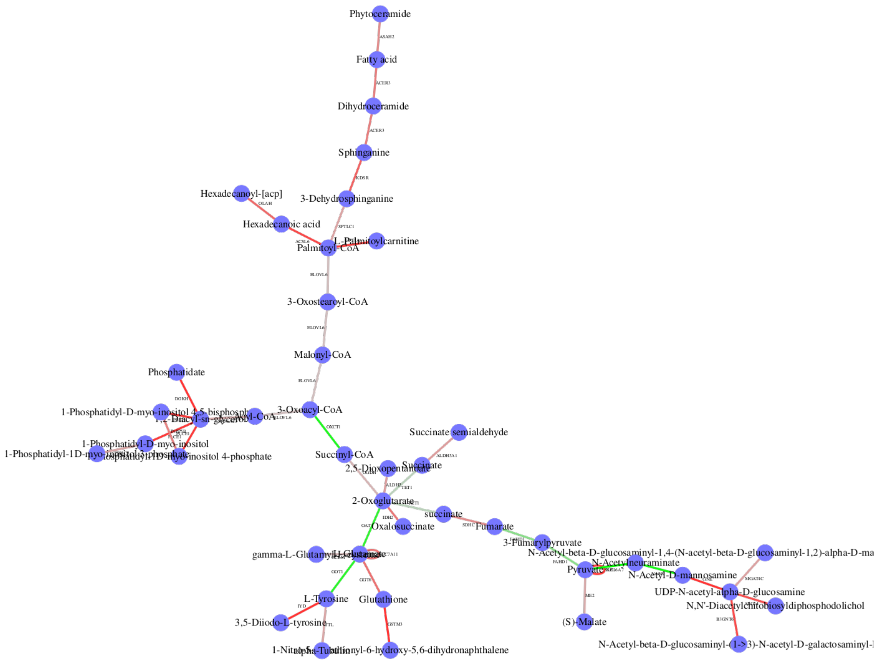


- Module 2

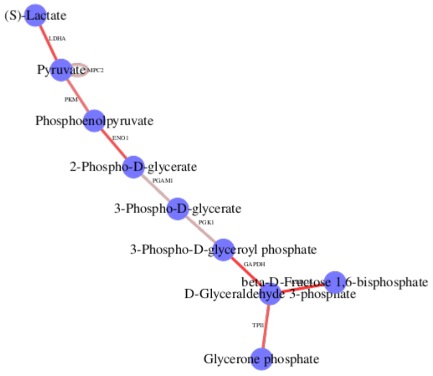


- Module 3

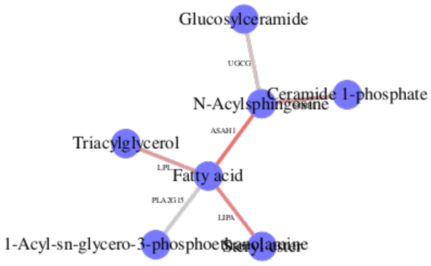

**Get gene tables:**

```
m.gene.list <- getGeneTables(modules = results$modules,
                             nets = results$nets,
                             patterns = results$patterns.pos,
                             gene.exprs = E.prep,
                             network.annotation = network.annotation,
                             work.dir = work.dir)
```

```
# Gene tables for module 1 are produced

# Gene tables for module 2 are produced

# Gene tables for module 3 are produced
```

**Get plots of modules genes and patterns:**

Patterns:

```
Seurat::DefaultAssay(seurat_object) <- "integrated"
geseca_list <- list()
for(i in 1:length(m.gene.list)){
  p <- fgsea::plotCoregulationProfileReduction(m.gene.list[[i]],
                                               seurat_object,
                                               title = paste0("module ", i),
                                               pt.size = 0.5,
                                               reduction = reduction)
  geseca_list[[i]] <- p
}

cowplot::plot_grid(plotlist = geseca_list,
                   ncol = length(m.gene.list))
```

```
ggplot2::ggsave(file = sprintf("%s/m.all.pattern_int.png", work.dir),
                bg = "white",
                dpi = 400,
                width = length(m.gene.list) * 4,
                height = 4)
```

Patterns of obtained modules

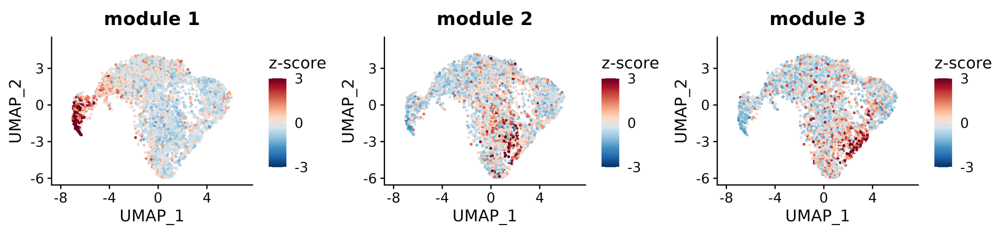


Modules genes expression:

```
Seurat::DefaultAssay(seurat_object) <- "SCT"
for(i in 1:length(m.gene.list)){
  Seurat::FeaturePlot(seurat_object,
```

```
                              reduction = reduction,
                              features = m.gene.list[[i]],
                              ncol = 5,
                              order = T,
                              combine = T)

  ggplot2::ggsave(file = sprintf("%s/m.%s.genes.png", work.dir, i),
                  width = 15,
                  height = round(length(m.gene.list[[i]]) / 5)*2 + 1)
}
```

Example of genes expression for Module 2

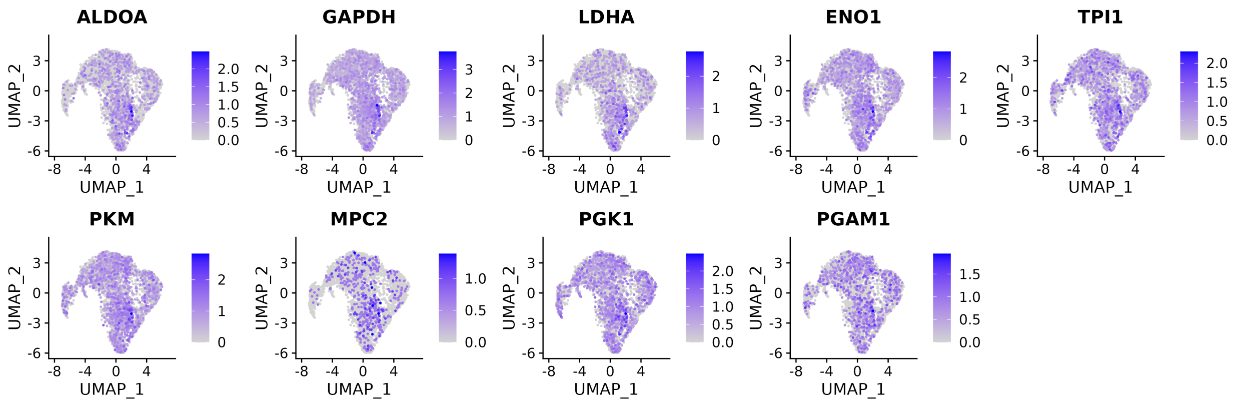


Modules specificity:

```
seurat_object <- AddModuleScore(seurat_object,
                                features = m.gene.list,
                                assay = "SCT", name = "M")
```

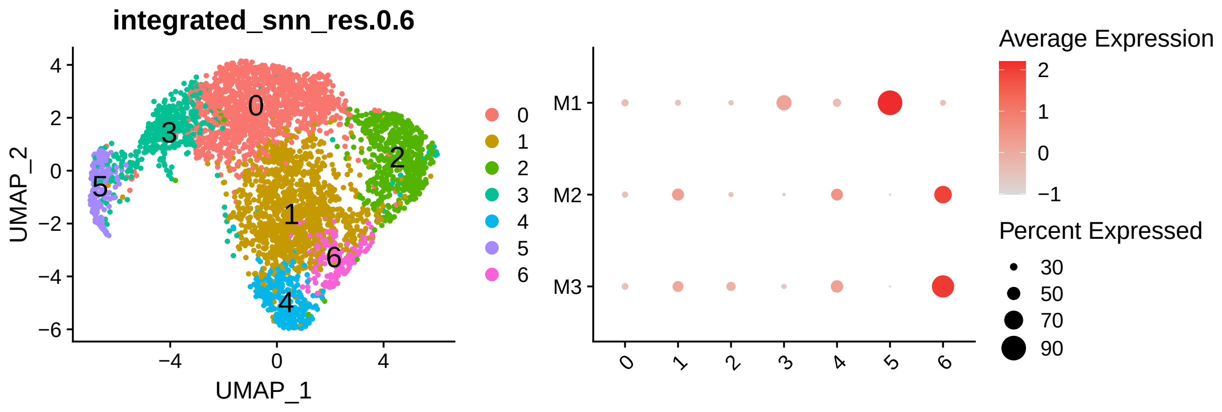

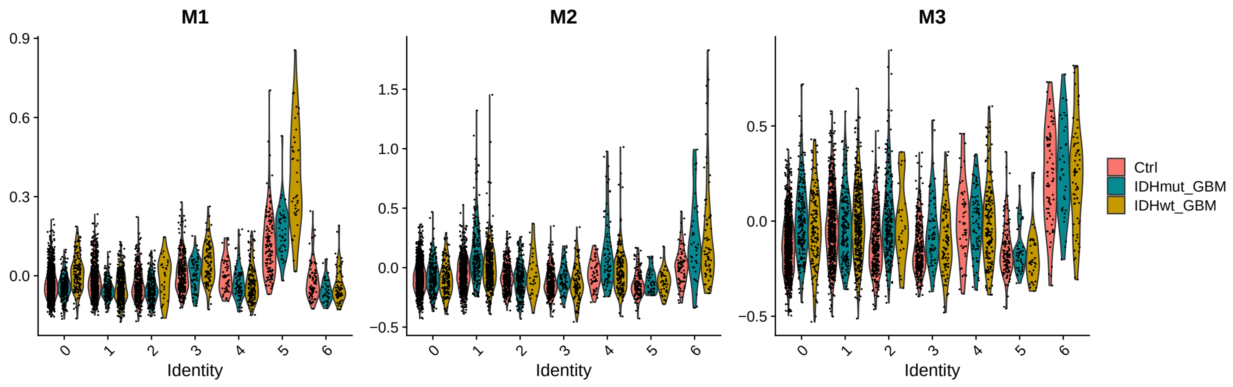


**Module 1**: Module 1 showed the highest score in cluster 5, mainly in IDHwt samples.

**Module 2**: Expression of Module 2 genes is mainly localized to clusters 6, with tumor samples showing higher expression compared to controls.

**Module 3**: Module 3 is cluster 6 specific but does not show differences between conditions.

### Get tables and plots with annotation of modules:

Functional annotation of obtained modules is done based on KEGG and Reactome canonical metabolic pathways.

```
getAnnotationTables(network.annotation = network.annotation,
                    nets = results$nets,
                    work.dir = work.dir)
```

```
# Pathway annotation for module 1 is produced

# Pathway annotation for module 2 is produced

# Pathway annotation for module 3 is produced
```

Example of the annotation table for the second module:

```
tbl <- head(GAMclust:::read.tsv(file.path(work.dir, "m.2.pathways.tsv")))
```

Table 3: Modules annotation

| pathway | pval | padj | foldEnrichment | overlap | size | overlapGenes |
|---|---|---|---|---|---|---|
| hsa_M00001: Glycolysis (Embden-Meyerhof pathway), glucose => pyruvate | 0.0000000 | 0.0000000 | 37.10000 | 7 | 10 | ENO1 ALDOA GAPDH PGAM1 PGK1 PKM TPI1 |
| hsa_M00002: Glycolysis, core module involving three-carbon compounds | 0.0000000 | 0.0000000 | 53.00000 | 6 | 6 | ENO1 GAPDH PGAM1 PGK1 PKM TPI1 |
| hsa_M00003: Gluconeogenesis, oxaloacetate => fructose-6P | 0.0000000 | 0.0000000 | 45.42857 | 6 | 7 | ENO1 ALDOA GAPDH PGAM1 PGK1 TPI1 |
| R-HSA-70268: Pyruvate metabolism | 0.0106528 | 0.4039203 | 11.77778 | 2 | 9 | MPC2 LDHA |

Annotation heatmap for all modules:

```
getAnnotationHeatmap(work.dir = work.dir, file_name = "Modules_heatmap.png")
```

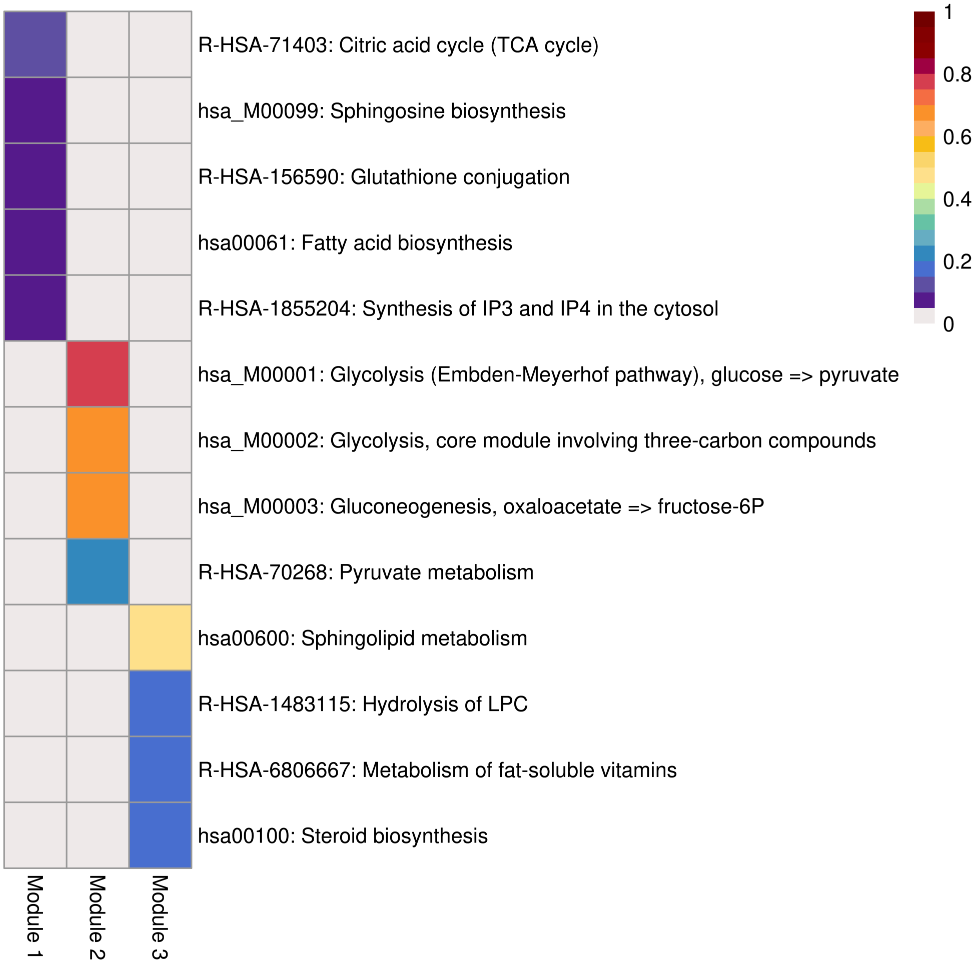


# Discussion

GAM-clustering was able to find two modules devoted the tumor phenotype. Both modules are mainly devoted the energy metabolism and highlight principal difference between IDHwt and IGHmut tumors. Associated with the poor prognosis IDHwt glioma exhibit upregulation of TCA cycle and in both tumors glycolysis appeared to be more active compared to control samples. The third module found by GAMclust did not show a clear diagnosis-specific pattern and appeared to reflect a lipid- and sphingolipid-related metabolic program.
