# Supplementary material for: GAMclust: identification of regulated metabolic modules in bulk, single cell and spatial gene expression data
Source: Bioinformatics. 2026 Aug 22;42(9):btag639. doi: 10.1093/bioinformatics/btag639 (PMC13544963; doi:10.1093/bioinformatics/btag639)
Supplement: Supplementary file 1 [file btag639_supplementary_data.zip › S7_TiCoNE_on_single_cell_RNA-seq_Friedrich_et_al_dataset.pdf]

Supplementary File 6: Metabolic Profiling of Friedrich et al glioma dataset with TiCoNE

Contents

Introduction **2**

Method overview **2**

Input dataset **3**

Analysis **3**

Input files 3

Clustering parameters 4

Clustering 5

Visualization of the results 7

Comparison with the GAM-clustering results 13

Discussion **15**

```r
library(fgsea)
library(data.table)
library(Seurat)
library(ggplot2)
library(dplyr)
library(UpSetR)
set.seed(42)
```

Introduction

There is a tool that provides functionality to infer metabolic modules similar to those obtained with GAM-clustering. However, the process of module generation does not take network structure into account until the final step. In this vignette, we describe the TiCoNE pipeline and compare its results with the GAM-clustering modules.

Method overview

TiCoNE (**Ti**me **Co**urse **N**etwork **E**nrichment) is a human-augmented clustering method for time series data combined with a temporal network enricher.

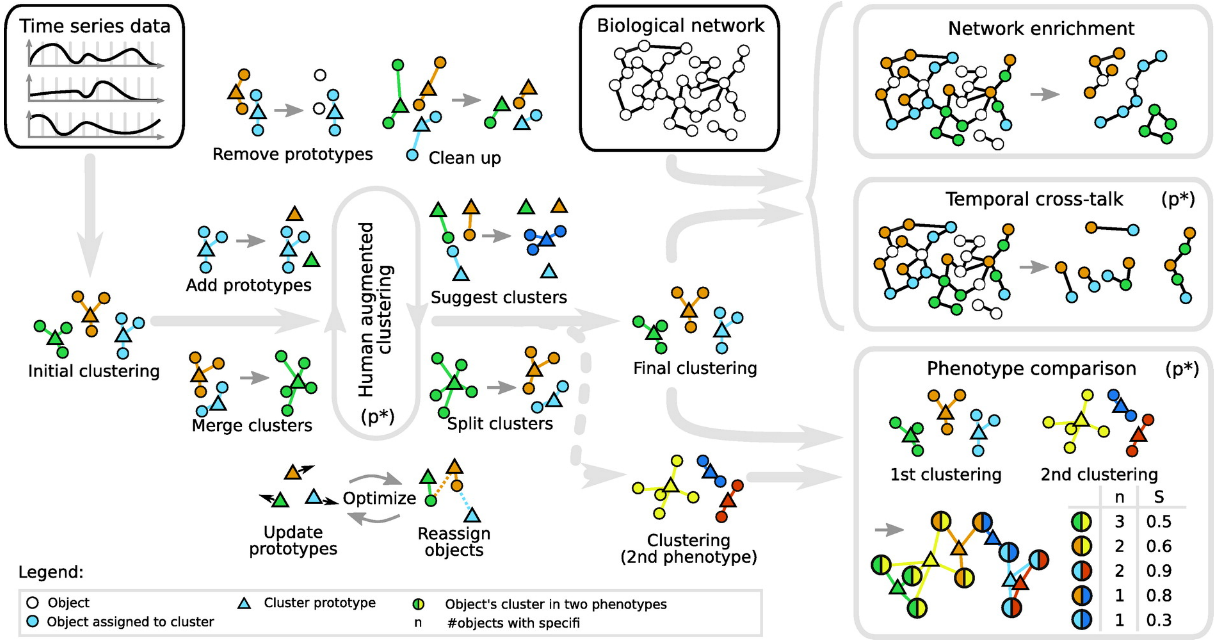

Wiwie C, Kuznetsova I, Mostafa A, et al. Time-Resolved Systems Medicine Reveals Viral Infection-Modulating Host Targets. Syst Med (New Rochelle). 2019;2(1):1-9. Published 2019 Mar 28.

doi:10.1089/sysm.2018.0013

- Gene clustering is performed based on the similarity of temporal expression profiles using multiple methods of choice
- The tool supports interactive user-guided refinement of clusters to incorproate prior knowledge or improve biological relevance
- The final output of TiCoNE integrates dynamic expression patterns with biological network structure, resulting in gene modules that are both temporally coherent and functionally connected

Input dataset

Similar to the GAM-clustering analysis vignette we show the full analysis pipeline on the glioma dataset consisting of control and two tumor sample groups (IDH wild-type and mutant samples)

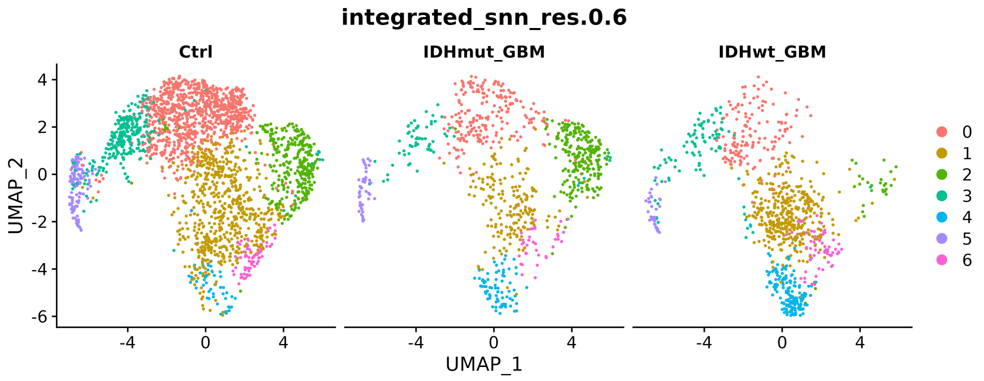

Analysis

Input files

- As input data we provided TiCoNE the same matrix of principle components that was used in the GAM-clustering analysis. It worth noting that TiCoNE was designed to work with bulk expression data and doesn't directly support PCA-based clustering. Although there might be unexpected consequences of our choice inn this analysis we use the same PC matrix as in GAM-clustering analysis for direct comparison of methods perfomance.

```
gam_results <- "GAMclust/results"
```

```
E.prep <- read.csv(
  file.path(gam_results, "E.prep_filt.tsv"),
  sep = "\t") %>%
  select(!Gene_Entrez) %>%
  tibble::column_to_rownames("Symbol")
```

Table 1: Input PC matrix

	PC1	PC2	PC3	PC4	PC5	PC6
GNPDA1	2.6717631	1.3852327	-0.6692550	0.4663673	-0.1212856	0.1379687
HDAC6	-0.1968200	-1.3577572	1.1242218	1.2622552	-0.7792255	0.5956046
HDAC5	0.6109927	-0.8915012	0.2995126	1.1243103	-0.3237144	-0.1737234

- Biological network

The same network that was used in the GAM-clustering analysis but with reversed edges and vertices to match TiCoNE requirements

Table 2: Network

from	to	name
DCXR	SORD	Xylitol
ADH5	AKR1A1	Acetaldehyde
ADH5	AKR1A1	Aldehyde

Clustering parameters

Main clustering parameters:

- Clustering method: k-means
- Initial number of clusters: 8
- Prototypes aggregation function: Mean time series
- Similarity estimate: Pearson correlation
- Random seed: 42 (for reproducibility)

At the first step, gene clustering, TiCoNE requires initial number of clusters. To select appropriate number of clusters we performed initial TiCoNE clustering step for n from 2 to 32. Based on pairwise correlation distance we selected n=8 as initial n.

```
ticone_initial_clustering <- "Clusterings_seed42"
```

Calculate pairwise correlation distance

```
cor_list <- list()
cor_list_df <- data.frame()
for (i in c(2:32)) {
  cl <- read.csv(
    file.path(ticone_initial_clustering, sprintf("cl_%s.tsv", i)),
    sep = "\t", col.names = c("cluster", "gene"), header = F)
  df_i <- data.frame()
  for (cl_i in stringr::str_sort(unique(cl$cluster), numeric = T)) {
    genes <- (cl %>% filter(cluster == cl_i))$gene
    E_mean <- E.prep[genes,] %>%
      colMeans(na.rm = T) %>% as.data.frame() %>% t() %>%
      as.data.frame() %>% `rownames<-`(gsub(" ", "_", cl_i))
    df_i <- rbind(df_i, E_mean)
  }
  df_cor <- as.data.frame(cor(t(df_i), method = "pearson"))
  cor_list[[as.character(i)]] <- df_cor
  cor_list_df <- rbind(cor_list_df,
                       data.frame(value = cor_list[[as.character(i)]][
                         lower.tri(cor_list[[as.character(i)]])], clusters = i))
}
```

```
ggplot(cor_list_df, aes(x = clusters, y = value,
                        group = clusters)) +
  geom_boxplot(outliers = F) +
  geom_point(position = position_jitter(width = 0.2), size = 0.3) +
  theme_bw() +
  scale_x_continuous(breaks = seq(2,32,1))
```

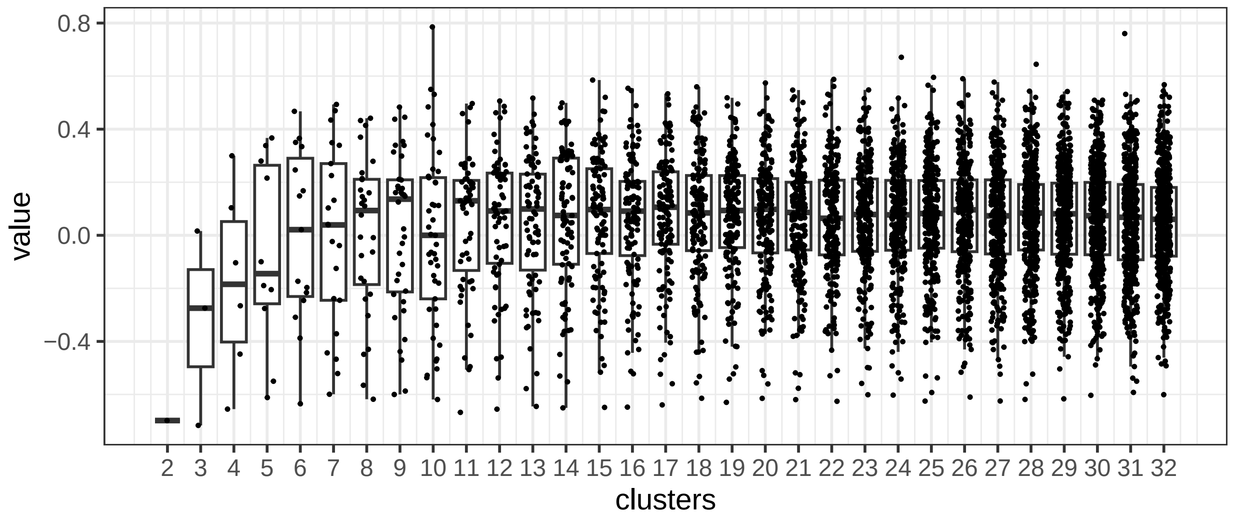

clusters - n, number of clusters that was provided to TiCoNE initial clustering step; value - pairwise pearson correlation distance between clusters, calculated for the averaged values

TiCoNE interface in Cytoscape with loaded biological network and data. Here we show all the paramters that were set before the initial clustering step.

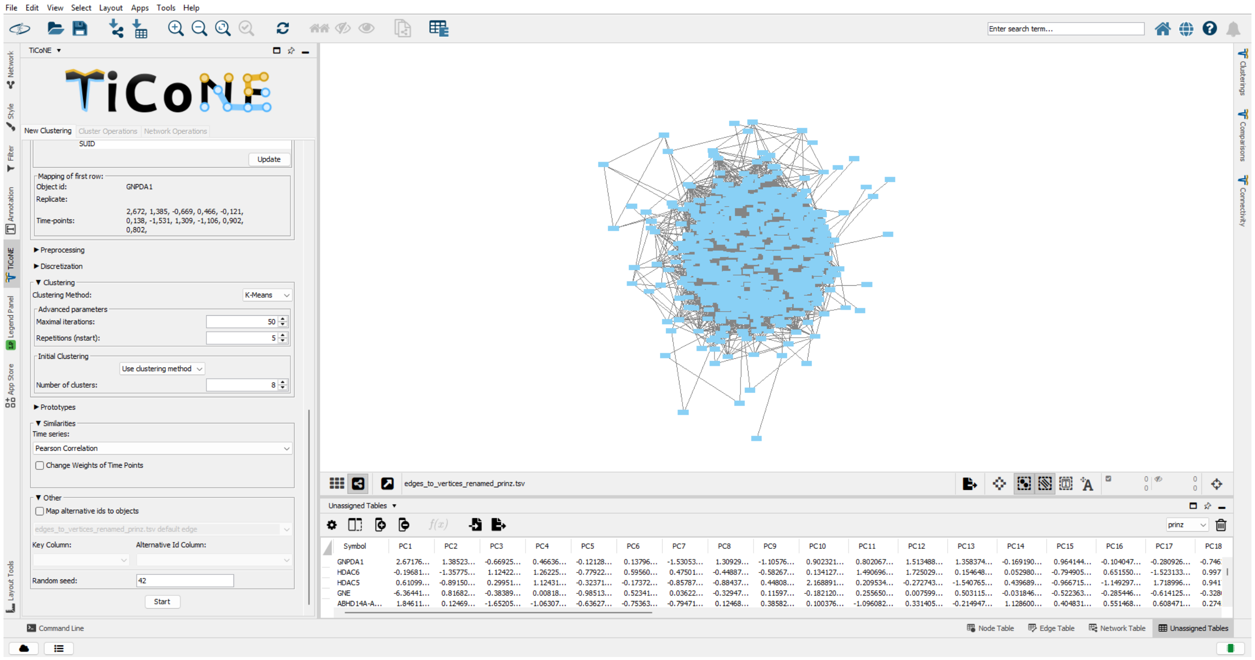

Clustering

After initial clustering we proceeded to final clusters using cluster “Until Convergance” functionality. As a result after 5 clustering iterations we obtained 8 final clusters and calculated p-values with default settings and specified earlier random seed for each cluster.

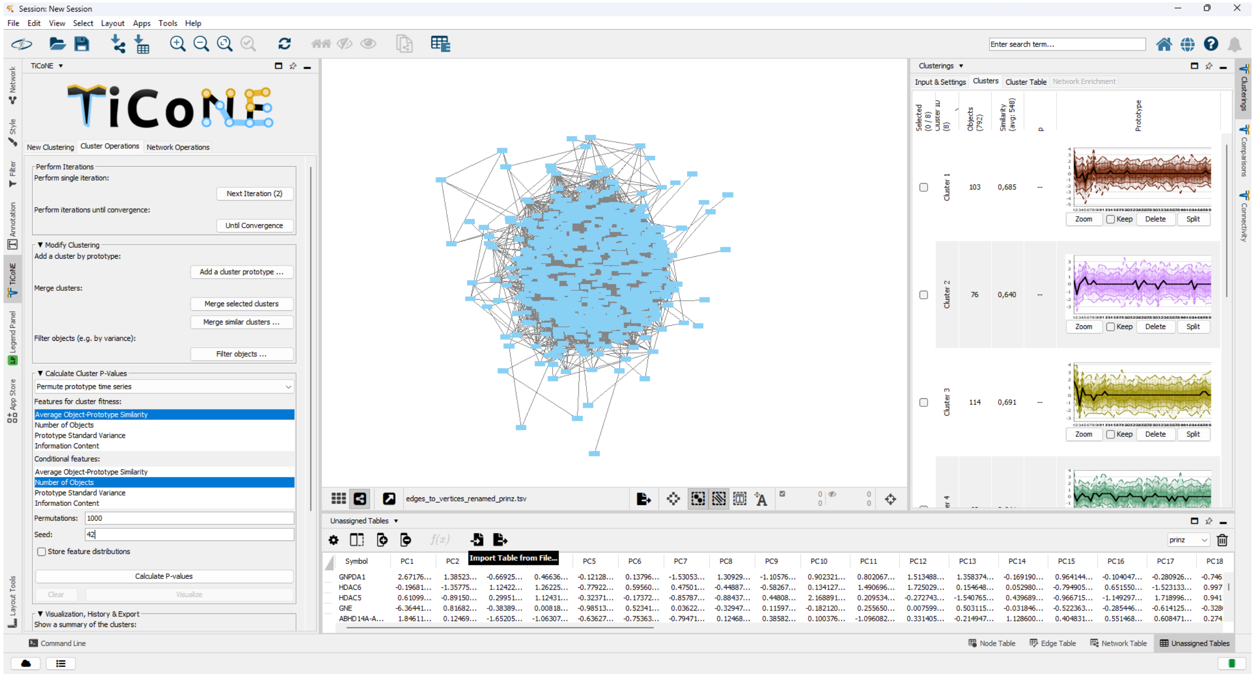

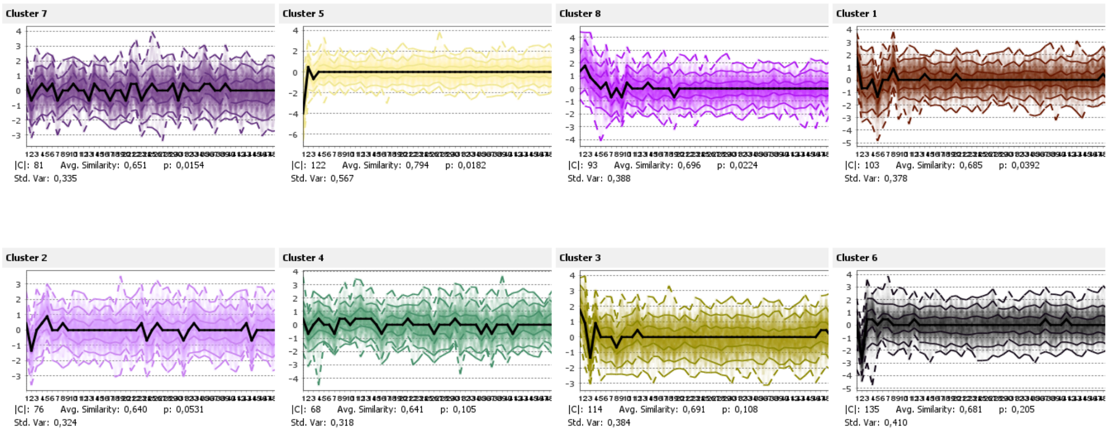

Clusters 1, 5, 7 and 8 showed statistical significance and were considered as final resulting modules.

Running time

Each step: **<1 min**

Overall required time: **<30 min** (highly depends on the dataset and user experience)

Visualization of the results

For each cluster we show here corresponding network with genes as vertices and biological interpretation of the results.

- Cluster 1

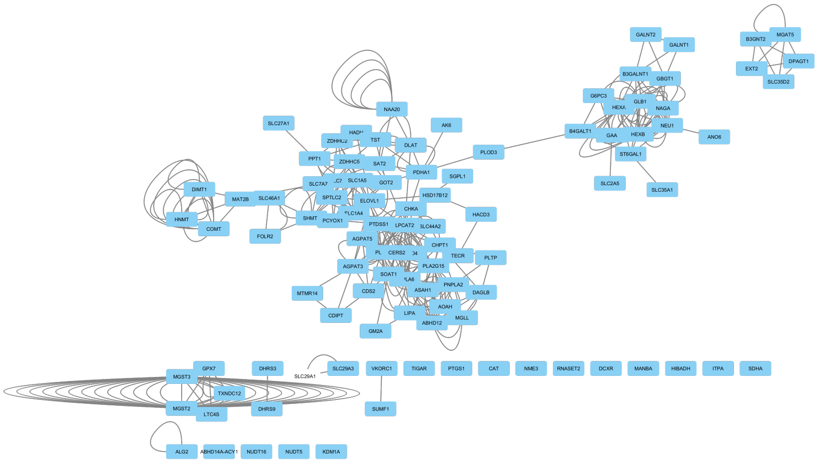

- Cluster 5

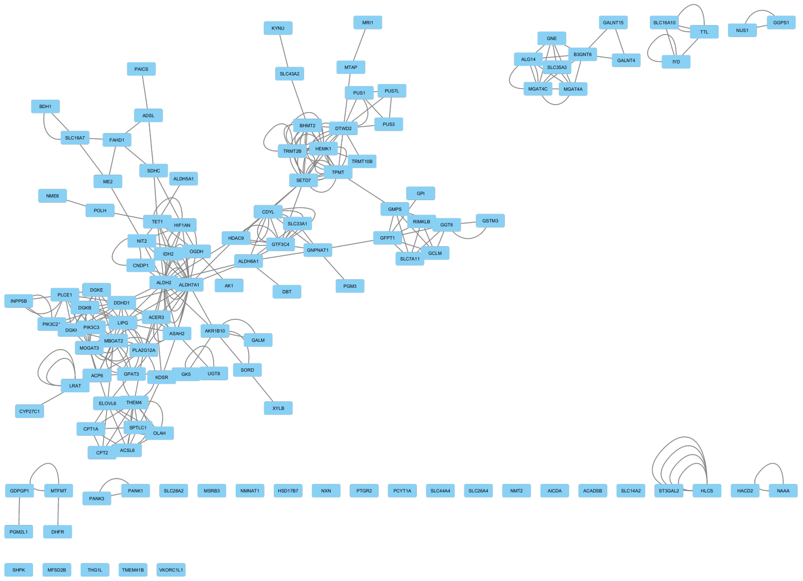

- Cluster 7

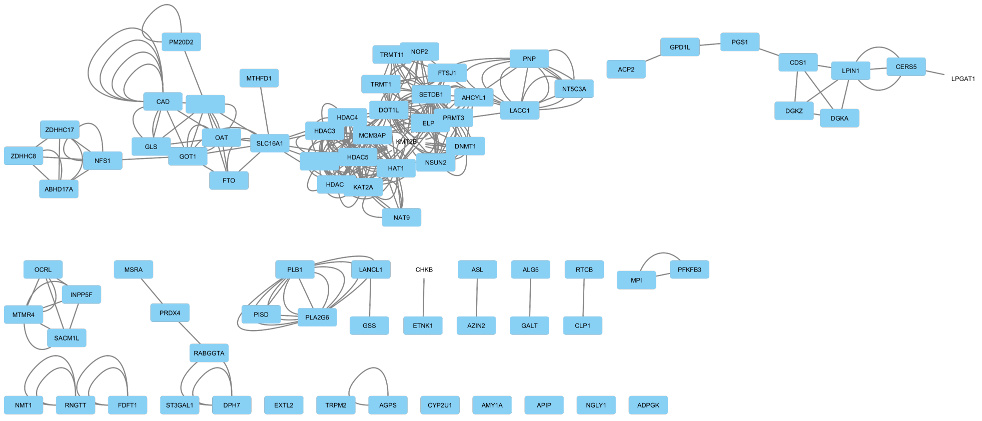

* Cluster 8

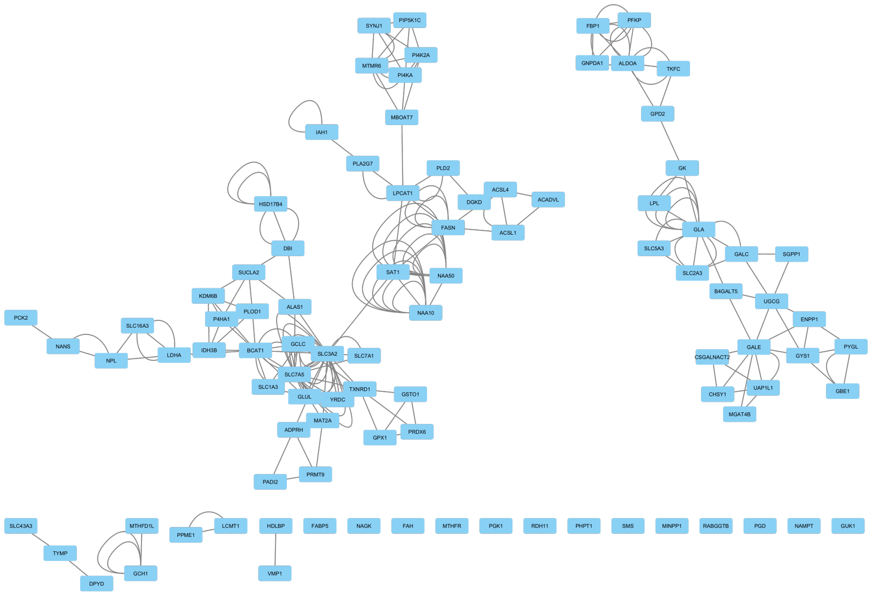

Annotation of the resulting modules

Annotation was performed similar to the GAM-clustering modules annotation.

```
ticone_clustering <- "Clusterings_8_seed42"
```

Load all objects form the GAM-clustering analysis

```
load(file.path(gam_results, "objects.RData"))
```

In the next sections we will obtain gene content of TiCoNE modules, visualize their expression pattern and perform pathways enrichment analysis separately for the whole module content and the largest connected subnetwork

- **Whole modules content**

```
clusters <- GAMclust:::read.tsv(file.path(ticone_clustering, "final_clusters.tsv"),
                                header = F)
colnames(clusters) <- c("Cluster", "Gene")
# keep only significant (1,3,7)
clusters <- clusters %>%
  filter(Cluster %in% paste0("Cluster ", c(1,5,7,8))) %>%
  mutate(Cluster = gsub(" ", "_", Cluster))
# convert symbols to entrez
symbols <- network.annotation[["mapFrom"]][["Symbol"]]
data.table::setkey(symbols, "Symbol")
symbols <- symbols[clusters$Gene,]
clusters$Entrez <- symbols$gene
```

```
clusters_split <- split(clusters, clusters$Cluster)
geseca_list <- list()
for(i in seq_along(clusters_split)){
```

```r
  Seurat::DefaultAssay(seurat_object) <- "integrated"
  p <- fgsea::plotCoregulationProfileReduction(clusters_split[[i]]$Gene, seurat_object,
                                                order = T,
                                                reduction = "umap") +
    ggplot2::ggtitle(paste0("Module_", gsub("Cluster_(\\d+)", "\\1",
↪   names(clusters_split)[i])))
  geseca_list[[i]] <- p
}
cowplot::plot_grid(plotlist = geseca_list,
                   ncol = 4)
```

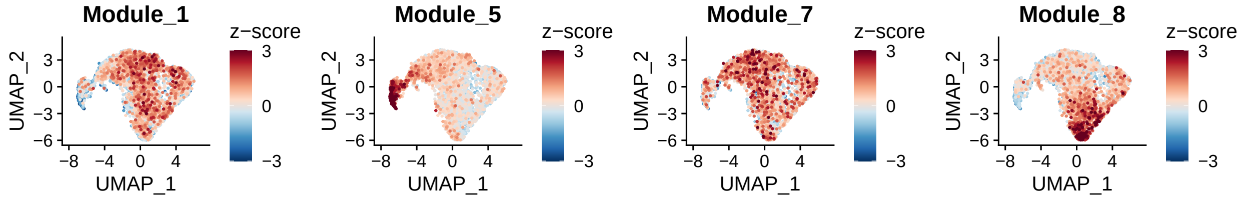

Geseca scores:

```r
DefaultAssay(seurat_object) <- "integrated"
seurat_object <- RunPCA(seurat_object, assay = "integrated", verbose = FALSE,
                        rev.pca = TRUE, reduction.name = "pca.rev",
                        reduction.key="PCR_", npcs = 50)
E <- seurat_object@reductions$pca.rev@feature.loadings
```

```r
pathways <- clusters_split %>% lapply(`[[`, "Gene")
```

```r
gesecaRes <- geseca(pathways = pathways,
                    E = E,
                    minSize = 1,
                    maxSize = 500,
                    center = FALSE,
                    eps=1e-100)
```

Table 3: Network

pathway	pctVar	pval	padj	log2err	size
Cluster_5	0.6691205	0.000000	0.000000	1.5297053	122
Cluster_8	0.2549609	0.000000	0.000000	1.0073180	93
Cluster_1	0.1349124	0.000000	0.000000	0.7477397	103
Cluster_7	0.0298029	0.031968	0.031968	0.2529611	81

All TiCoNE modules are significant based on geseca scores.

Pathways annotation:

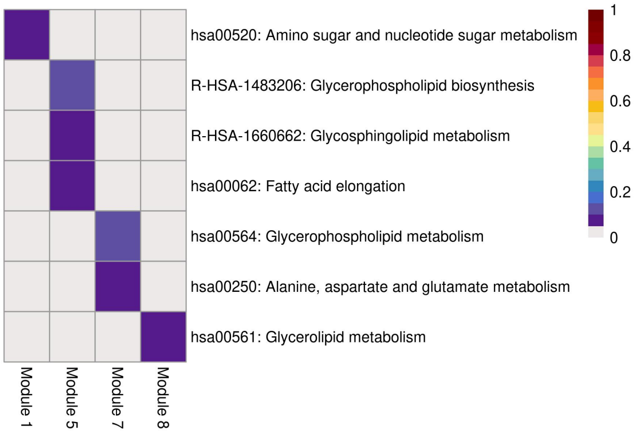

- **Largest connected subnetwork**

```
# filter genes for the largest connected subnetwork
clusters_split <- split(clusters, clusters$Cluster)
for (i in seq_along(clusters_split)) {
  genes_lc <- read.csv(file.path(ticone_clustering,
                                 sprintf("%s_largestComponent.csv",
                                 ↪  names(clusters_split)[i])))$shared.name %>%
    unique()
  clusters_split[[names(clusters_split)[i]]] <-
↪   clusters_split[[names(clusters_split)[i]]] %>%
    filter(Gene %in% genes_lc)
}
clusters_lc <- Reduce(rbind, clusters_split)
```

```
clusters_split <- split(clusters_lc, clusters_lc$Cluster)
geseca_list <- list()
for(i in seq_along(clusters_split)){
  Seurat::DefaultAssay(seurat_object) <- "integrated"
  p <- fgsea::plotCoregulationProfileReduction(clusters_split[[i]]$Gene, seurat_object,
                                               order = T,
                                               reduction = "umap") +
    ggplot2::ggtitle(paste0("Module_", gsub("Cluster_(\\d+)", "\\1",
↪   names(clusters_split)[i])))
  geseca_list[[i]] <- p
}
cowplot::plot_grid(plotlist = geseca_list,
                   ncol = 4)
```

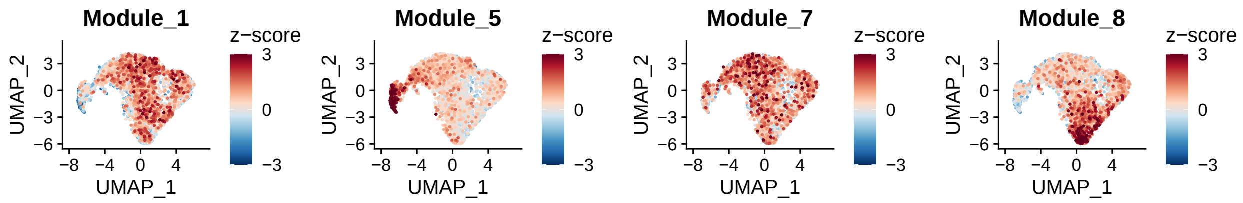


```r
pathways <- clusters_split %>% lapply(`[[`, "Gene")
```

```r
gesecaRes <- geseca(pathways = pathways,
                    E = E,
                    minSize = 1,
                    maxSize = 500,
                    center = FALSE,
                    eps=1e-100)
```

Table 4: Network

pathway	pctVar	pval	padj	log2err	size
Cluster_5	0.4162076	0.0000000	0.0000000	1.2295041	81
Cluster_8	0.1770325	0.0000000	0.0000000	0.8266573	47
Cluster_1	0.1110078	0.0000002	0.0000002	0.6749629	72
Cluster_7	0.0172363	0.2347652	0.2347652	0.0824344	46

TiCoNE module 7 is not significnt based on geseca scores.

Pathways annotation:

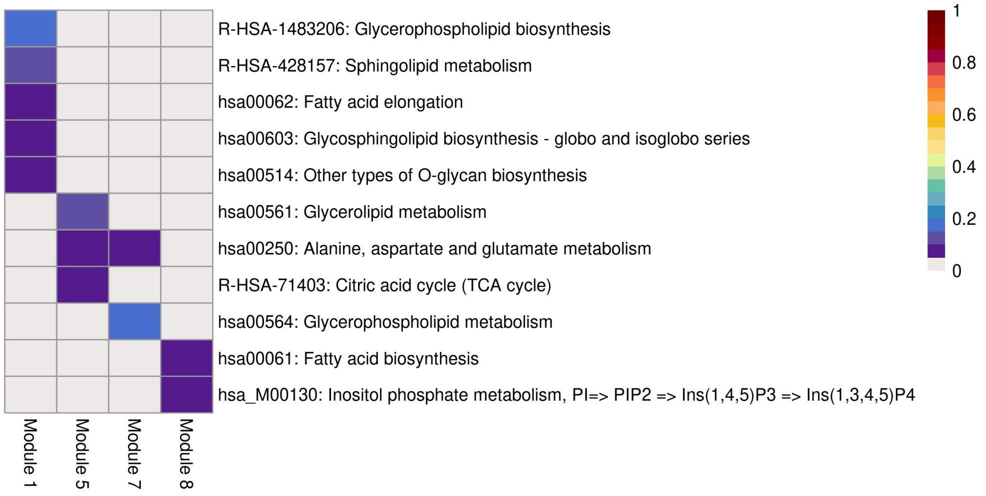

Comparison with the GAM-clustering results

We compared the whole content of TiCoNE and GAM-clustering modules. Only TiCoNE modules 1 and 3 share genes with GAM-clustering module 1.

Upset plot showing intersection of modules genes. TiCoNE_cl.1/cl.5/cl.7/cl.8 denote TiCoNE modules and GAMclust_M1/M2/M3 state for GAM-clustering modules

```
m_files <- list.files(work.dir, "m\\.[0-9]+\\.genes\\.tsv", full.names = T)
gam_mod <- lapply(c(1:length(m_files)), function(i) {
  x <- GAMclust:::read.tsv(m_files[i])$symbol
})
names(gam_mod) <- c("GAMclust_M1", "GAMclust_M2", "GAMclust_M3")
```

Whole TiCoNE modules content

```
plot_lst <- lapply(split(clusters, clusters$Cluster), function(x) x$Gene)
names(plot_lst) <- gsub("Cluster_", "TiCoNE_cl.", names(plot_lst))
plot_lst <- c(plot_lst, gam_mod)
```

```
upset(fromList(plot_lst), sets = rev(names(plot_lst)),
      keep.order = F, order.by = "freq", nintersects = NA,
      point.size = 3.5, line.size = 2,
      mainbar.y.label = "Genre Intersections", sets.x.label = "Modules size")
```

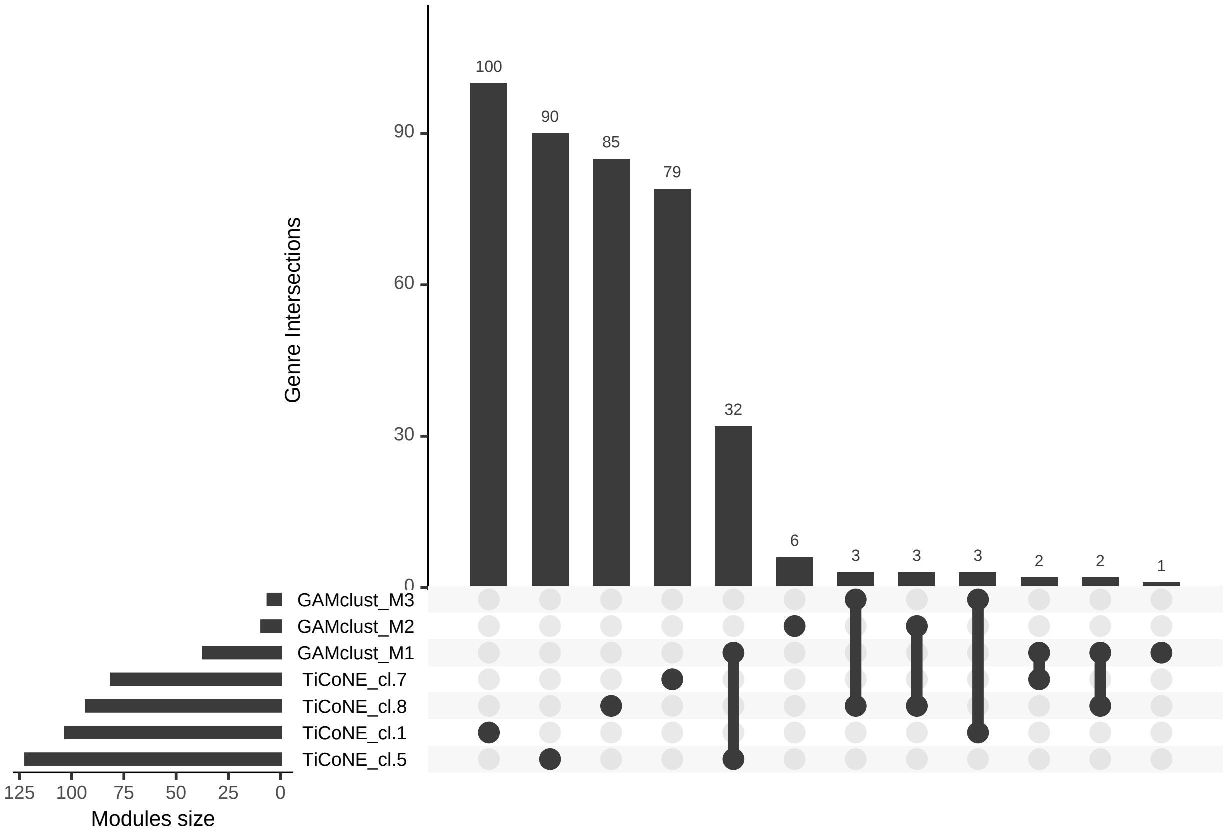

Largest connected component

```
plot_lst <- lapply(split(clusters_lc, clusters_lc$Cluster), function(x) x$Gene)
names(plot_lst) <- gsub("Cluster_", "TiCoNE_cl.", names(plot_lst))
```

```r
plot_lst <- c(plot_lst, gam_mod)
```

```r
upset(fromList(plot_lst), sets = rev(names(plot_lst)),
      keep.order = F, order.by = "freq",
      point.size = 3.5, line.size = 2,
      mainbar.y.label = "Genre Intersections", sets.x.label = "Modules size")
```

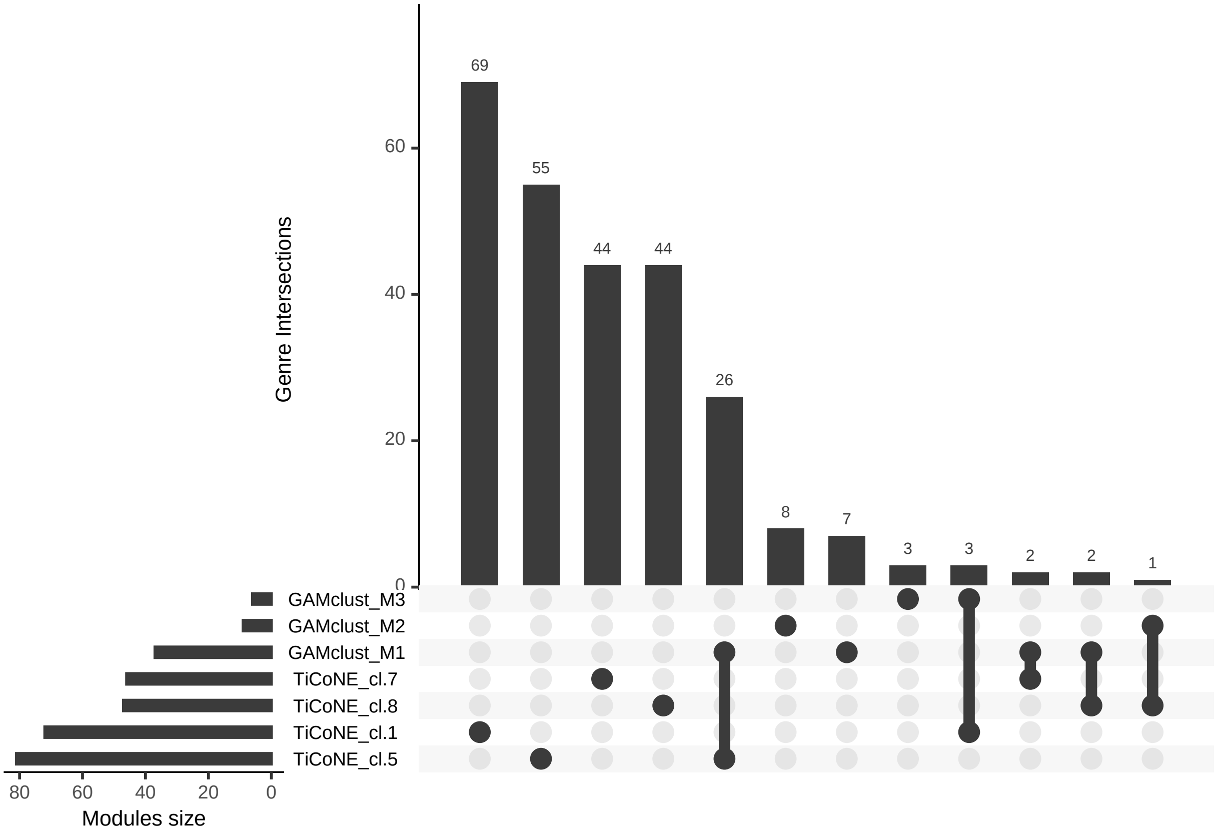

Discussion

TiCoNE algorithm found 3 significant modules:

- Module 1 [geseca: the largest connected subnetwork is not significant]: the largest connected subnetwork is devoted to TCA and shares genes with GAM-clustering module 1 but expression pattern of moules do not match. Other TiCoNE modules genes also devoted to arachidonic acid metabolism.
- Module 3: focused on lipid metabolism (glycerophospholipids, sphingolipids, phosphatidilethanolamine metabolism). No intersection in lipid pathways but overall GAM-clustering module 1 also has a part of sphingosine biosynthesis.
- Module 7 [geseca: not significant]: devoted to cysteine and methionine degradation

TiCoNE was able to capture unique features of the dataset devoted to energy, lipid and amino acid metabolism. Similar to GAM-clustering energy metabolism (TCA) was one of the top findings but the most highlighted changes by TiCoNE were among lipid metabolism.
