# Supplementary material for: GAMclust: identification of regulated metabolic modules in bulk, single cell and spatial gene expression data
Source: Bioinformatics. 2026 Aug 22;42(9):btag639. doi: 10.1093/bioinformatics/btag639 (PMC13544963; doi:10.1093/bioinformatics/btag639)
Supplement: Supplementary file 1 [file btag639_supplementary_data.zip › S8_scCellFie_on_single_cell_RNA-seq_Friedrich_et_al_dataset.pdf]

Supplementary File 8: Metabolic Profiling of Friedrich et al glioma dataset with scCellFie

- Introduction
- Method overview
- Input dataset
- Analysis
- Discussion

Introduction

While various approaches exist to extract metabolic insights from gene expression data, one strategy is to assess the ability of a cell to perform specific metabolic functions, known as tasks. The key advantage compared to FBA-based merthod is the time of analysis. In this vignette, we present a step-by-step application of scCellFie, highlight practical considerations when using it on single-cell datasets, and discuss how its output can be interpreted in the context of metabolic specialization. We further compare its results with GAM-clustering.

Method overview

scCellFie is a computational framework to infer metabolic activities from human and mouse transcriptomic data at single-cell and spatial resolution.

Tool is based on **CellFie** (**Cell**ular **F**unctions **I**nferenc**E**)

Out[2]:

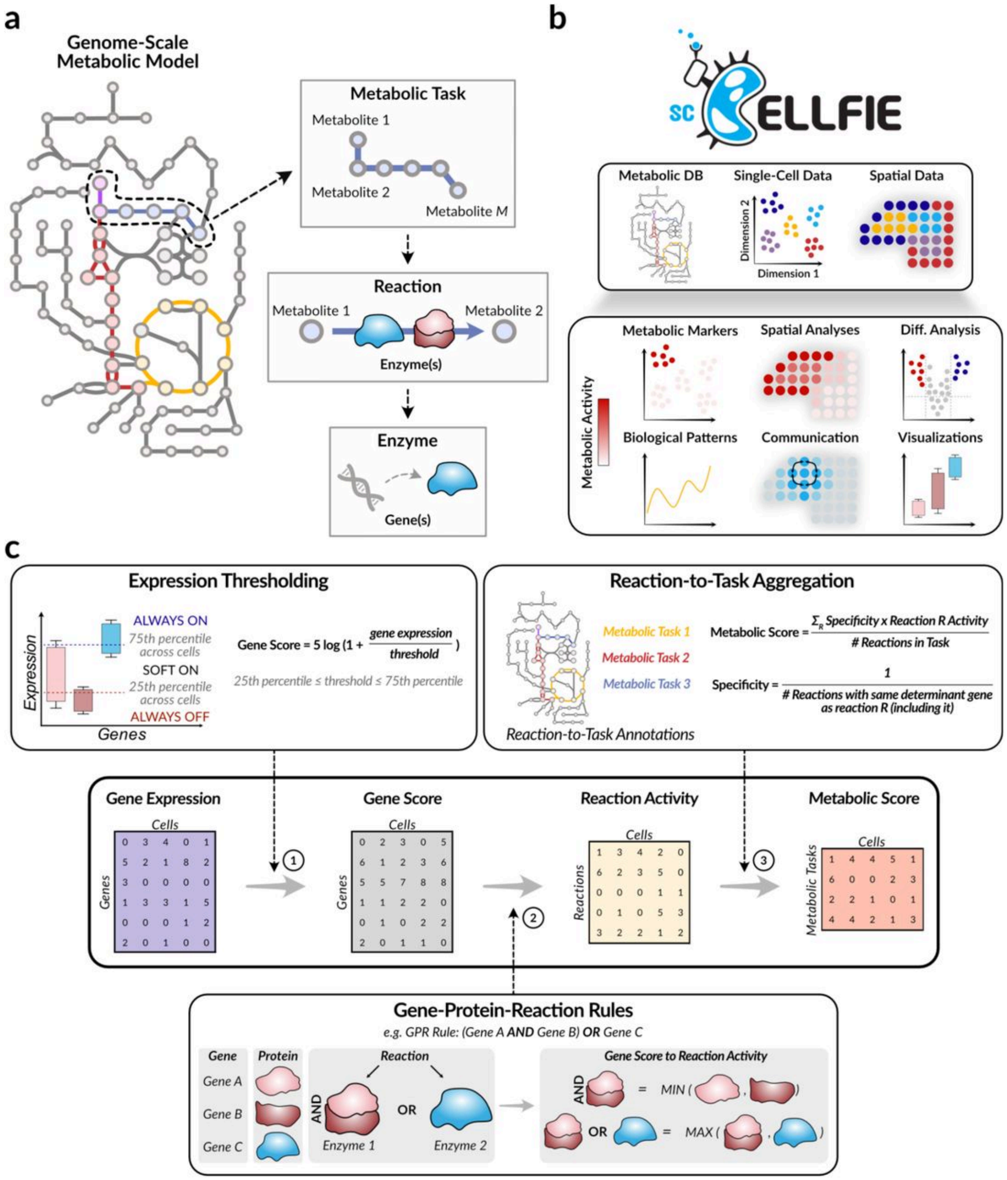

Atlas-scale metabolic activities inferred from single-cell and spatial transcriptomics *Erick Armingol, James Ashcroft, Magda Mareckova, Martin Prete, Valentina Lorenzi, Cecilia Icoresi Mazzeo, Jimmy Tsz Hang Lee, Marie Moullet, Omer Ali Bayraktar, Christian Becker, Krina Zondervan, Luz Garcia-Alonso, Nathan E. Lewis, Roser Vento-Tormo bioRxiv 2025.05.09.653038; doi: https://doi.org/10.1101/2025.05.09.653038*

Model-based assessment of mammalian cell metabolic functionalities using omics data. *Richelle A, Kellman BP, Wenzel AT, Chiang AWT, Reagan T, Gutierrez JM, Joshi C, Li S, Liu JK, Masson H, Lee J, Li Z, Heirendt L, Trefois C, Juarez EF, Bath T, Borland D, Mesirov JP, Robasky K, Lewis NE. Cell Rep Methods. 2021 Jul 26;1(3):100040. doi: 10.1016/j.crmeth.2021.100040. Epub 2021 Jun 30. PMID: 34761247; PMCID: PMC8577426.*

- scCellFie infers metabolic activities in single cells by integrating gene expression data with genome-scale metabolic models
- It uses gene-protein-reaction (GPR) rules to link genes to corresponding enzymes and reactions
- The tool relies on flux balance analysis (FBA) only to identify the essential reactions for each metabolic task
- Metabolic task is a small module of reactions required for generation of specific metabolites given a defined set of substrate metabolites
- To determine whether a gene is active scCellFie uses gene-specific thresholds calculated from large-scale cell atlas data (Chan Zuckerberg CELLxGENE) which accounts for variability across cell types.
- Metabolic task scores are calculated for each metabolic task in each cell
- Final results can be used to compare different conditions, identify distinct metabolic markers and infer cell-cell communiction patterns.

In [3]:
```
## To avoid warnings
import warnings
warnings.filterwarnings("ignore")
```

In [4]:
```
import sccellfie
import scanpy as sc

import pandas as pd
import numpy as np

import matplotlib.pyplot as plt
import seaborn as sns
import glasbey

import textwrap

import pickle
pd.set_option('display.max_colwidth', 100)
```

Input dataset

With scCellFie we are analysing microglial dataset from control and two tumor samples (IDH wild-type and IDH mutant gliomas)

Out[5]:

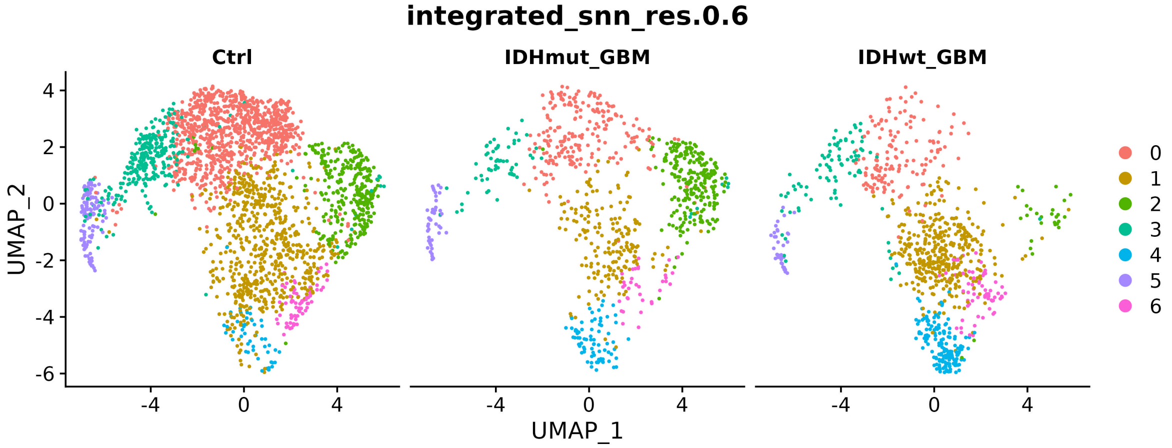

Analysis

Run scCellFie

In [6]:

```
%%time
adata = sc.read(filename='prinz_h_9k.h5ad')

results = sccellfie.run_sccellfie_pipeline(adata,
                                           organism='human',
                                           sccellfie_data_folder=None,
                                           n_counts_col='nCount_RNA',
                                           process_by_group=False,
                                           groupby=None,
                                           n_neighbors=10,
                                           batch_key='Batch',
                                           threshold_key=
                                           'sccellfie_threshold',
                                           smooth_cells=True,
                                           alpha=0.33,
                                           chunk_size=1000,
                                           disable_pbar=False,
                                           save_folder=None,
                                           save_filename=None
                                          )
```

```
==== scCellFie Pipeline: Initializing ====
Loading scCellFie database for organism: human

==== scCellFie Pipeline: Processing entire dataset ====

---- scCellFie Step: Preprocessing data ----

---- scCellFie Step: Computing neighbors ----

---- scCellFie Step: Preparing inputs ----
Gene names corrected to match database: 22
Shape of new adata object: (4153, 810)
Number of GPRs: 740
Shape of tasks by genes: (215, 810)
Shape of reactions by genes: (740, 810)
Shape of tasks by reactions: (215, 740)

---- scCellFie Step: Smoothing gene expression ----
```

```
Smoothing Expression: 100%|
████████████████████████████████████████████████████████████████| 1/1
[00:00<00:00,  3.60it/s]
```

```
---- scCellFie Step: Computing gene scores ----

---- scCellFie Step: Computing reaction activity ----
```

```
Cell Rxn Activities: 100%|
██████████████████████████████████████████████████████████| 4153/4153
[00:14<00:00, 282.49it/s]
```

```
---- scCellFie Step: Computing metabolic task activity ----
Removed 1 metabolic tasks with zeros across all cells.

==== scCellFie Pipeline: Processing completed successfully ====
CPU times: user 1min 48s, sys: 17.5 s, total: 2min 6s
Wall time: 29.6 s
```

Running time

Elapsed Time: **<1 min**

In [7]:

```
adata_save = results['adata']
adata_save.__dict__['_raw'].__dict__['_var'] =
adata_save.__dict__['_raw'].__dict__['_var'].rename(columns={'_index':
'features'})
sccellfie.io.save_adata(adata=adata_save,
                        output_directory='sccellfie_results',
                        filename='prinz_9k_RNA')
with open('sccellfie_results/results_prinz_9k_RNA.pkl', 'wb') as f:
    pickle.dump(results, f)
```

Visualization of results

In [9]:

```
# Column indicating the cell types in the adata.obs dataframe
cell_group = 'Diagnosis'
# We use glasbey to expand the palette into a larger number of colors
```

```
# This is useful when we have many cell types
palette = glasbey.extend_palette('Set2',
                                  palette_size=max([10, results['adata'].
                                      metabolic_tasks.obs[cell_group].
                                      unique().shape[0]]))
```

Metabolic tasks

Aggregate metabolic task scores across conditions

In [10]:
```
agg = sccellfie.expression.aggregation.agg_expression_cells(
    results['adata'].metabolic_tasks,
    groupby=cell_group,
    agg_func='trimean')
agg.iloc[:,1:4]
```

Out[10]:

Task	3'-Phospho-5'-adenylyl sulfate synthesis	AMP salvage from adenine	ATP generation from glucose (hypoxic conditions) - glycolysis
**Ctrl**	0.060296	0.106277	1.162983
**IDHmut_GBM**	0.090679	0.092157	1.378289
**IDHwt_GBM**	0.070477	0.126614	1.409273

Visualize activity of metabolic tasks

Metabolik tasks scores can only be compared between different cells. Here we use proposed workflow normalizing activity scores so that 0 is assigned to the group with the lowest value and 1 - with the highest. Normalized values reflect the magnitude of changes between conditions.

In [11]:
```
input_df = sccellfie.preprocessing.matrix_utils.min_max_normalization(
    agg.T, axis=1)
input_df.head()
```

Out[11]:

Task	Ctrl	IDHmut_GBM	IDHwt_GBM
**(R)-3-Hydroxybutanoate synthesis**	0.000000	0.988552	1.000000
**3'-Phospho-5'-adenylyl sulfate synthesis**	0.000000	1.000000	0.335088
**AMP salvage from adenine**	0.409773	0.000000	1.000000
**ATP generation from glucose (hypoxic conditions) - glycolysis**	0.000000	0.874199	1.000000
**ATP regeneration from glucose (normoxic conditions) - glycolysis + krebs cycle**	0.000000	0.740231	1.000000

In [12]:

```
df_melted = pd.melt(input_df.reset_index(),
                    id_vars='Task',
                    var_name='cell_type',
                    value_name='scaled_trimean')
df_melted = df_melted.rename(columns={'Task': 'metabolic_task'})
df_melted.head()
```

Out[12]:

	metabolic_task	cell_type	scaled_trimean
**0**	(R)-3-Hydroxybutanoate synthesis	Ctrl	0.000000
**1**	3'-Phospho-5'-adenylyl sulfate synthesis	Ctrl	0.000000
**2**	AMP salvage from adenine	Ctrl	0.409773
**3**	ATP generation from glucose (hypoxic conditions) - glycolysis	Ctrl	0.000000
**4**	ATP regeneration from glucose (normoxic conditions) - glycolysis + krebs cycle	Ctrl	0.000000

```
In [13]: cell_types = results['adata'].obs.Diagnosis.unique().tolist()
```

```
In [14]: # Create figure with subplots
fig = plt.figure(figsize=(20, 10))
ax1 = fig.add_subplot(131, projection='polar')
ax2 = fig.add_subplot(132, projection='polar')
ax3 = fig.add_subplot(133, projection='polar')
for i, (cell, ax) in enumerate(zip(cell_types, [ax1, ax2, ax3])):
    sccellfie.plotting.create_radial_plot(df_melted,
                                          results['task_info'],
                                          cell_type=cell,
                                          ax=ax,
                                          show_legend=i == 2,
                                          ylim=1.0
                                          )
```

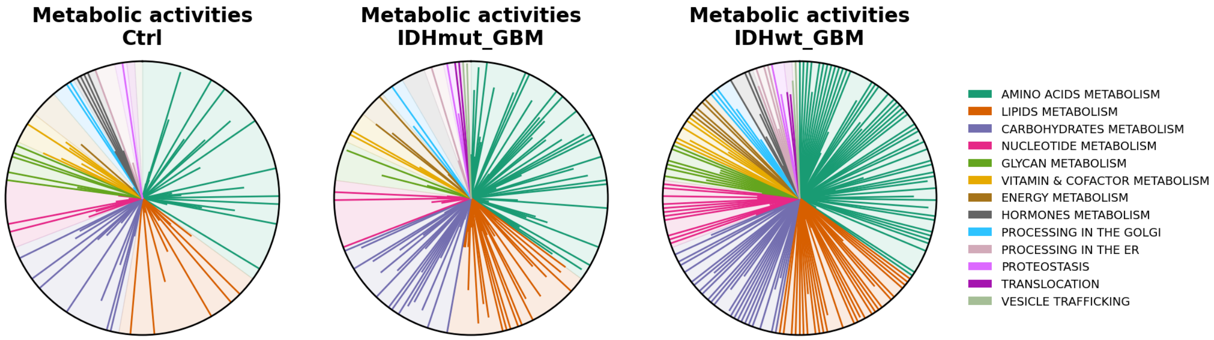


```
In [15]: # Create figure with subplots
fig = plt.figure(figsize=(20, 10))
ax1 = fig.add_subplot(131, projection='polar')
ax2 = fig.add_subplot(132, projection='polar')
ax3 = fig.add_subplot(133, projection='polar')

for i, (cell, ax) in enumerate(zip(cell_types, [ax1, ax2, ax3])):
    sccellfie.plotting.create_radial_plot(df_melted,
                                          results['task_info'],
                                          cell_type=cell,
                                          ax=ax,
                                          show_legend=i == 2,
                                          ylim=1.0,
                                          sort_by_value=True
                                          )
```

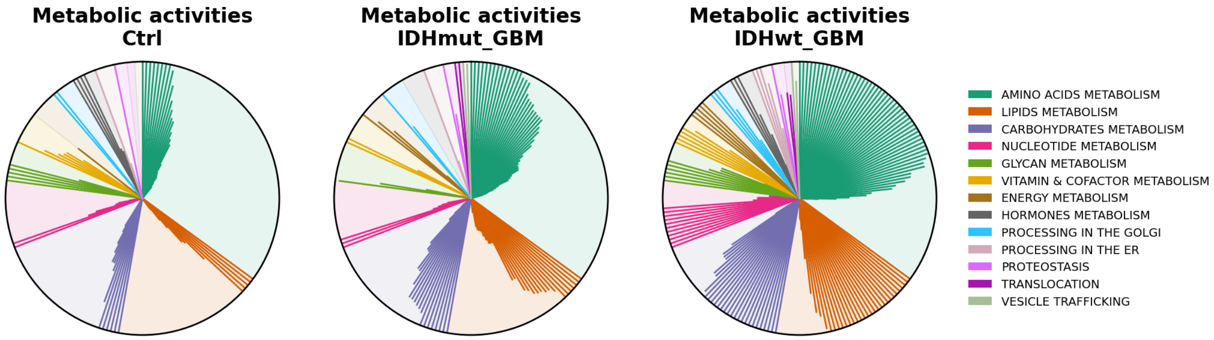

We can see that in control sample most of the metabolic tasks have the lowest activity. IDHwt tumor samples are the most metabolicaly active among all three groups. IDHmut samples are in the middle in the terms of metabolic activity supporting evidence of the better prognosis of IDHmut tumors.

Metabolic markers

For each predefined cell group scCellFie can find disctinct metabolic tasks specific to this cluster.

Detection using TF-IDF

In [16]:
```
mrks = sccellfie.external.quick_markers(results['adata'].metabolic_tasks,
                                        cluster_key=cell_group,
                                        n_markers=20,
                                        express_cut=5*np.log(2))
```

In [17]:
```
plt.figure(figsize=(3,2))
mrks['tf_idf'].hist()
```

Out[17]: <Axes: >

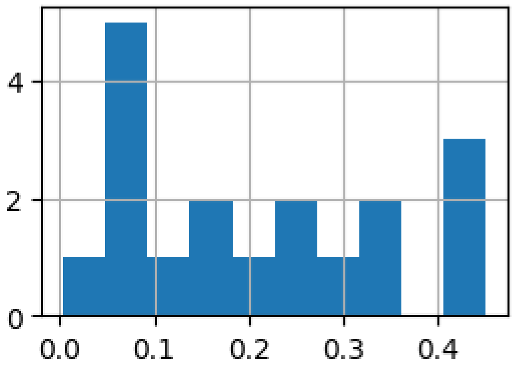

In [18]:
```
plt.figure(figsize=(4,3))
scatter = plt.scatter(mrks['tf'], mrks['idf'],
                      c=mrks['tf_idf'], cmap='YlGnBu')
plt.colorbar(scatter)
plt.xlabel('TF')
plt.ylabel('IDF')
```

Out[18]: Text(0, 0.5, 'IDF')

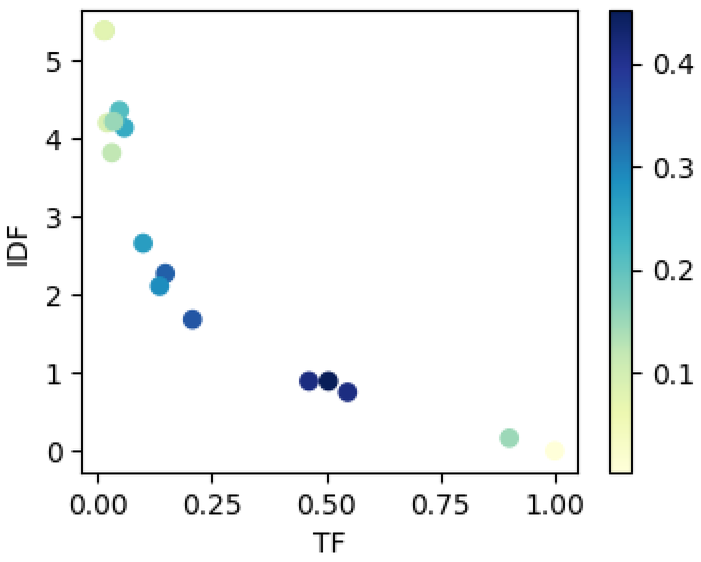

Select 0.2 threshold based on the histogram

In [19]:
```
x_col = 'tf'
y_col = 'idf'
df = mrks
tfidf_threshold = 0.2
tf_ratio = 1.2

# Visualization
plt.figure(figsize=(4,3))
# Plot all points
plt.scatter(df[x_col], df[y_col], alpha=0.5, c='lightgray', label='All
points')
# Plot selected points
filtered_mrks, curve = sccellfie.external.filter_tfidf_markers(
    df, tf_col=x_col, idf_col=y_col,
    tfidf_threshold=tfidf_threshold,
    tf_ratio=tf_ratio)
plt.scatter(filtered_mrks[x_col], filtered_mrks[y_col],
            c='red', label='Selected points')
plt.plot(*curve, label='Fit curve')
plt.xlabel('TF')
plt.ylabel('IDF')
plt.legend()
```

Out[19]:
```
<matplotlib.legend.Legend at 0x7fca0a127f40>
```

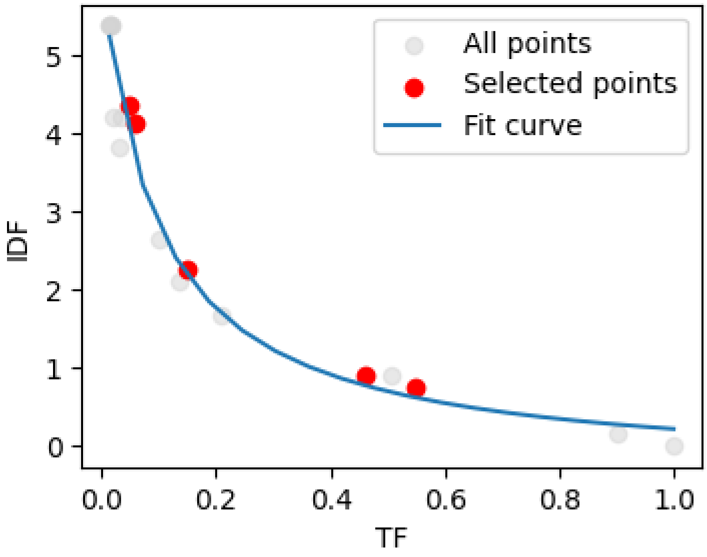

In [20]:
```
# Get unique list of filtered markers
tf_idf_mrks = filtered_mrks['gene'].unique().tolist()
tf_idf_mrks
```

Out[20]:
```
['Conversion of 1-phosphatidyl-1D-myo-inositol 4,5-bisphosphate to 1D-myo-
inositol 1,4,5-trisphosphate',
 'Synthesis of androstenedione from cholesterol',
 'Conversion of glutamate to glutamine',
 'ATP generation from glucose (hypoxic conditions) - glycolysis',
 'Glucose to lactate conversion']
```

Detection using Logistic Regression in Scanpy

In [21]:
```
method = 'logreg'
sc.tl.rank_genes_groups(results['adata'].metabolic_tasks,
                        cell_group, method=method,
                       use_raw=False, key_added=method)
# Get the results as a dataframe
scanpy_df = sc.get.rank_genes_groups_df(results['adata'].
    metabolic_tasks, key=method, group=None)
```

In [22]:
```
# Visualize score distribution
plt.figure(figsize=(3,2))
scanpy_df['scores'].hist()
```

Out[22]:
```
<Axes: >
```

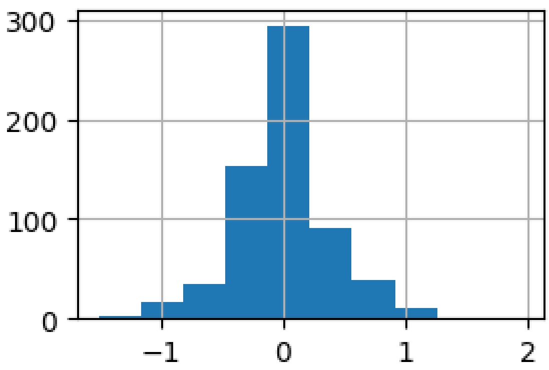

In [23]:
```
# Filter based on score threshold
scores_threshold = 0.5
filtered_scanpy_mrks = scanpy_df.loc[scanpy_df['scores'] >
    scores_threshold]

# Convert them to a list
scanpy_mrks = filtered_scanpy_mrks['names'].unique().tolist()
```

Visualize aggregated results

In [24]:
```
both_markers = sorted(set(tf_idf_mrks + scanpy_mrks))
```

In [25]:
```
# Select only highly active markers
# Aggregate the metabolic activity of markers into
# a cell-type level using trimean
agg = sccellfie.expression.aggregation.agg_expression_cells(
    results['adata'].metabolic_tasks[:, both_markers],
    groupby=cell_group,
    agg_func='trimean')
both_markers = agg.T.loc[(agg.T > 0.5).any(axis=1)].index.tolist()

filtered_agg = agg[both_markers]
normalized_markers = sccellfie.preprocessing.min_max_normalization(
    filtered_agg, axis=0)
```

In [26]:
```
# Create heatmap
plt.figure(figsize=(18, 10))
sns.heatmap(normalized_markers, cmap='YlOrBr', annot=False, linewidths=0.5,
            cbar_kws={'label': 'Min-max scaled metabolic score'})

plt.title('Metabolic Markers across Cell Types')
plt.ylabel('Cell Type')
plt.xlabel('Metabolic Task')
plt.yticks(rotation=0)
plt.tight_layout()
```

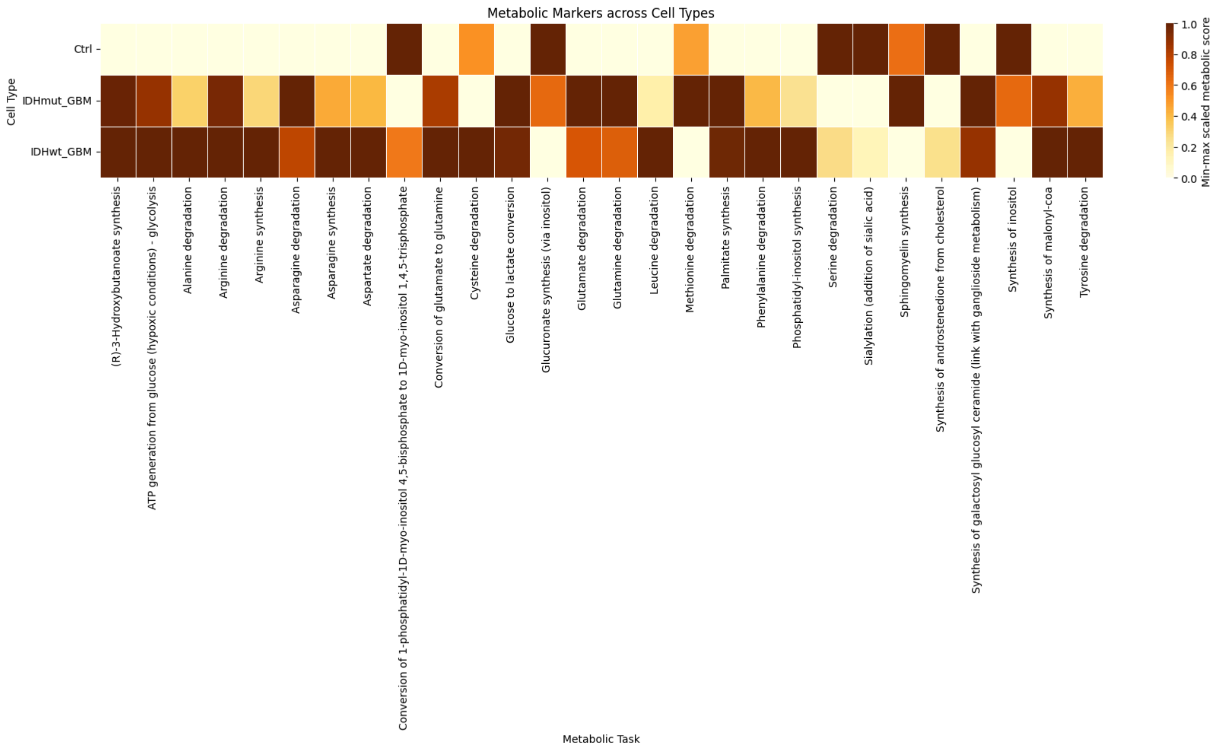

scCellFie identified as control-specific markers pathways related to inositol phosphate synthesis, arginine, cholesterol and sphingomyelin methabolism and synthesis of malonyl-CoA. IDHmut tumor samples show more energy metabolism related tasks (pyruvate), amino acids degradation, glutamate and glutamine methabolism. IDHwt samples exhibit the highest production of ATP and wide range of amino acids related tasks. Except increased glycolysis we see downregulation of glucuronate production (important for detoxification) from control to IDHwt samples which is consistent with the prognosis of both tumosrs.

In [27]:
```
filtered_markers = normalized_markers.columns.tolist()
len(filtered_markers)
```

Out[27]: 28

Intersection of metabolic markers genes with GAM-clustering modules

In [28]:
```
m1 = set(pd.read_csv('m.1.genes.tsv',
                     sep='\t')['symbol'].dropna())
m2 = set(pd.read_csv('m.2.genes.tsv',
                     sep='\t')['symbol'].dropna())
m3 = set(pd.read_csv('m.3.genes.tsv',
                     sep='\t')['symbol'].dropna())
```

In [29]:
```
df = results['task_by_gene'].loc[df_melted.
    metabolic_task.unique().tolist()]
df = df.filter(items=filtered_markers, axis=0)
task_gene_intersections = {
```

```python
    task: {
        'm1': list(m1.intersection(df.columns[df.loc[task] == 1])),
        'm2': list(m2.intersection(df.columns[df.loc[task] == 1])),
        'm3': list(m3.intersection(df.columns[df.loc[task] == 1]))
    }
    for task in df.index
}
df_result = pd.DataFrame.from_dict(task_gene_intersections, orient='index')
df_result = df_result[
    df_result['m1'].astype(bool) | df_result['m2'].astype(bool) |
df_result['m3'].astype(bool)
]

df_result['m1_count'] = df_result['m1'].apply(len)
df_result['m2_count'] = df_result['m2'].apply(len)
df_result['m3_count'] = df_result['m3'].apply(len)
```

GAM-clustering module 1

In [30]:
```python
df_m1_sorted = df_result[df_result['m1'] != ''].sort_values(
    by='m1_count', ascending=False)
# Drop zero-count rows
df_m1_sorted = df_m1_sorted[df_m1_sorted['m1_count'] > 0]
# Aggregate list of genes into a string
df_m1_sorted['m1'] = df_m1_sorted['m1'].apply(lambda genes: ', '.
    join(genes) if isinstance(genes, list) else genes)
# Reset index and keep only relevant columns
df_m1_sorted = df_m1_sorted.reset_index().rename(columns={
    'index': 'metabolic_task'})
df_m1_sorted = df_m1_sorted[['metabolic_task', 'm1']]
df_m1_sorted.head(15)
```

Out[30]:

	metabolic_task	m1
**0**	Synthesis of galactosyl glucosyl ceramide (link with ganglioside metabolism)	SDHC, KDSR, CPT1A, SLC16A7, ELOVL6, SPTLC1, OGDH, ACSL6
**1**	Sphingomyelin synthesis	KDSR, CPT1A, SLC16A7, ELOVL6, SPTLC1, ACSL6, DGKH
**2**	Phosphatidyl-inositol synthesis	SDHC, CPT1A, SLC16A7, ELOVL6, OGDH, ACSL6
**3**	Aspartate degradation	IDH2, SDHC, SLC16A7, GOT1, OGDH
**4**	Alanine degradation	IDH2, SDHC, SLC16A7, GOT1, OGDH
**5**	Asparagine degradation	IDH2, SDHC, SLC16A7, GOT1, OGDH
**6**	Arginine degradation	SDHC, GOT1, OAT, ALDH5A1, OGDH
**7**	Tyrosine degradation	IDH2, SDHC, SLC16A7, GOT1, OGDH
**8**	Leucine degradation	IDH2, SDHC, SLC16A7, GOT1, OGDH
**9**	Glutamine degradation	IDH2, SDHC, SLC16A7, GOT1, OGDH
**10**	Glutamate degradation	IDH2, SDHC, SLC16A7, GOT1, OGDH
**11**	Phenylalanine degradation	IDH2, SDHC, SLC16A7, GOT1, OGDH
**12**	Cysteine degradation	IDH2, GOT1, SLC16A7
**13**	Methionine degradation	ALDH2, SDHC, OGDH
**14**	Asparagine synthesis	SDHC, GOT1, OGDH

In [31]:

```
reactions_f = df_m1_sorted.metabolic_task.tolist()[:5]

plt.rcParams['figure.figsize'] = (3,3)
plt.rcParams['font.size'] = 10

sc.pl.embedding(results['adata'].metabolic_tasks,
                color=[cell_group] + reactions_f,
                ncols=3,
                palette=palette,
                frameon=False,
                basis='X_umap',
                wspace=0.7,
                title=["\n".join(textwrap.wrap(
                    t, width=60)) for t in [cell_group] + reactions_f],
```

```
                  cmap='OrRd'
                 )
```

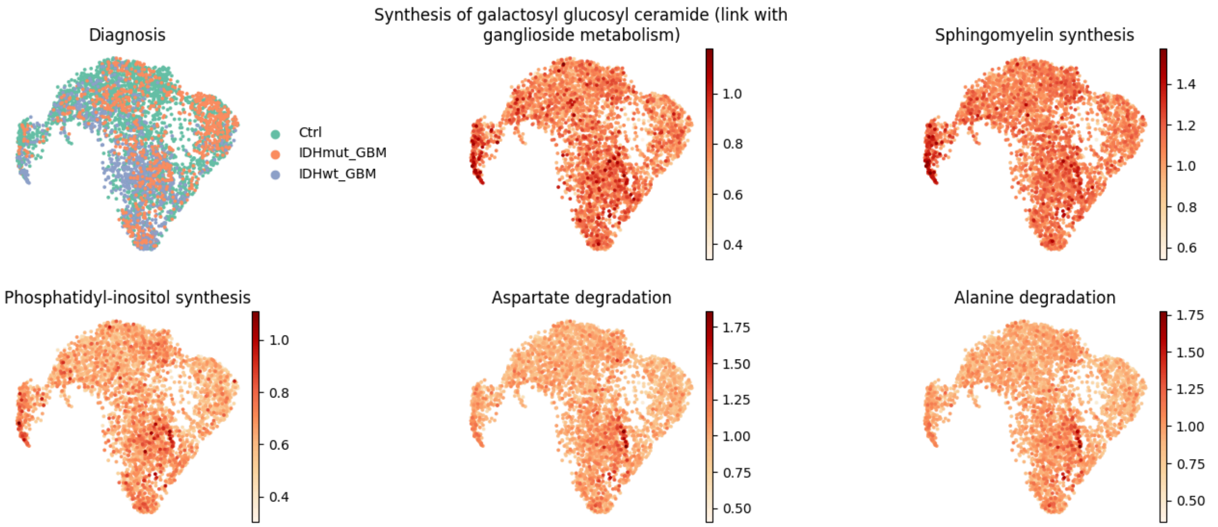

GAM-clustering module 2

In [32]:
```
df_m2_sorted = df_result[df_result['m2'] != ''].sort_values(
    by='m2_count', ascending=False)
# Drop zero-count rows
df_m2_sorted = df_m2_sorted[df_m2_sorted['m2_count'] > 0]
# Aggregate list of genes into a string
df_m2_sorted['m2'] = df_m2_sorted['m2'].apply(lambda genes: ', '.
    join(genes) if isinstance(genes, list) else genes)
# Reset index and keep only relevant columns
df_m2_sorted = df_m2_sorted.reset_index().rename(columns={
    'index': 'metabolic_task'})
df_m2_sorted = df_m2_sorted[['metabolic_task', 'm2']]
df_m2_sorted.head(15)
```

Out[32]:

	metabolic_task	m2
**0**	Alanine degradation	ALDOA, PGK1, LDHA, TPI1, PGAM1, GAPDH, ENO1
**1**	Arginine synthesis	ALDOA, PGK1, PKM, TPI1, PGAM1, GAPDH, ENO1
**2**	Arginine degradation	ALDOA, PGK1, PKM, TPI1, PGAM1, GAPDH, ENO1
**3**	Palmitate synthesis	ALDOA, PGK1, LDHA, TPI1, PGAM1, GAPDH, ENO1
**4**	Leucine degradation	ALDOA, PGK1, LDHA, TPI1, PGAM1, GAPDH, ENO1
**5**	Glucose to lactate conversion	ALDOA, PGK1, LDHA, TPI1, PGAM1, GAPDH, ENO1
**6**	Aspartate degradation	ALDOA, PGK1, LDHA, TPI1, PGAM1, GAPDH, ENO1
**7**	Asparagine synthesis	ALDOA, PGK1, TPI1, PGAM1, GAPDH, ENO1
**8**	Asparagine degradation	ALDOA, PGK1, TPI1, PGAM1, GAPDH, ENO1
**9**	(R)-3-Hydroxybutanoate synthesis	ALDOA, PGK1, TPI1, PGAM1, GAPDH, ENO1
**10**	ATP generation from glucose (hypoxic conditions) - glycolysis	ALDOA, PGK1, TPI1, PGAM1, GAPDH, ENO1
**11**	Glutamine degradation	ALDOA, PGK1, TPI1, PGAM1, GAPDH, ENO1
**12**	Synthesis of malonyl-coa	ALDOA, PGK1, TPI1, PGAM1, GAPDH, ENO1
**13**	Phenylalanine degradation	ALDOA, PGK1, TPI1, PGAM1, GAPDH, ENO1
**14**	Glutamate degradation	ALDOA, PGK1, TPI1, PGAM1, GAPDH, ENO1

In [33]:

```
reactions_f = df_m2_sorted.metabolic_task.tolist()[:6] + [
    'ATP generation from glucose (hypoxic conditions) - glycolysis']
plt.rcParams['figure.figsize'] = (3,3)
plt.rcParams['font.size'] = 10

sc.pl.embedding(results['adata'].metabolic_tasks,
                color=[cell_group] + reactions_f,
                ncols=4,
                palette=palette,
                frameon=False,
                basis='X_umap',
                wspace=0.7,
                title=["\n".join(textwrap.wrap(
                    t, width=60)) for t in [cell_group] + reactions_f],
```

```
                 cmap='OrRd'
                )
```

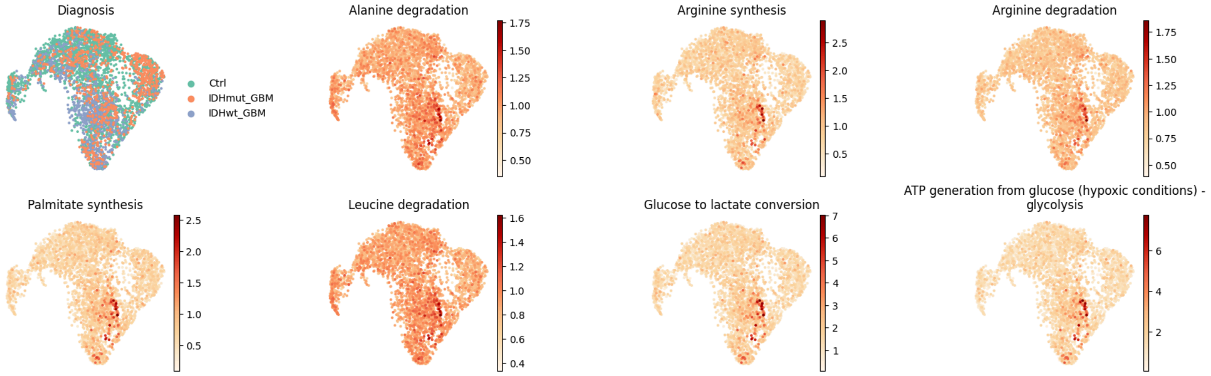

GAM-clustering module 3

In [34]:

```
df_m3_sorted = df_result[df_result['m3'] != ''].sort_values(
    by='m3_count', ascending=False)
# Drop zero-count rows
df_m3_sorted = df_m3_sorted[df_m3_sorted['m3_count'] > 0]
# Aggregate list of genes into a string
df_m3_sorted['m3'] = df_m3_sorted['m3'].apply(lambda genes: ', '.
    join(genes) if isinstance(genes, list) else genes)
# Reset index and keep only relevant columns
df_m3_sorted = df_m3_sorted.reset_index().rename(columns={
    'index': 'metabolic_task'})
df_m3_sorted = df_m3_sorted[['metabolic_task', 'm3']]
df_m3_sorted.head(15)
```

Out[34]:

	metabolic_task	m3
**0**	Synthesis of galactosyl glucosyl ceramide (link with ganglioside metabolism)	UGCG
**1**	Palmitate synthesis	LIPA

In [35]:

```
reactions_f = df_m3_sorted.metabolic_task.tolist()[:6]
plt.rcParams['font.size'] = 10

sc.pl.embedding(results['adata'].metabolic_tasks,
                 color=[cell_group] + reactions_f,
                 ncols=4,
                 palette=palette,
                 frameon=False,
                 basis='X_umap',
                 wspace=0.7,
                 title=["\n".join(textwrap.wrap(
                     t, width=60)) for t in [cell_group] + reactions_f],
                 cmap='OrRd'
                )
```

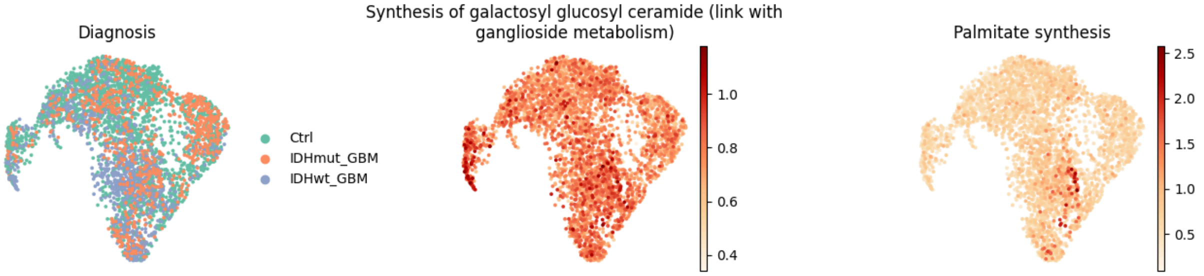

Save results

In [36]:

```python
# Add row order to preserve sorting
df_m1_sorted['__order'] = range(len(df_m1_sorted))
df_m1_scored = df_m1_sorted.merge(df_melted, on='metabolic_task',
how='left')
df_m1_wide = df_m1_scored.pivot(index='metabolic_task',
columns='cell_type',
                                values='scaled_trimean').reset_index()
df_m1_wide = df_m1_sorted[['metabolic_task', 'm1', '__order']].merge(
    df_m1_wide, on='metabolic_task', how='left')
df_m1_wide = df_m1_wide.sort_values('__order').drop(
    columns='__order').reset_index(drop=True)
df_m1_wide.head()
```

Out[36]:

	metabolic_task	m1	Ctrl	IDHmut_GBM	IDHwt_GBM
**0**	Synthesis of galactosyl glucosyl ceramide (link with ganglioside metabolism)	SDHC, KDSR, CPT1A, SLC16A7, ELOVL6, SPTLC1, OGDH, ACSL6	0.000000	1.000000	0.873262
**1**	Sphingomyelin synthesis	KDSR, CPT1A, SLC16A7, ELOVL6, SPTLC1, ACSL6, DGKH	0.627929	1.000000	0.000000
**2**	Phosphatidyl-inositol synthesis	SDHC, CPT1A, SLC16A7, ELOVL6, OGDH, ACSL6	0.000000	0.248623	1.000000
**3**	Aspartate degradation	IDH2, SDHC, SLC16A7, GOT1, OGDH	0.000000	0.409877	1.000000
**4**	Alanine degradation	IDH2, SDHC, SLC16A7, GOT1, OGDH	0.000000	0.317990	1.000000

In [37]:

```
df_m2_sorted['__order'] = range(len(df_m2_sorted))
df_m2_scored = df_m2_sorted.merge(df_melted, on='metabolic_task',
                                  how='left')
df_m2_wide = df_m2_scored.pivot(index='metabolic_task',
columns='cell_type',
                                values='scaled_trimean').reset_index()
df_m2_wide = df_m2_sorted[['metabolic_task', 'm2', '__order']].merge(
    df_m2_wide, on='metabolic_task', how='left')
df_m2_wide = df_m2_wide.sort_values('__order').drop(
    columns='__order').reset_index(drop=True)
df_m2_wide.head()
```

Out[37]:

	metabolic_task	m2	Ctrl	IDHmut_GBM	IDHwt_GBM
**0**	Alanine degradation	ALDOA, PGK1, LDHA, TPI1, PGAM1, GAPDH, ENO1	0.0	0.317990	1.000000
**1**	Arginine synthesis	ALDOA, PGK1, PKM, TPI1, PGAM1, GAPDH, ENO1	0.0	0.298430	1.000000
**2**	Arginine degradation	ALDOA, PGK1, PKM, TPI1, PGAM1, GAPDH, ENO1	0.0	0.951640	1.000000
**3**	Palmitate synthesis	ALDOA, PGK1, LDHA, TPI1, PGAM1, GAPDH, ENO1	0.0	1.000000	0.971298
**4**	Leucine degradation	ALDOA, PGK1, LDHA, TPI1, PGAM1, GAPDH, ENO1	0.0	0.167589	1.000000

In [39]:

```
df_m3_sorted['__order'] = range(len(df_m3_sorted))
df_m3_scored = df_m3_sorted.merge(df_melted, on='metabolic_task',
                                    how='left')
df_m3_wide = df_m3_scored.pivot(index='metabolic_task',
columns='cell_type',
                                values='scaled_trimean').reset_index()
df_m3_wide = df_m3_sorted[['metabolic_task', 'm3', '__order']].merge(
    df_m3_wide, on='metabolic_task', how='left')
df_m3_wide = df_m3_wide.sort_values('__order').drop(
    columns='__order').reset_index(drop=True)
df_m3_wide.head()
```

Out[39]:

	metabolic_task	m3	Ctrl	IDHmut_GBM	IDHwt_GBM
**0**	Synthesis of galactosyl glucosyl ceramide (link with ganglioside metabolism)	UGCG	0.0	1.0	0.873262
**1**	Palmitate synthesis	LIPA	0.0	1.0	0.971298

Discussion

However, results of GAM-clustering do not neccesserly should reflect condition differences, we can see that pathways found by GAM-clustering were also highlighted by scCellFie as changing from condition to condition. Glycolysis was found by both tools as pathway specific to GBM samples with the highest activity in IDHwt samples. Both methods were able to capture importance of lipid methabolism. scCellFie also identified a wide range of condition-specific metabolic tasks including tasks related to metabolism of amino acids, lipids, inositol phosphate and glucuronic acid.
