# Supplementary material for: GAMclust: identification of regulated metabolic modules in bulk, single cell and spatial gene expression data
Source: Bioinformatics. 2026 Aug 22;42(9):btag639. doi: 10.1093/bioinformatics/btag639 (PMC13544963; doi:10.1093/bioinformatics/btag639)
Supplement: Supplementary file 1 [file btag639_supplementary_data.zip › S9_Compass_on_single_cell_RNA-seq_Friedrich_et_al_dataset.pdf]

Supplementary File 9: Metabolic Profiling of Friedrich et al glioma dataset with Compass

- Introduction
- Method overview
- Input dataset
- Analysis
    - Comparison of glioma IDHwt and IDHmut samples
    - Comparison of control and glioma samples
    - Comparison of control and IDHwt glioma samples
    - Comparison of control and IDHmut glioma samples
- Discussion
- Helper functions

Introduction

One powerful approach to investigate metabolic changes using transcriptomic data involves the use of flux balance analysis (FBA) — a constraint-based modeling technique that estimates the flow of metabolites through a metabolic network. While traditional FBA applications have focused on bulk transcriptomic, Compass was specifically designed to infer metabolic states from scRNA-seq data.

Our goal is to provide a detailed and reproducible workflow for Compass analysis and compare its results with GAM-clustering modules.

Method overview

Compass is an algorithm to characterize the metabolic state of cells based on single-cell RNA-Seq and flux balance analysis (FBA).

Out[1]:

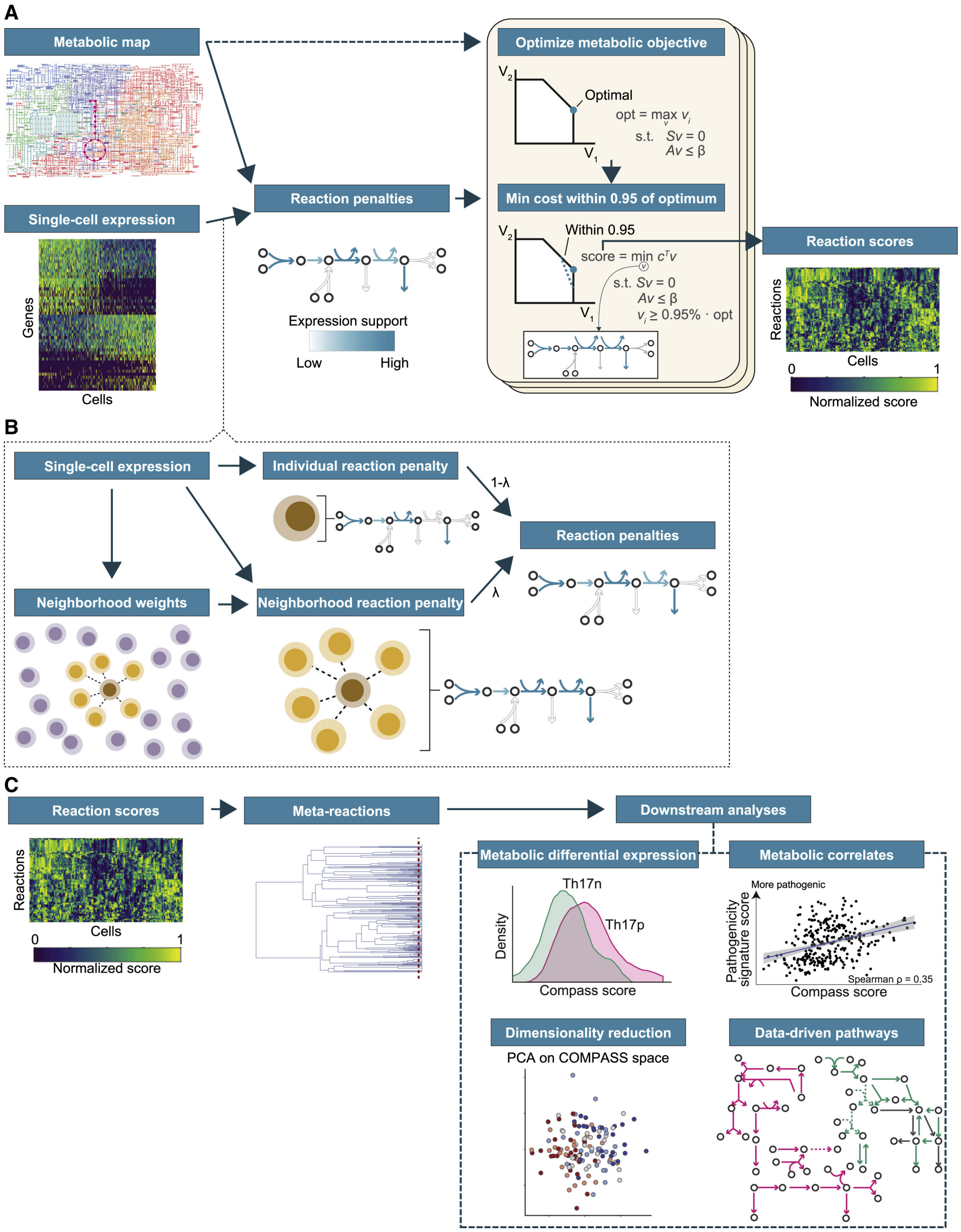

Wagner A, Wang C, Fessler J, et al. Metabolic modeling of single Th17 cells reveals regulators of autoimmunity. Cell. 2021;184(16):4168-4185.e21. doi:10.1016/j.cell.2021.05.045

https://www.cell.com/cell/fulltext/S0092-8674(21)00700-5

- Compass uses a genome-scale metabolic network to model thousands of metabolic reactions
- Activity of reactions are based on expression of enzymes allowing to map transcriptomic data to metabolic network
- Based on gene expression it estimates feasibility of metabolic reactions in each cell by solving a flux balance optimisation problem for each reaction
- Method evaluates flux activity at the single-cell resolution allowing to quantify the metabolic potential of single cells downt to individual reactions

In [28]:
```
import warnings
warnings.filterwarnings("ignore")
```

Input dataset

Dataset is composed of microglia from control and two tumor samples (IDH wild-type and mutant)

Out[2]:

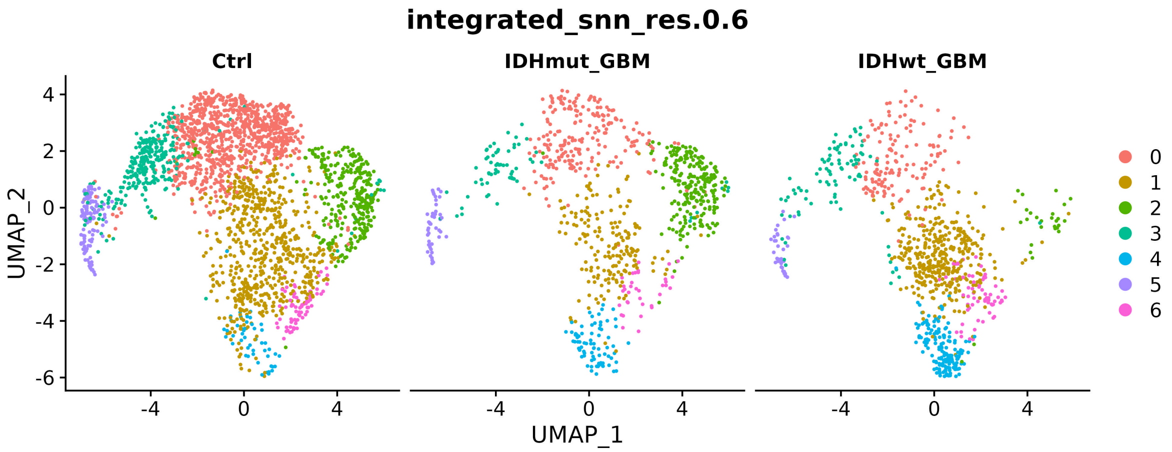

As input data for Compass we used RC RNA normalized data as recommended by authors. All the initial preprocessing steps were performed in R, final count matrix was saved and used inthis analysis.

In [3]:
```
# in R
seurat_object <- NormalizeData(seurat_object,
                               normalization.method = "RC",
                               scale.factor = 1e+06)
mtx <- GetAssayData(seurat_object, assay = "RNA", slot = "data") %>%
  as.data.frame() %>%
  tibble::rownames_to_column("Gene_Symbol")
```

Analysis

Run Compass

We used CPLEX based version (cplex 22.10) and python 3.7 for the analysis.

Compass was installed from github repository and launched in terminal. Here we provide commands to run compass.

In [4]:
```
# in Terminal:

python -m pip install git+https://github.com/yoseflab/Compass.git

# # Build cache
time compass --precache --species homo_sapiens --num-processes 64

# # Run Compass
compass --data /data/prinz_RNAnormRC_CPM_reint9k.tsv \
            --num-processes 64 \
            --species homo_sapiens
```

Running time

Processing 4153 samples using 128 processes

Elapsed Time: **8h 26min**

+ build cache 58 sec

Postprocessing

Processing results of Compass run we followed pipeline proposed by the authors.

Prepare functions and data

We need to download all the additional scripts and data required to run the analysis. To focuse on the main analysis all functions used in this pipeline are provided in the end in the section Helper functions

In [3]:
```
!wget
https://raw.githubusercontent.com/YosefLab/Compass/refs/heads/compass_v2/do
cs/notebooks/compass_analysis.py
!wget https://raw.githubusercontent.com/wagnerlab-
berkeley/Compass/refs/heads/master/docs/notebooks/extdata/RECON2/reaction_m
etadata.csv
```

In [4]:
```
def install_reqs():
  !pip install pandas
  !pip install "matplotlib>=3.4"
  !pip install numpy
  !pip install statsmodels
```

```
  !pip install scipy
install_reqs()
```

In [3]:
```
import pandas as pd
import numpy as np
import matplotlib.pyplot as plt
from matplotlib_venn import venn2
from collections import defaultdict
from upsetplot import plot, UpSet
```

In [4]:
```
from compass_analysis import (cohens_d, wilcoxon_test,
                              get_reaction_consistencies,
                              get_metareactions, amino_acid_metab)
```

Load GAM-clustering modules for comparison

In [5]:
```
# Read m1.tsv and m2.tsv
m1_df = pd.read_csv('m.1.genes.tsv', sep='\t')
m2_df = pd.read_csv('m.2.genes.tsv', sep='\t')
m3_df = pd.read_csv('m.3.genes.tsv', sep='\t')
m1_set = set(m1_df['symbol'].dropna())
m2_set = set(m2_df['symbol'].dropna())
m3_set = set(m3_df['symbol'].dropna())
```

Data used for the analysis

1. Reaction scores calculated by Compass (output of Compass run)

In [6]:
```
reaction_penalties = pd.read_csv("reactions.tsv",
                                 sep="\t", index_col = 0)
reaction_penalties.iloc[0:1,0:5]
```

Out[6]:

	Pat1_Ctrl_1_2	Pat1_Ctrl_1_3	Pat1_Ctrl_1_4	Pat1_Ctrl_1_5	Pat1_Ctrl_1_6
**10FTHF5GLUtl_pos**	4716.625141	4631.545613	4438.191038	4621.118757	4742.993507

2. Metadata required to split cells into groups

In [7]:
```
cell_metadata = pd.read_csv("meta.tsv",
                            index_col = 0, sep = "\t")
cell_metadata.head(2)
```

Out[7]:

	Diagnosis
**cell_id**	
**Pat1_Ctrl_1_2**	Ctrl
**Pat1_Ctrl_1_3**	Ctrl

3. Metadata for reaction provided by authors

In [8]:
```
reaction_metadata = pd.read_csv("reaction_metadata.csv", index_col = 0)
reaction_metadata.loc[['r0281']].iloc[:,0:2]
```

Out[8]:

	reaction_name	formula
**reaction_no_direction**		
**r0281**	Putrescine:oxygen oxidoreductase (deaminating)...	1.00 * Water [e] + 1.00 * O2 [e] + 1.00 * Putr...

In [9]:
```
reaction_metadata.loc[['r0281']].iloc[:,2:]
```

Out[9]:

	associated_genes	subsystem	EC_number	confidence
**reaction_no_direction**				
**r0281**	AOC1	Methionine and cysteine metabolism	1.4.3.6	0.0

Comparison of glioma IDHwt and IDHmut samples

Select groups

In [19]:
```
g1 = "IDHwt_GBM"
g2 = "IDHmut_GBM"
```

In [20]:
```
G1_cells = cell_metadata.index[cell_metadata['Diagnosis'] == g1]
G2_cells = cell_metadata.index[cell_metadata['Diagnosis'] == g2]
```

Calculate reaction scores

In [21]:
```
reaction_consistencies = get_reaction_consistencies(reaction_penalties)
```

Wilcoxon rank-sum test

Positive values indicate higher potential activity in Group2 (IDWmut)

In [22]:
```
wilcox_results = wilcoxon_test(reaction_consistencies, G2_cells, G1_cells)
wilcox_results['metadata_r_id'] = ""
for r in wilcox_results.index:
    if r in reaction_metadata.index:
        wilcox_results.loc[r, 'metadata_r_id'] = r
    elif r[:-4] in reaction_metadata.index:
        wilcox_results.loc[r, 'metadata_r_id'] = r[:-4]
    else:
        print("Should not occur")
```

Metareaction analysis

Join according to the reaction metadata

Join the metadata to the reactions in a new dataframe W, so that we can filter out non-core reactions
[Remove reactions with confidence other than 0 or 4 (4 = most confident; 0 = unassigned confidence) + filter out reactions in the citric acid cycle subsystem which are outside of the mitochondria]

In [23]:
```
W = join_wilcox_reaction_metadata(wilcox_results)
```

The “_neg” or “_pos” suffixes indicate the direction of a bidirectional reaction
“_pos” direction indicates positive flux through the reaction

Subset subsystems and plot distance

- Remove Miscellaneous and Unassigned
- Remove Transport, Exchange (and Other)
- Keep only subsystems with 5 or more reactions

In [24]:
```
data = filter_results(W)
```

Plot only top 25% for both groups

In [25]:
```
plot_reactions_top25(data,9,8)
```

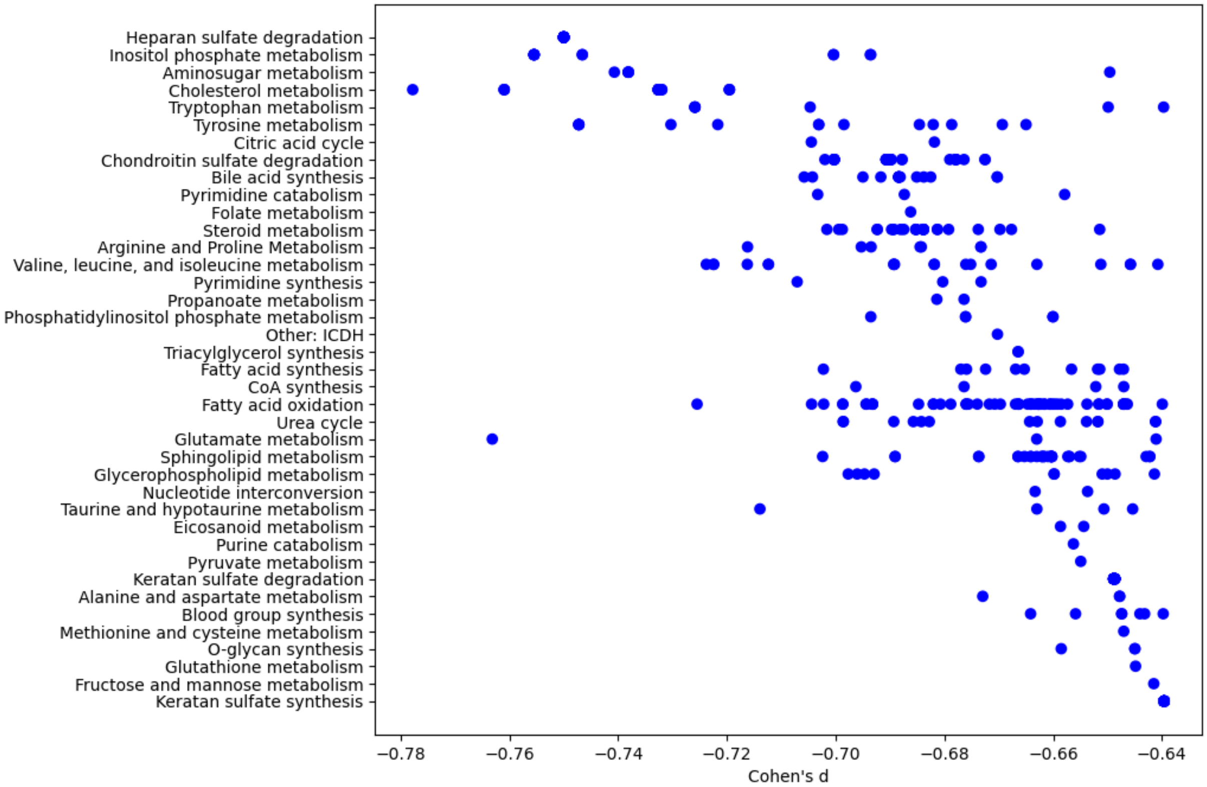

Up-regulated in IDHwt samples: Fatty acid, Energy, Amino acids metabolism, IP metabolism, Cholesterol metabolism, Lipid metabolism, Gluthathione metabolism, Urea cycle

Compare with GAM-clustering modules

Intersection of group-specific genes with GAM-clustering modules genes

In [26]:
```
pos_genes, neg_genes = get_top25_genes(data)
pos_genes_set = set(pos_genes)
neg_genes_set = set(neg_genes)
```

```
Number of positive-associated genes: 0
Number of negative-associated genes: 314
```

In [29]:
```
set_names = ['GAMclust_M1', 'GAMclust_M2', 'GAMclust_M3', 'IDHwt_GBM']
all_elems = m1_set.union(m2_set).union(m3_set).union(neg_genes_set)
df = pd.DataFrame([[e in m1_set, e in m2_set, e in m3_set, e in
neg_genes_set]
                   for e in all_elems], columns = set_names)
df_up = df.groupby(set_names).size()
upset = UpSet(df_up, show_counts=True)
upset.style_categories(
    "IDHwt_GBM",
```

```
    shading_facecolor="lavender"
)
upset.plot()
plt.show()
```

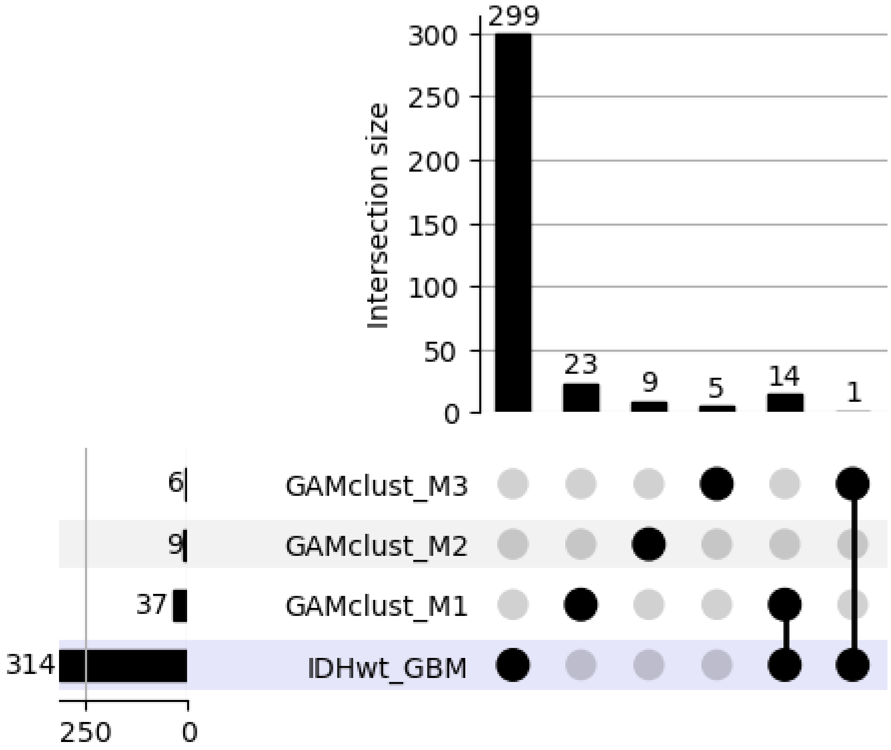

In [30]:
```
intersection_gam(pos_genes_set, neg_genes_set, m1_set, m2_set, m3_set)
```

```
Shared genes:
IDHwt_GBM ∩ GAMclust_M1 (14 genes): ACSL6, ALDH2, ALDH5A1, CPT1A, DGKH,
ELOVL6, GNE, IDH2, INPP5B, KDSR, PIK3C3, PLCE1, SDHC, SPTLC1
IDHwt_GBM ∩ GAMclust_M3 (1 genes): ASAH1
```

IDHwt vs M1:

Fatty acid oxidation, **TCA, Sphingolipid metabolism**, Aminosugar metabolism, Glycerophospholipid metabolism, IP metabolism, PIP metabolism, Arginine and Proline Metabolism, Glutamate metabolism, Valine, leucine, and isoleucine metabolism

Intersection of group-specific genes with GAM-clustering modules genes on a subsystem level

In [32]:
```
intersection_by_subsystem(data, m1_set, m2_set, m3_set)
```

```
--- Intersections for IDHwt_GBM Reactions ---
Subsystem: Sphingolipid metabolism
  ∩ M1 (2): KDSR, SPTLC1
  ∩ M3 (1): ASAH1

Subsystem: Glutamate metabolism
  ∩ M1 (1): ALDH5A1

Subsystem: Valine, leucine, and isoleucine metabolism
  ∩ M1 (1): ALDH2

Subsystem: Aminosugar metabolism
  ∩ M1 (1): GNE

Subsystem: Phosphatidylinositol phosphate metabolism
  ∩ M1 (1): PIK3C3

Subsystem: Fatty acid oxidation
  ∩ M1 (2): ACSL6, CPT1A

Subsystem: Glycerophospholipid metabolism
  ∩ M1 (1): DGKH

Subsystem: Fatty acid synthesis
  ∩ M1 (1): ELOVL6

Subsystem: Citric acid cycle
  ∩ M1 (2): IDH2, SDHC

Subsystem: Inositol phosphate metabolism
  ∩ M1 (2): INPP5B, PLCE1


--- Unique subsystems (no intersection with GAMclust_M1, GAMclust_M2 or
GAMclust_M3) ---
Total: 29
- Alanine and aspartate metabolism
- Arginine and Proline Metabolism
- Bile acid synthesis
- Blood group synthesis
- Cholesterol metabolism
- Chondroitin sulfate degradation
- CoA synthesis
- Eicosanoid metabolism
- Folate metabolism
- Fructose and mannose metabolism
- Glutathione metabolism
- Heparan sulfate degradation
- Keratan sulfate degradation
- Keratan sulfate synthesis
- Methionine and cysteine metabolism
- Nucleotide interconversion
- O-glycan synthesis
- Other: ICDH
- Propanoate metabolism
- Purine catabolism
```

```
- Pyrimidine catabolism
- Pyrimidine synthesis
- Pyruvate metabolism
- Steroid metabolism
- Taurine and hypotaurine metabolism
- Triacylglycerol synthesis
- Tryptophan metabolism
- Tyrosine metabolism
- Urea cycle
```

Comparison of control and glioma samples

Select groups

In [33]:
```
g1 = "Ctrl"
g2 = "IDH_GBM"
```

In [34]:
```
G1_cells = cell_metadata.index[cell_metadata['Diagnosis'] == g1]
G2_cells = cell_metadata.index[cell_metadata['Diagnosis'].isin(
    ['IDHwt_GBM', 'IDHmut_GBM'])]
```

Calculate reaction scores

In [35]:
```
reaction_consistencies = get_reaction_consistencies(reaction_penalties)
```

Wilcoxon rank-sum test

Positive values indicate higher potential activity in Group2 (GBM)

In [36]:
```
wilcox_results = wilcoxon_test(reaction_consistencies, G2_cells, G1_cells)
wilcox_results['metadata_r_id'] = ""
for r in wilcox_results.index:
    if r in reaction_metadata.index:
        wilcox_results.loc[r, 'metadata_r_id'] = r
    elif r[:-4] in reaction_metadata.index:
        wilcox_results.loc[r, 'metadata_r_id'] = r[:-4]
    else:
        print("Should not occur")
```

Metareaction analysis

Join according to the reaction metadata

In [37]:
```
W = join_wilcox_reaction_metadata(wilcox_results)
```

Subset subsystems and plot distance

In [38]:
```
data = filter_results(W)
```

In [41]:
```
plot_reactions(data,10,12)
```

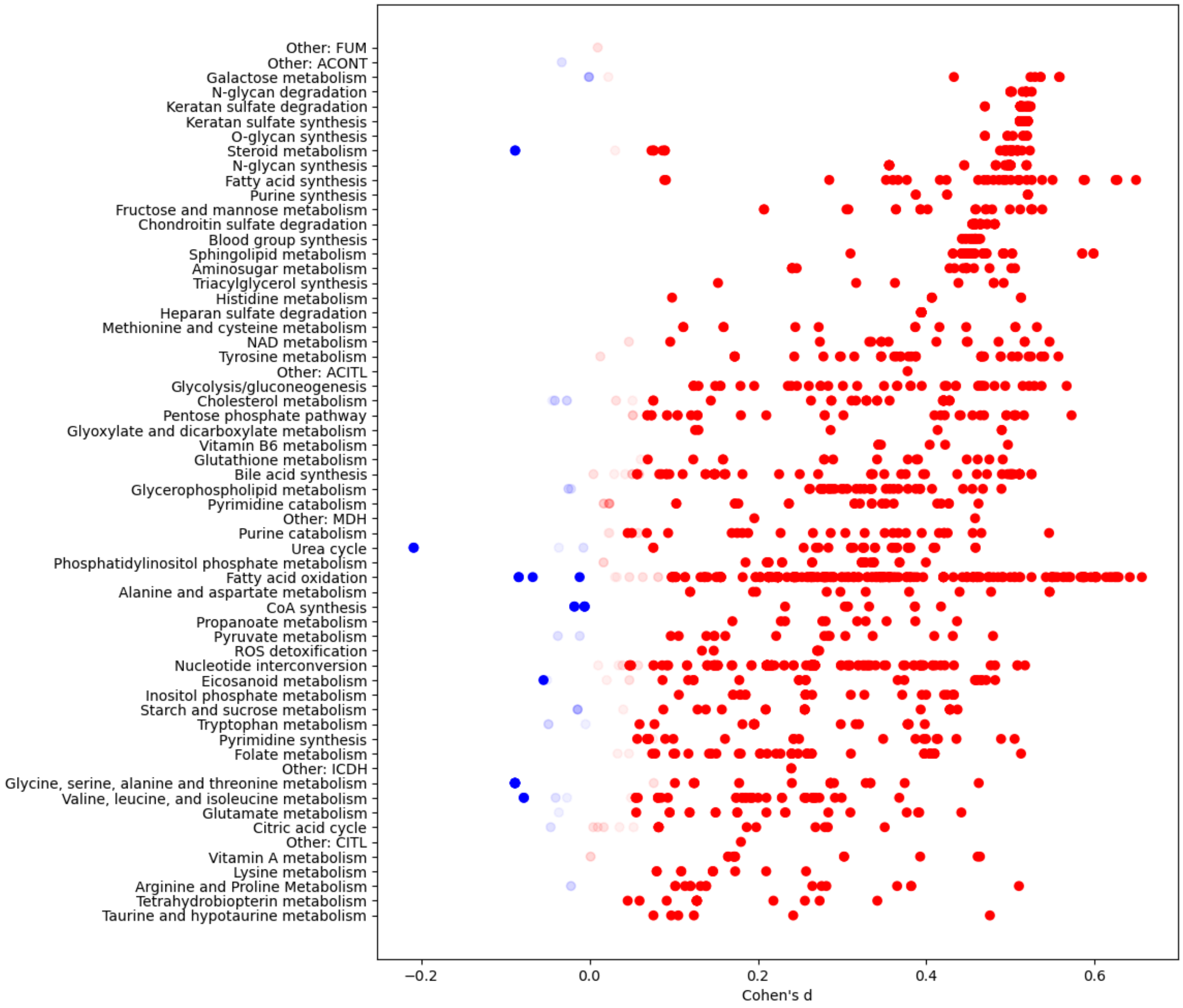

Plot only top 25% for both groups

In [42]:
```
plot_reactions_top25(data,9,8)
```

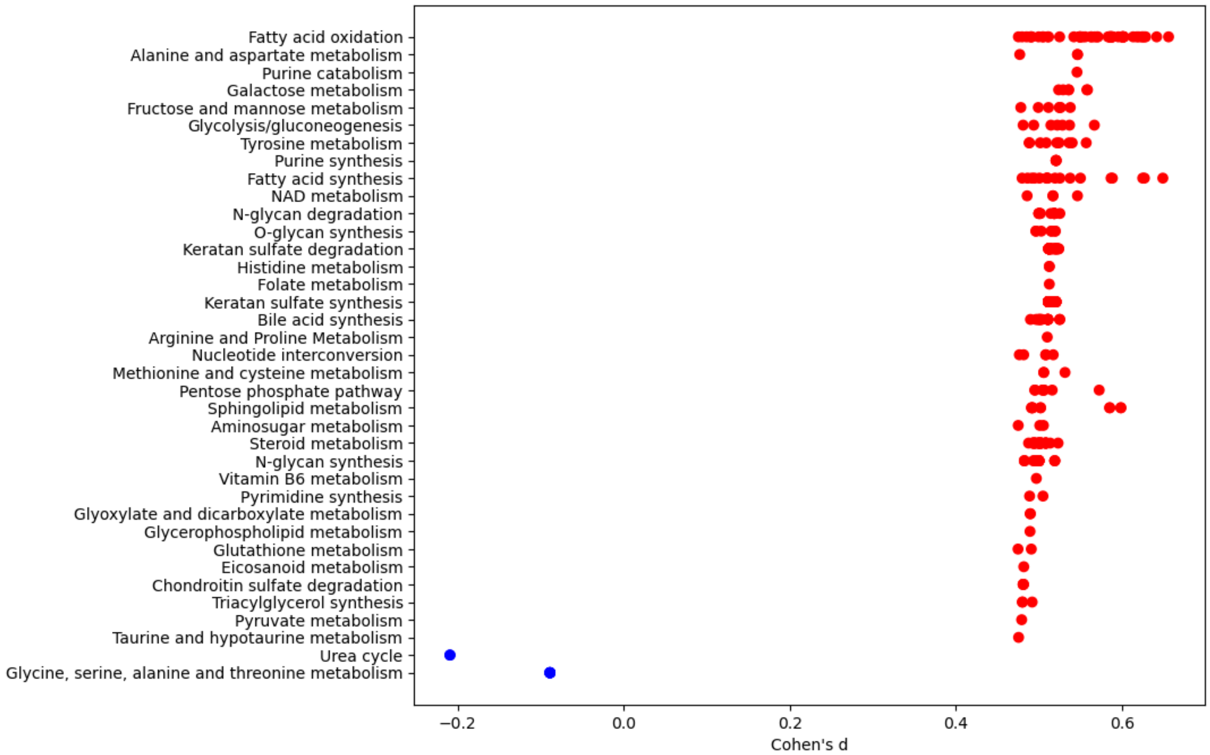

Down-regulated in tumor samples: Urea cycle, Gly/Ser/Ala/Thr metabolism
Up-regulated in tumor samples: Fatty acid, Energy (Glycolysis), Amino acids, Lipid metabolism, Vitamin B6, Chondroitine and Keratan sulfate synthesis, Gluthathione metabolism

Compare with GAM-clustering modules

Intersection of group-specific genes with GAM-clustering modules genes

In [43]:
```
pos_genes, neg_genes = get_top25_genes(data)
pos_genes_set = set(pos_genes)
neg_genes_set = set(neg_genes)
```

```
Number of positive-associated genes: 239
Number of negative-associated genes: 8
```

In [44]:
```
set_names = ['GAMclust_M1', 'GAMclust_M2', 'GAMclust_M3', 'IDH_GBM',
'Ctrl']
all_elems =
m1_set.union(m2_set).union(m3_set).union(pos_genes_set).union(neg_genes_set
)
df = pd.DataFrame([[e in m1_set, e in m2_set, e in m3_set, e in
pos_genes_set, e in neg_genes_set]
                   for e in all_elems], columns = set_names)
df_up = df.groupby(set_names).size()
upset = UpSet(df_up, show_counts=True)
upset.style_categories(
    "IDH_GBM",
    shading_facecolor="lavender"
```

```
)
upset.style_categories(
    "Ctrl",
    shading_facecolor="#d1ffbd"
)
upset.plot()
plt.show()
```

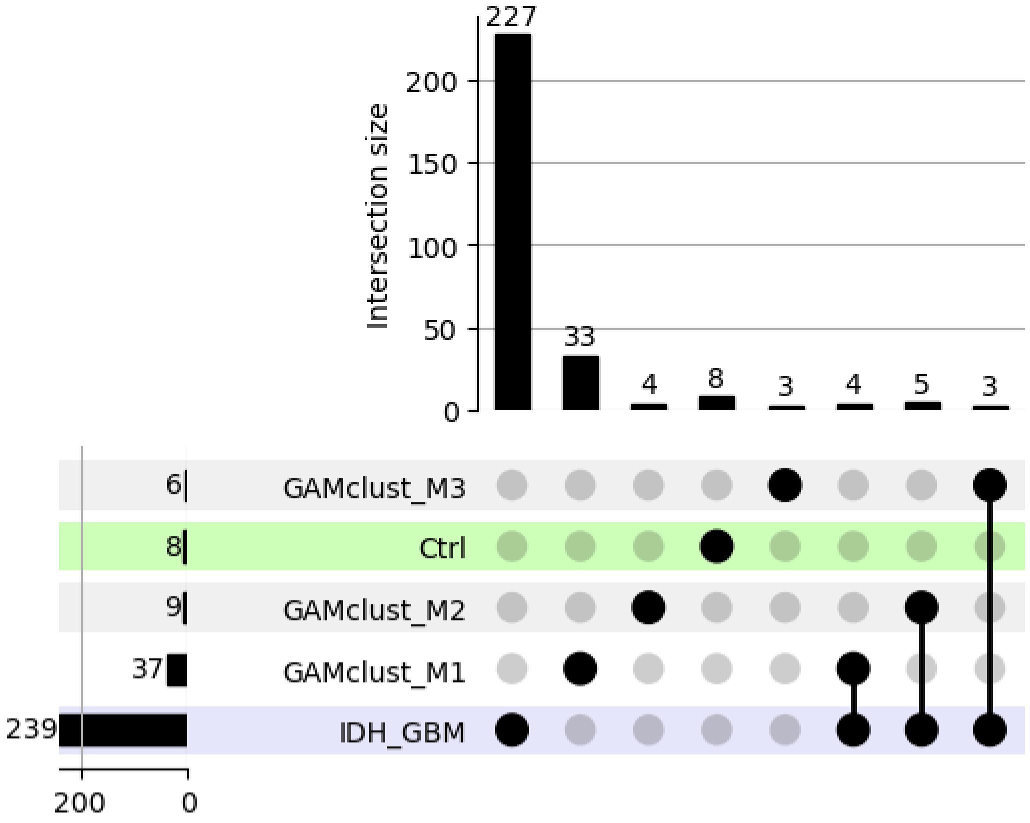

In [45]:
```
intersection_gam(pos_genes_set, neg_genes_set, m1_set, m2_set, m3_set)
```

```
Shared genes:
IDH_GBM ∩ GAMclust_M1 (4 genes): ACSL6, B3GNT6, ELOVL6, NANS
IDH_GBM ∩ GAMclust_M2 (5 genes): ENO1, LDHA, PGAM1, PKM, TPI1
IDH_GBM ∩ GAMclust_M3 (3 genes): ASAH1, LPL, UGCG
```

GBM vs M1:

Fatty acid oxidation, O-glycan synthesis

GBM vs M2:

Glycolysis/gluconeogenesis, Methionine and cysteine metabolism, Pyrimidine synthesis

GBM vs M3:

Sphingolipid metabolism

Intersection of group-specific genes with GAM-clustering modules genes on a subsystem level

In [46]:

```
intersection_by_subsystem(data, m1_set, m2_set, m3_set)
```

--- Intersections for IDH_GBM Reactions ---
Subsystem: Sphingolipid metabolism
  ∩ M3 (2): ASAH1, UGCG

Subsystem: Aminosugar metabolism
  ∩ M1 (1): NANS

Subsystem: Triacylglycerol synthesis
  ∩ M3 (1): LPL

Subsystem: Pyrimidine synthesis
  ∩ M2 (1): PKM

Subsystem: O-glycan synthesis
  ∩ M1 (1): B3GNT6

Subsystem: Methionine and cysteine metabolism
  ∩ M2 (1): LDHA

Subsystem: Fatty acid synthesis
  ∩ M1 (1): ELOVL6

Subsystem: Glycolysis/gluconeogenesis
  ∩ M2 (3): ENO1, PGAM1, TPI1

Subsystem: Fatty acid oxidation
  ∩ M1 (1): ACSL6

--- Unique subsystems (no intersection with GAMclust_M1, GAMclust_M2 or GAMclust_M3) ---
Total: 28
- Alanine and aspartate metabolism
- Arginine and Proline Metabolism
- Bile acid synthesis
- Chondroitin sulfate degradation
- Eicosanoid metabolism
- Folate metabolism
- Fructose and mannose metabolism
- Galactose metabolism
- Glutathione metabolism
- Glycerophospholipid metabolism
- Glycine, serine, alanine and threonine metabolism
- Glyoxylate and dicarboxylate metabolism
- Histidine metabolism
- Keratan sulfate degradation
- Keratan sulfate synthesis
- N-glycan degradation
- N-glycan synthesis
- NAD metabolism
- Nucleotide interconversion
- Pentose phosphate pathway
- Purine catabolism
- Purine synthesis
- Pyruvate metabolism
- Steroid metabolism

```
- Taurine and hypotaurine metabolism
- Tyrosine metabolism
- Urea cycle
- Vitamin B6 metabolism
```

Comparison of control and IDHwt glioma samples

Select groups

In [47]:
```
g1 = "Ctrl"
g2 = "IDHwt_GBM"
```

In [48]:
```
G1_cells = cell_metadata.index[cell_metadata['Diagnosis'] == g1]
G2_cells = cell_metadata.index[cell_metadata['Diagnosis'] == g2]
```

Calculate reaction scores

In [49]:
```
reaction_consistencies = get_reaction_consistencies(reaction_penalties)
```

Wilcoxon rank-sum test

Positive values indicate higher potential activity in Group2 (IDHwt)

In [50]:
```
wilcox_results = wilcoxon_test(reaction_consistencies, G2_cells, G1_cells)
wilcox_results['metadata_r_id'] = ""
for r in wilcox_results.index:
    if r in reaction_metadata.index:
        wilcox_results.loc[r, 'metadata_r_id'] = r
    elif r[:-4] in reaction_metadata.index:
        wilcox_results.loc[r, 'metadata_r_id'] = r[:-4]
    else:
        print("Should not occur")
```

Metareaction analysis

Join according to the reaction metadata

In [51]:
```
W = join_wilcox_reaction_metadata(wilcox_results)
```

Subset subsystems and plot distance

```
In [52]: data = filter_results(W)
```

```
In [53]: plot_reactions(data,10,12)
```

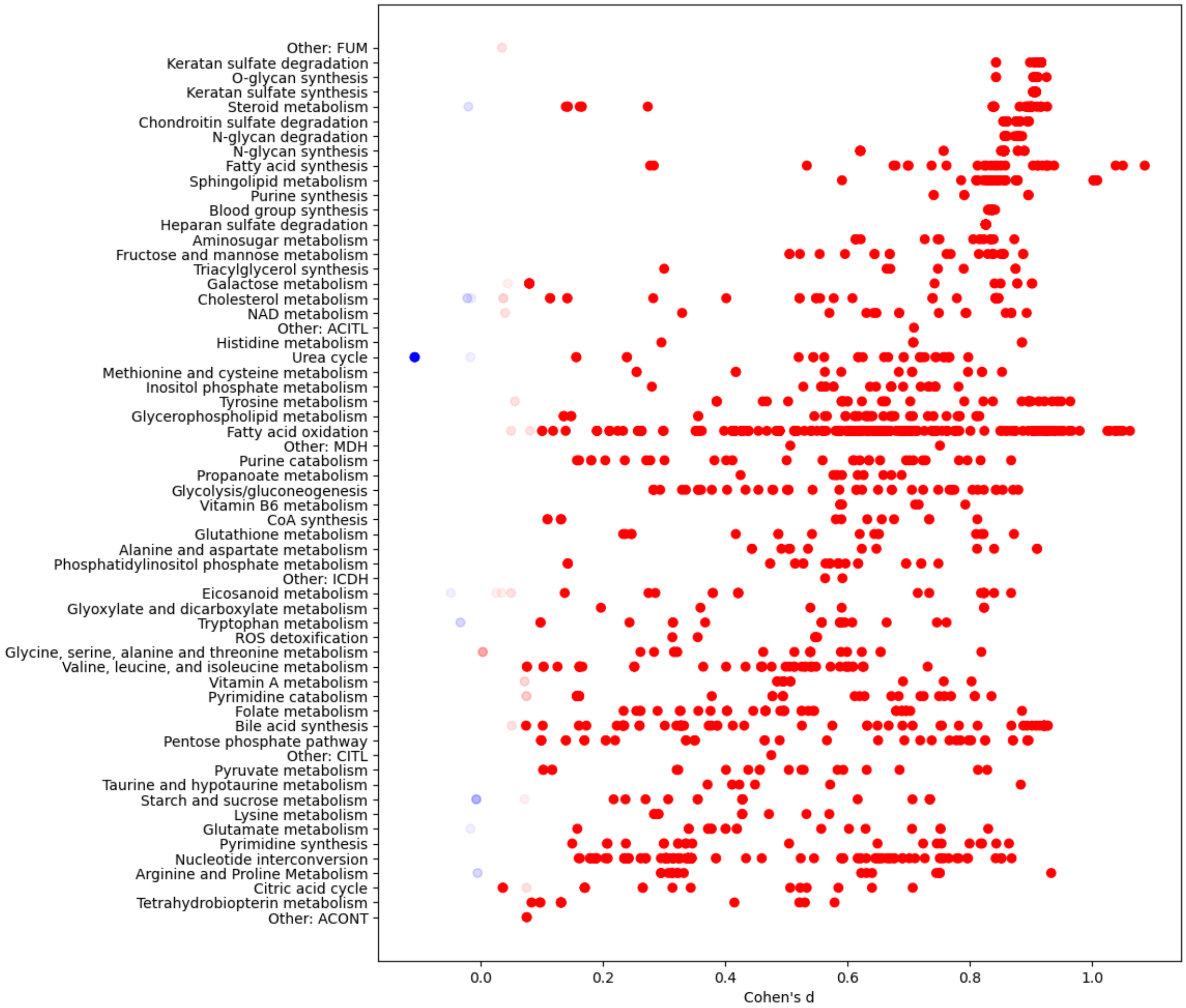

Plot only top 25% for both groups

```
In [54]: plot_reactions_top25(data,9,8)
```

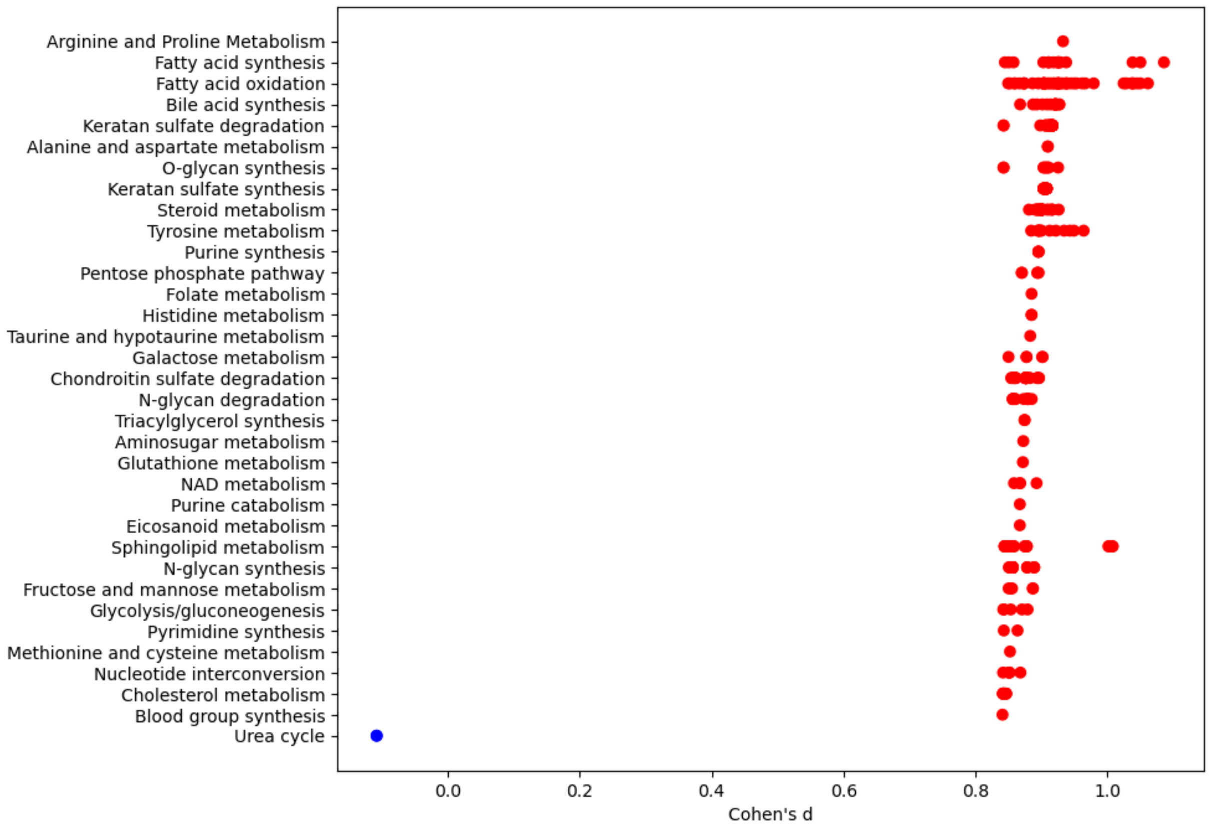

Down-regulated in IDHwt samples: Urea cycle

Up-regulated in IDHwt samples: Fatty acid, Energy, Aminoacids, Lipid metabolism, Vitamin B6, Chondroitine and Keratan sulfate synthesis, Gluthathione metabolism

Compare with GAM-clustering modules

Intersection of group-specific genes with GAM-clustering modules genes

In [55]:
```
pos_genes, neg_genes = get_top25_genes(data)
pos_genes_set = set(pos_genes)
neg_genes_set = set(neg_genes)
```

```
Number of positive-associated genes: 256
Number of negative-associated genes: 4
```

In [56]:
```
set_names = ['GAMclust_M1', 'GAMclust_M2', 'GAMclust_M3', 'IDHwt_GBM',
'Ctrl']
all_elems =
m1_set.union(m2_set).union(m3_set).union(pos_genes_set).union(neg_genes_set
)
df = pd.DataFrame([[e in m1_set, e in m2_set, e in m3_set, e in
pos_genes_set, e in neg_genes_set]
                   for e in all_elems], columns = set_names)
df_up = df.groupby(set_names).size()
upset = UpSet(df_up, show_counts=True)
upset.style_categories(
```

```
    "IDHwt_GBM",
    shading_facecolor="lavender"
)
upset.style_categories(
    "Ctrl",
    shading_facecolor="#d1ffbd"
)
upset.plot()
plt.show()
```

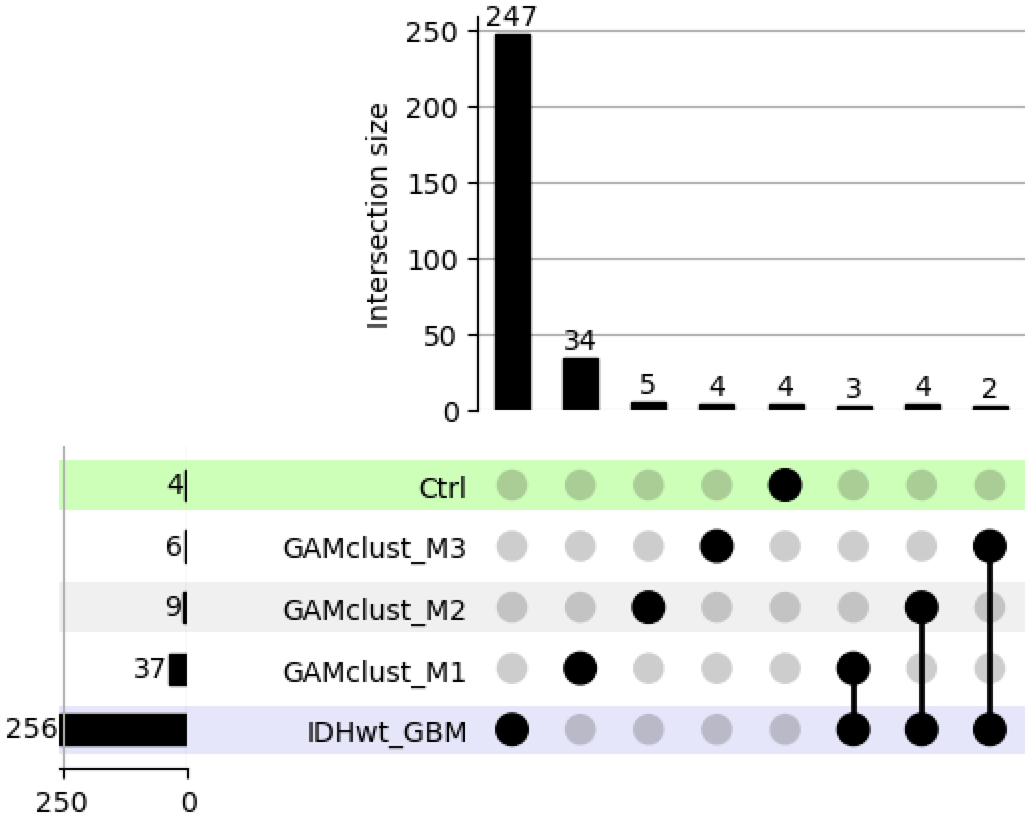

In [57]:
```
intersection_gam(pos_genes_set, neg_genes_set, m1_set, m2_set, m3_set)
```

```
Shared genes:
IDHwt_GBM ∩ GAMclust_M1 (3 genes): ACSL6, B3GNT6, ELOVL6
IDHwt_GBM ∩ GAMclust_M2 (4 genes): ENO1, LDHA, PGAM1, TPI1
IDHwt_GBM ∩ GAMclust_M3 (2 genes): ASAH1, UGCG
```

IDHwt vs M1:

Fatty acid oxidation O-glycan synthesis, **Sphingolipid metabolism**

IDHwt vs M2:

Glycolysis/gluconeogenesis, Methionine and cysteine metabolism

IDHwt vs M3:

Sphingolipid metabolism

Intersection of group-specific genes with GAM-clustering modules genes on a subsystem level

In [58]:
```
intersection_by_subsystem(data, m1_set, m2_set, m3_set)
```

```
--- Intersections for IDHwt_GBM Reactions ---
Subsystem: Sphingolipid metabolism
  ∩ M3 (2): ASAH1, UGCG

Subsystem: O-glycan synthesis
  ∩ M1 (1): B3GNT6

Subsystem: Fatty acid synthesis
  ∩ M1 (1): ELOVL6

Subsystem: Glycolysis/gluconeogenesis
  ∩ M2 (3): ENO1, PGAM1, TPI1

Subsystem: Fatty acid oxidation
  ∩ M1 (1): ACSL6

Subsystem: Methionine and cysteine metabolism
  ∩ M2 (1): LDHA


--- Unique subsystems (no intersection with GAMclust_M1, GAMclust_M2 or
GAMclust_M3) ---
Total: 28
- Alanine and aspartate metabolism
- Aminosugar metabolism
- Arginine and Proline Metabolism
- Bile acid synthesis
- Blood group synthesis
- Cholesterol metabolism
- Chondroitin sulfate degradation
- Eicosanoid metabolism
- Folate metabolism
- Fructose and mannose metabolism
- Galactose metabolism
- Glutathione metabolism
- Histidine metabolism
- Keratan sulfate degradation
- Keratan sulfate synthesis
- N-glycan degradation
- N-glycan synthesis
- NAD metabolism
- Nucleotide interconversion
- Pentose phosphate pathway
- Purine catabolism
- Purine synthesis
- Pyrimidine synthesis
- Steroid metabolism
- Taurine and hypotaurine metabolism
- Triacylglycerol synthesis
- Tyrosine metabolism
- Urea cycle
```

Comparison of control and IDHmut glioma samples

Select groups

In [59]:
```
g1 = "Ctrl"
g2 = "IDHmut_GBM"
```

In [60]:
```
G1_cells = cell_metadata.index[cell_metadata['Diagnosis'] == g1]
G2_cells = cell_metadata.index[cell_metadata['Diagnosis'] == g2]
```

Calculate reaction scores

In [61]:
```
reaction_consistencies = get_reaction_consistencies(reaction_penalties)
```

Wilcoxon rank-sum test

Positive values indicate higher potential activity in Group2 (IDWmut)

In [62]:
```
wilcox_results = wilcoxon_test(reaction_consistencies, G2_cells, G1_cells)
wilcox_results['metadata_r_id'] = ""
for r in wilcox_results.index:
    if r in reaction_metadata.index:
        wilcox_results.loc[r, 'metadata_r_id'] = r
    elif r[:-4] in reaction_metadata.index:
        wilcox_results.loc[r, 'metadata_r_id'] = r[:-4]
    else:
        print("Should not occur")
```

Metareaction analysis

Join according to the reaction metadata

In [63]:
```
W = join_wilcox_reaction_metadata(wilcox_results)
```

Subset subsystems and plot distance

In [64]:
```
data = filter_results(W)
```

In [65]:
```
plot_reactions(data,10,12)
```

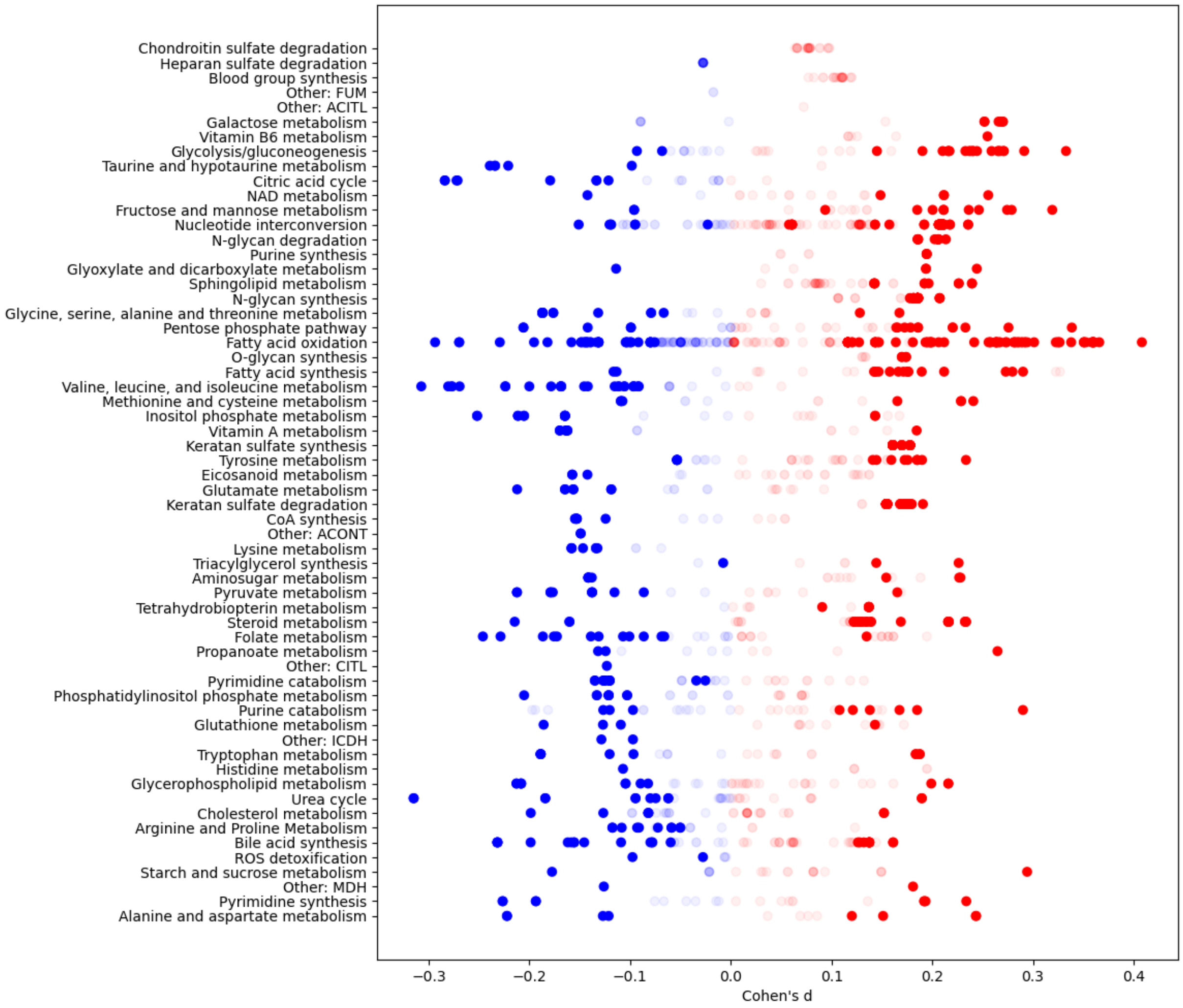

Plot only top 25% for both groups

In [66]:
```
plot_reactions_top25(data,9,8)
```

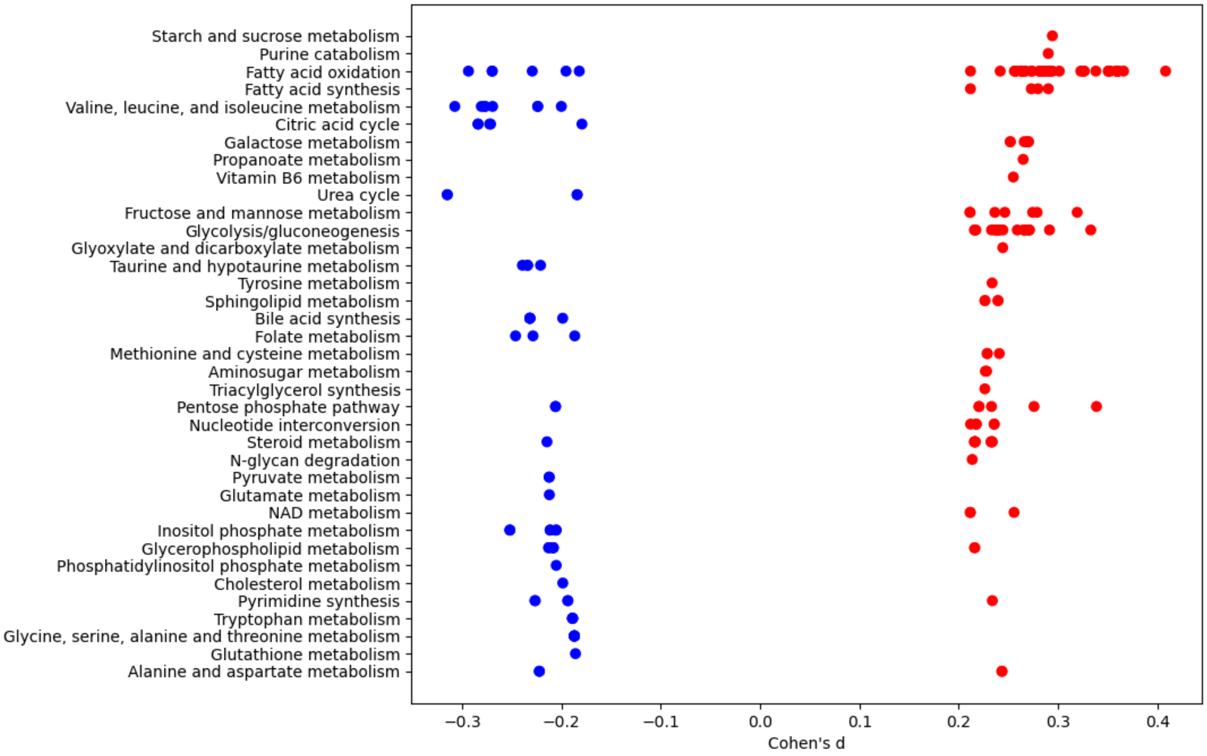

Down-regulated in IDHmut samples: Urea cycle, Fatty acid and Amino acids metabolism, TCA an PPP, IP metabolism, Cholesterol metabolism, Lipid metabolism

Up-regulated in IDHmut samples: Fatty acid, Energy (Glucolysis), Amino acids, Lipid metabolism

Compare with GAM-clustering modules

Intersection of group-specific genes with GAM-clustering modules genes

In [67]:
```
pos_genes, neg_genes = get_top25_genes(data)
pos_genes_set = set(pos_genes)
neg_genes_set = set(neg_genes)
```

```
Number of positive-associated genes: 105
Number of negative-associated genes: 90
```

In [68]:
```
set_names = ['GAMclust_M1', 'GAMclust_M2', 'GAMclust_M3', 'IDHmut_GBM',
'Ctrl']
all_elems =
m1_set.union(m2_set).union(m3_set).union(pos_genes_set).union(neg_genes_set
)
df = pd.DataFrame([[e in m1_set, e in m2_set, e in m3_set, e in
pos_genes_set, e in neg_genes_set]
                   for e in all_elems], columns = set_names)
df_up = df.groupby(set_names).size()
upset = UpSet(df_up, show_counts=True)
upset.style_categories(
```

```
    "IDHmut_GBM",
    shading_facecolor="lavender"
)
upset.style_categories(
    "Ctrl",
    shading_facecolor="#d1ffbd"
)
upset.plot()
plt.show()
```

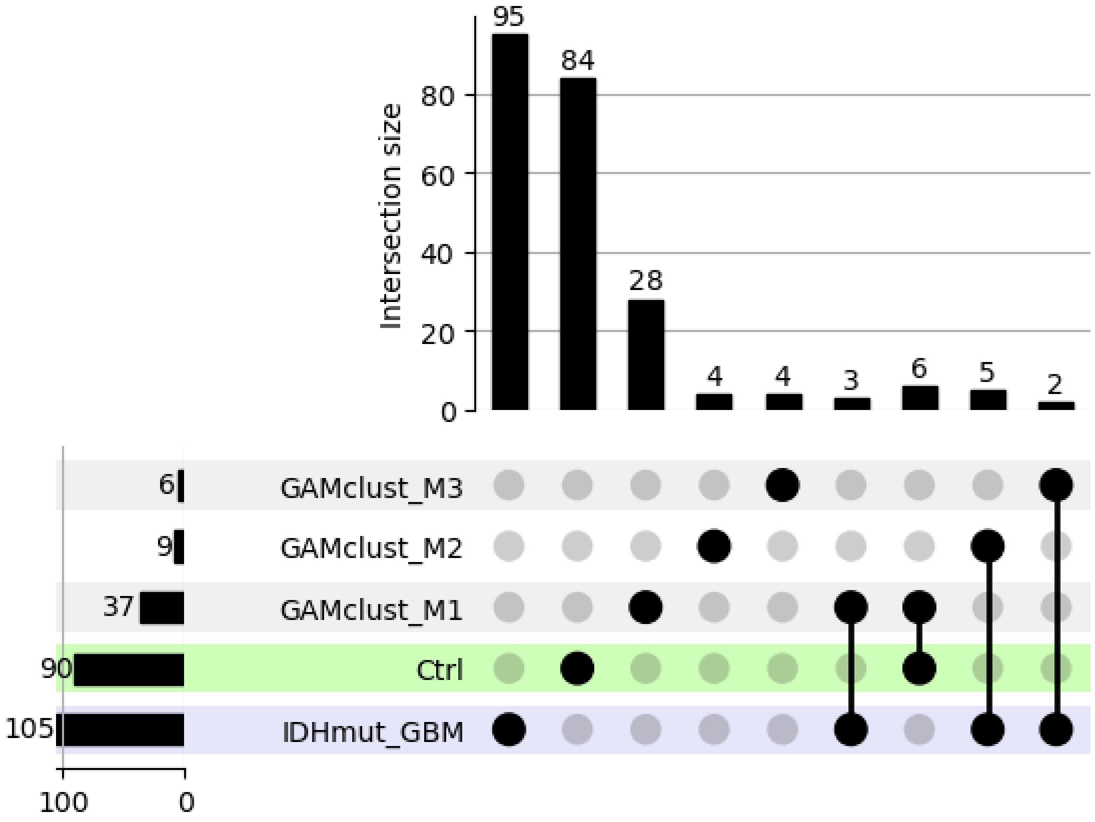

In [69]:
```
intersection_gam(pos_genes_set, neg_genes_set, m1_set, m2_set, m3_set)
```

```
Shared genes:
IDHmut_GBM ∩ GAMclust_M1 (3 genes): ACSL6, ELOVL6, NANS
Ctrl ∩ GAMclust_M1 (6 genes): ALDH2, CPT1A, IDH2, INPP5B, PLCE1, SDHC
IDHmut_GBM ∩ GAMclust_M2 (5 genes): ENO1, LDHA, PGAM1, PKM, TPI1
IDHmut_GBM ∩ GAMclust_M3 (2 genes): ASAH1, LPL
```

<u>Ctrl vs M1:</u>

Fatty acid oxidation, **TCA**, IP metabolism, Bile acid synthesis

<u>IDHmut vs M1:</u>

Fatty acid oxidation

<u>IDHmut vs M2:</u>

Glycolysis/gluconeogenesis, Pyrimidine synthesis

<u>IDHmut vs M3:</u>

Sphingolipid metabolism, Triacylglycerol synthesis

Intersection of group-specific genes with GAM-clustering modules genes on a subsystem level

In [70]:
```
intersection_by_subsystem(data, m1_set, m2_set, m3_set)
```

```
--- Intersections for IDHmut_GBM Reactions ---
Subsystem: Propanoate metabolism
  ∩ M2 (1): LDHA

Subsystem: Aminosugar metabolism
  ∩ M1 (1): NANS

Subsystem: Sphingolipid metabolism
  ∩ M3 (1): ASAH1

Subsystem: Methionine and cysteine metabolism
  ∩ M2 (1): LDHA

Subsystem: Fatty acid synthesis
  ∩ M1 (1): ELOVL6

Subsystem: Glycolysis/gluconeogenesis
  ∩ M2 (4): ENO1, LDHA, PGAM1, TPI1

Subsystem: Fatty acid oxidation
  ∩ M1 (1): ACSL6

Subsystem: Glyoxylate and dicarboxylate metabolism
  ∩ M2 (1): LDHA

Subsystem: Triacylglycerol synthesis
  ∩ M3 (1): LPL

Subsystem: Pyrimidine synthesis
  ∩ M2 (1): PKM


--- Intersections for Ctrl Reactions ---
Subsystem: Bile acid synthesis
  ∩ M1 (1): ALDH2

Subsystem: Fatty acid oxidation
  ∩ M1 (1): CPT1A

Subsystem: Citric acid cycle
  ∩ M1 (2): IDH2, SDHC

Subsystem: Inositol phosphate metabolism
  ∩ M1 (2): INPP5B, PLCE1


--- Unique subsystems (no intersection with GAMclust_M1, GAMclust_M2 or
GAMclust_M3) ---
Total: 25
- Alanine and aspartate metabolism
- Cholesterol metabolism
- Folate metabolism
- Fructose and mannose metabolism
- Galactose metabolism
- Glutamate metabolism
- Glutathione metabolism
```

```
- Glycerophospholipid metabolism
- Glycine, serine, alanine and threonine metabolism
- N-glycan degradation
- NAD metabolism
- Nucleotide interconversion
- Pentose phosphate pathway
- Phosphatidylinositol phosphate metabolism
- Purine catabolism
- Pyrimidine synthesis
- Pyruvate metabolism
- Starch and sucrose metabolism
- Steroid metabolism
- Taurine and hypotaurine metabolism
- Tryptophan metabolism
- Tyrosine metabolism
- Urea cycle
- Valine, leucine, and isoleucine metabolism
- Vitamin B6 metabolism
```

Discussion

Compass found a substantial list of reactions for each conditions. However, in all comparisons cells from IDHwt showed the highest metabolic potential in a variety of metabolic pathways including Urea cycle, Fatty acid, Energy, Amino acids, Gluthathione metabolism, IP metabolism, Cholesterol and Lipid metabolism. For both tumor types Urea cycle appeared to be downregulated but in comparison between tumors it is more active in IDHwt tumors.

Similar to GAM-clustering results among other pathways we noted higher activity of Glycolysis in tumor samples and downregulation of TCA in IDHmut compared to IDHwt.

Helper functions

Modified function from original code

In [10]:
```
def plot_differential_scores(data, title, c="#D3A991", n_top=5):
    plt.figure(figsize=(8,6))
    axs = plt.gca()
    axs.scatter(data['cohens_d'], -np.log10(data['adjusted_pval']), c=c)
    axs.set_xlabel("Cohen's d", fontsize=16)
    axs.set_ylabel("-log10 (Wilcoxon-adjusted p)", fontsize=16)
    #Everything after this should be tweaked depending on your application
    axs.set_xlim(-2.2, 2.2)
    axs.axvline(0, dashes=(3,3), c='black')
    axs.axhline(1, dashes=(3,3), c='black')
    axs.set_title(title, fontdict={'fontsize':20})
    axs.annotate('', xy=(0.5, -0.13), xycoords='axes fraction',
                 xytext=(0, -0.13),
            arrowprops=dict(arrowstyle="<-", color='#348C73', linewidth=4))
    axs.annotate(g2, xy=(0.75, -0.2), xycoords='axes fraction',
fontsize=16)
    axs.annotate('', xy=(0.5, -0.13), xycoords='axes fraction',
                 xytext=(1, -0.13),
            arrowprops=dict(arrowstyle="<-", color='#E92E87',
                            linewidth=4))
    axs.annotate(g1, xy=(0.25, -0.2), xycoords='axes fraction',
                 fontsize=16)
    # Sort and get top rows
    top_rows = data.sort_values('adjusted_pval').head(n_top)
    # Remove text within parentheses (and the parentheses themselves)
    top_rows['reaction_name'] = top_rows['reaction_name'].str.replace(
        r'\s*\(.*\)', '', regex=True)
    # Create dictionary with original indices
    top = top_rows['reaction_name'].to_dict()
    for r in data.index:
```

```python
        if r in top:
            x = data.loc[r, 'cohens_d']
            y = -np.log10(data.loc[r, 'adjusted_pval'])
            offset = (20, 0)
            if x < 0:
                offset = (-100, -40)
            axs.annotate(top[r], (x,y), xytext = offset,
                         textcoords='offset pixels',
                         arrowprops={'arrowstyle':"-"})
```

Add custom functions for plotting

In [11]:
```python
def join_wilcox_reaction_metadata(wilcox_results):
    W = wilcox_results.merge(reaction_metadata, how='left',
                             left_on='metadata_r_id', right_index=True,
                                 validate='m:1')
    W = W[W['confidence'].isin([0,4])]
    W = W[~W['EC_number'].isna()]
    W.loc[(W['formula'].map(lambda x: '[m]' not in x)) &
          (W['subsystem'] == "Citric acid cycle"), 'subsystem'] = 'Other'
    return(W)
```

In [12]:
```python
def filter_results(W):
    data = W[~W['subsystem'].isin(["Miscellaneous", "Unassigned"])]
    data = data[~data['subsystem'].map(
        lambda x: "Transport" in x or "Exchange" in x)]
    items, counts = np.unique(data['subsystem'], return_counts=True)
    items = [items[i] for i in range(len(items)) if counts[i] > 5]
    data = data[data['subsystem'].isin(items)]
    mask = data['subsystem'].str.contains('Other', case=False, na=False)
    data.loc[mask, 'subsystem'] = data.loc[mask, 'metadata_r_id'].apply(
        lambda x: 'Other: ' + ''.join([c for c in x if c.isupper()])
    )
    return(data)
```

In [40]:
```python
def plot_reactions(data, w=12, h=12):
    plt.figure(figsize=(w, h))
    axs = plt.gca()

    d = (
        data[data["adjusted_pval"] < 0.05]
        .groupby("subsystem")["cohens_d"]
        .median()
        .abs()
        .sort_values()
    )

    # invisible points only to set the order of subsystems on y-axis
    axs.scatter(d.values, d.index, alpha=0)

    color = data["cohens_d"].map(lambda x: "r" if x >= 0 else "b")
    alpha = data["adjusted_pval"].map(lambda x: 1.0 if x < 0.05 else 0.05)

    axs.scatter(
        data["cohens_d"],
```

```python
        data["subsystem"],
        c=color,
        alpha=alpha
    )

    axs.set_xlabel("Cohen's d")
    plt.show()
```

In [14]:

```python
def get_filtered_data_top25(data):
    # Filter significant data
    sig_data = data[data['adjusted_pval'] < 0.05].copy()

    # Separate negative and positive cohens_d values
    neg = sig_data[sig_data['cohens_d'] < 0]
    pos = sig_data[sig_data['cohens_d'] > 0]

    # Calculate quantiles
    neg_cutoff = neg['cohens_d'].quantile(0.25)
    pos_cutoff = pos['cohens_d'].quantile(0.75)

    # Filter bottom 25% negative and top 25% positive
    neg_bottom_25 = neg[neg['cohens_d'] <= neg_cutoff]
    pos_top_25 = pos[pos['cohens_d'] >= pos_cutoff]

    # Combine filtered data
    filtered = pd.concat([neg_bottom_25, pos_top_25])
    return(filtered)
```

In [15]:

```python
def plot_reactions_top25(data,w=12,h=12):

    filtered = get_filtered_data_top25(data)

    # Calculate median absolute cohens_d per subsystem for sorting
    d = filtered.groupby('subsystem')['cohens_d'].median().abs()
    sorted_subsystems = d.sort_values().index

    plt.figure(figsize=(w,h))
    axs = plt.gca()
    # Map colors and alpha
    color = filtered['cohens_d'].map(lambda x: 'r' if x >= 0 else 'b')
    alpha = filtered['adjusted_pval'].map(
        lambda x: 1.0 if x < 0.05 else 0.25)
    y_positions = {sub: i for i, sub in enumerate(sorted_subsystems)}
    y_vals = filtered['subsystem'].map(y_positions)
    axs.scatter(filtered['cohens_d'], y_vals, c=color, alpha=alpha)
    axs.set_yticks(range(len(sorted_subsystems)))
    axs.set_yticklabels(sorted_subsystems)
    axs.set_xlabel("Cohen's d")
    plt.show()
```

In [16]:

```python
def get_top25_genes(data):
    filtered = get_filtered_data_top25(data)
    pos_reactions = filtered[filtered['cohens_d'] > 0]
    neg_reactions = filtered[filtered['cohens_d'] < 0]
```

```python
    def extract_genes(df, col='associated_genes', delimiter='; '):
        genes = df[col].dropna().str.split(delimiter)
        return set(gene for sublist in genes for gene in sublist if gene)

    # Extract unique genes from each group
    pos_genes = extract_genes(pos_reactions)
    neg_genes = extract_genes(neg_reactions)

    pos_genes = sorted(pos_genes)
    neg_genes = sorted(neg_genes)

    # Show stats
    print(f"Number of positive-associated genes: {len(pos_genes)}")
    # print(pos_genes)
    print(f"Number of negative-associated genes: {len(neg_genes)}")
    # print(neg_genes)

    return(pos_genes, neg_genes)
```

In [17]:
```python
def intersection_gam(pos_genes_set, neg_genes_set, m1, m2, m3):
    m1_set = set(m1)
    m2_set = set(m2)
    m3_set = set(m3)

    gam_modules = {
        "GAMclust_M1": m1_set,
        "GAMclust_M2": m2_set,
        "GAMclust_M3": m3_set,
    }

    def format_genes(genes):
        return ", ".join(sorted(genes))

    print("\nShared genes:")

    for module_name, module_genes in gam_modules.items():
        inter_pos = pos_genes_set & module_genes
        inter_neg = neg_genes_set & module_genes

        if inter_pos:
            print(
                f"{g2} ∩ {module_name} ({len(inter_pos)} genes): "
                f"{format_genes(inter_pos)}"
            )

        if inter_neg:
            print(
                f"{g1} ∩ {module_name} ({len(inter_neg)} genes): "
                f"{format_genes(inter_neg)}"
            )
```

In [18]:
```python
def intersection_by_subsystem(data, m1, m2, m3):
    m1_set = set(m1)
    m2_set = set(m2)
    m3_set = set(m3)
```

```python
gam_modules = {
    "M1": m1_set,
    "M2": m2_set,
    "M3": m3_set,
}

filtered = get_filtered_data_top25(data)

positive_df = filtered[filtered["cohens_d"] > 0]
negative_df = filtered[filtered["cohens_d"] < 0]

def get_subsystem_gene_map(df):
    subsystem_genes = defaultdict(set)

    for _, row in df.iterrows():
        subsystem = row["subsystem"]
        genes = str(row["associated_genes"]).split(";")
        genes = [g.strip() for g in genes if g.strip()]
        subsystem_genes[subsystem].update(genes)

    return subsystem_genes

pos_subsystem_genes = get_subsystem_gene_map(positive_df)
neg_subsystem_genes = get_subsystem_gene_map(negative_df)

def print_intersections(group_name, subsystem_genes):
    print(f"\n--- Intersections for {group_name} ---")

    for subsystem, genes in subsystem_genes.items():
        intersections = {
            module_name: genes & module_genes
            for module_name, module_genes in gam_modules.items()
        }

        if any(intersections.values()):
            print(f"Subsystem: {subsystem}")

            for module_name, inter_genes in intersections.items():
                if inter_genes:
                    print(
                        f"  ∩ {module_name} ({len(inter_genes)}): "
                        f"{', '.join(sorted(inter_genes))}"
                    )

            print()

all_gam_genes = m1_set | m2_set | m3_set

pos_all_genes = {
    gene
    for genes in pos_subsystem_genes.values()
    for gene in genes
}

neg_all_genes = {
```

```
        gene
        for genes in neg_subsystem_genes.values()
        for gene in genes
    }

    if pos_all_genes & all_gam_genes:
        print_intersections(f"{g2} Reactions", pos_subsystem_genes)

    if neg_all_genes & all_gam_genes:
        print_intersections(f"{g1} Reactions", neg_subsystem_genes)

    all_subsystems = {**pos_subsystem_genes, **neg_subsystem_genes}

    non_intersecting_subsystems = []

    for subsystem, genes in all_subsystems.items():
        if not (genes & all_gam_genes):
            non_intersecting_subsystems.append(subsystem)

    print("\n--- Unique subsystems (no intersection with GAMclust_M1,
GAMclust_M2 or GAMclust_M3) ---")
    print(f"Total: {len(non_intersecting_subsystems)}")

    for subsystem in sorted(non_intersecting_subsystems):
        print(f"- {subsystem}")
```

In [ ]:

```
```

In [ ]:

```
```
